# Multiomics integration unveils photoperiodic plasticity in the molecular rhythms of marine phytoplankton

**DOI:** 10.1093/plcell/koaf033

**Published:** 2025-02-11

**Authors:** Ana B Romero-Losada, Christina Arvanitidou, M Elena García-Gómez, María Morales-Pineda, M José Castro-Pérez, Yen Peng Chew, Gerben van Ooijen, Mercedes García-González, Francisco J Romero-Campero

**Affiliations:** Institute for Plant Biochemistry and Photosynthesis, Universidad de Sevilla – Consejo Superior de Investigaciones Científicas, Av. Américo Vespucio 49, Seville 41092, Spain; Department of Computer Science and Artificial Intelligence, Universidad de Sevilla, Av. Reina Mercedes s/n, Seville 41012, Spain; Institute for Plant Biochemistry and Photosynthesis, Universidad de Sevilla – Consejo Superior de Investigaciones Científicas, Av. Américo Vespucio 49, Seville 41092, Spain; Department of Computer Science and Artificial Intelligence, Universidad de Sevilla, Av. Reina Mercedes s/n, Seville 41012, Spain; Institute for Plant Biochemistry and Photosynthesis, Universidad de Sevilla – Consejo Superior de Investigaciones Científicas, Av. Américo Vespucio 49, Seville 41092, Spain; Institute for Plant Biochemistry and Photosynthesis, Universidad de Sevilla – Consejo Superior de Investigaciones Científicas, Av. Américo Vespucio 49, Seville 41092, Spain; Institute for Biomedicine in Seville, Universidad de Sevilla – Consejo Superior de Investigaciones Científicas, Av. Manuel Siurot s/n, Seville 41012, Spain; School of Biological Sciences, University of Edinburgh, Max Born Crescent, Edinburgh EH9 3BF, UK; School of Biological Sciences, University of Edinburgh, Max Born Crescent, Edinburgh EH9 3BF, UK; Institute for Plant Biochemistry and Photosynthesis, Universidad de Sevilla – Consejo Superior de Investigaciones Científicas, Av. Américo Vespucio 49, Seville 41092, Spain; Institute for Plant Biochemistry and Photosynthesis, Universidad de Sevilla – Consejo Superior de Investigaciones Científicas, Av. Américo Vespucio 49, Seville 41092, Spain; Department of Computer Science and Artificial Intelligence, Universidad de Sevilla, Av. Reina Mercedes s/n, Seville 41012, Spain

## Abstract

Earth's tilted rotation and translation around the Sun produce pervasive rhythms on our planet, giving rise to photoperiodic changes in diel cycles. Although marine phytoplankton plays a key role in ecosystems, multiomics analysis of its responses to these periodic environmental signals remains largely unexplored. The marine picoalga *Ostreococcus tauri* was chosen as a model organism due to its cellular and genomic simplicity. *Ostreococcus* was subjected to different light regimes to investigate its responses to periodic environmental signals: long summer days, short winter days, constant light, and constant dark conditions. Although <5% of the transcriptome maintained oscillations under both constant conditions, 80% presented diel rhythmicity. A drastic reduction in diel rhythmicity was observed at the proteome level, with 39% of the detected proteins oscillating. Photoperiod-specific rhythms were identified for key physiological processes such as the cell cycle, photosynthesis, carotenoid biosynthesis, starch accumulation, and nitrate assimilation. In this study, a photoperiodic plastic global orchestration among transcriptome, proteome, and physiological dynamics was characterized to identify photoperiod-specific temporal offsets between the timing of transcripts, proteins, and physiological responses.

## Teaser

Marine phytoplankton exhibits a global orchestration among transcriptome, proteome, and physiological dynamics, marked by seasonal temporal offsets between transcript, protein, and physiological peaks.

## Introduction

Marine phytoplankton plays a pivotal role in Earth's ecosystems, acting as primary producers by contributing to ∼45% of global photosynthetic net primary production (Field et al. 1998). Consequently, it not only sustains the existence of most oceanic life but also supports life across the entire planet. Light availability has a profound impact on marine phytoplankton growth and physiology (Edwards et al. 2015). Earth's rotation produces the most pervasive rhythmic environmental signal, giving rise to alternating cycles of days (light periods or photoperiods) and nights (dark periods or skotoperiods), collectively known as diel cycles. Earth's tilted rotational axis and its translation around the Sun also lead to photoperiodic variations in diel cycles, resulting in long days (LDs) in summer and short days (SDs) in winter in temperate zones, the regions between the tropics and the polar circles. Seasonality plays a central regulatory role in marine phytoplankton dynamics (Bolaños et al. 2020; Chen et al. 2021; Mondal and Banerjee 2022). However, the different molecular rhythms underpinning these responses are not yet characterized.

Chronobiology is a multidisciplinary field that focuses on the study of the timing and regulation of biological rhythms and their synchronization with environmental cycles (Kuhlman et al. 2018). Specifically, the autonomous oscillating molecular systems that have evolved to anticipate and respond to diel cycles are referred to as circadian clocks. These systems are entrained to external inputs, diel cycles, and produce rhythmic output of biological processes. Circadian rhythms are self-sustained, maintaining rhythmicity of about 24 h under constant conditions. To distinguish these biological rhythms from diel rhythms that respond to the environment, chronobiology experiments are typically designed as a sequence of several consecutive days under alternating light/dark cycles, followed by several consecutive days of constant light (LL) or dark (DD), called free-running conditions (Kuhlman et al. 2018). Extensive chronobiological studies on circadian and diel rhythms have been conducted at the molecular and physiological levels in many model organisms (Bläsing et al. 2005; Miller et al. 2007; Patke et al. 2020; Ma et al. 2021; Petersen et al. 2022; Häfker et al. 2023) uncovering key molecular mechanisms and regulators controlling circadian rhythms. However, only initial steps have been taken to analyze such systems in marine phytoplankton using omics technologies (Monnier et al. 2010; Annunziata et al. 2019; Kay et al. 2021), which are beginning to characterize molecular rhythms at the transcriptomic and proteomic levels and identify their circadian clock regulators. A recent kingdom-wide comparative analysis of diel gene expression profiles across Archaeplastida has highlighted evolutionary trends in rhythmic patterns across the green lineage (Ferrari et al. 2019). However, this study solely focused on the transcriptomic layer and did not explore the effect of different photoperiods and free-running conditions.

To characterize the responses of marine phytoplankton to photoperiodic variations in diel cycles, the model phytoplanktonic picoeukaryote *Ostreococcus tauri* (*Ostreococcus*) was selected due to its cellular simplicity (Henderson et al. 2007) and fully sequenced and annotated genome (Derelle et al. 2006; Palenik et al. 2007; Blanc-Mathieu et al. 2014). Moreover, *Ostreococcus* occupies a key position in the green lineage (Viridiplantae) as a representative of the Class Mamiellophyceae, which diverged early from the group that would give rise to land plants and, therefore, could provide insights into the ancestral traits of green plants and their evolutionary processes (van Baren et al. 2016; de los Reyes et al. 2017; Bachy et al. 2022). Intensive and extensive studies on the physiology of *Ostreococcus* have provided a solid foundation for systems biology and omics studies. Chronobiological studies (Corellou et al. 2009; Monnier et al. 2010; O'-Neill et al. 2011; van Ooijen et al. 2011; Freeney et al. 2016) have uncovered the fundamental components of the *Ostreococcus* circadian clock revealing key rhythmic processes. Cell-cycle analysis (Corellou et al. 2005; Robbens et al. 2005; Gil-Rodríguez et al. 2024) have provided insights into the molecular components regulating cell-cycle progression in *Ostreococcus* under different light regimes while photobiology approaches (Sands et al. 2023) have highlighted the role of light quality in different biological processes. Research into sexual reproduction (Benites et al. 2021), viral infections dynamics (Derelle et al. 2018; Castillo et al. 2021), and biomass composition (Ral et al. 2004; Degraeve-Guilbault et al. 2017, 2020; Guyon et al. 2018) have expanded the knowledge of *Ostreococcus*’ potential strategies to cope with biotic and nutritional stresses. Finally, omics analysis (Monnier et al. 2010; Le Bihan et al. 2011, 2015; Henríquez-Castillo et al. 2018; Bousquet et al. 2020; Kay et al. 2021) have provided global characterizations of the transcriptome and proteome separately. These investigations have unveiled that the *Ostreococcus* circadian clock is notably simpler than that of other photosynthetic organisms like *Arabidopsis thaliana*. It has been shown to be constituted only by a reduced central loop comprising potential orthologues of CIRCADIAN CLOCK ASSOCIATED 1 (CCA1) and TIMING OF CAB EXPRESSION 1 (TOC1). In contrast, Arabidopsis has a more complex clock composed of interlocking loops, including a morning loop constituted among others by PSEUDO RESPONSE REGULATORS 9, 7, and 5 (PRR9, PRR7, and PRR5), the central loop consisting in CCA1 and TOC1, and an evening loop involving additional components such as ZEITLUPE and GIGANTEA (Serrano-Bueno et al. 2017).

Despite these advances, *Ostreococcus* responses to photoperiodic variations in diel cycles, as well as free-running conditions, remain to be explored, particularly through multiomics integrative analysis. This study aimed to characterize the *Ostreococcus* transcriptome, proteome, and physiological rhythmicity, under long and short photoperiods. Additionally, to determine circadian rhythms, our experiments were extended to free-running conditions consisting of LL or DD. Integration of transcriptomic and proteomic data with physiological measurements unveiled a photoperiodic plastic global orchestration between transcriptome, proteome, and physiological dynamics, with photoperiod-specific temporal offsets between the phase of transcript, protein, and physiological rhythms.

## Results

### Transcript rhythmicity under diel cycles and free-running conditions

**Figure 1.**
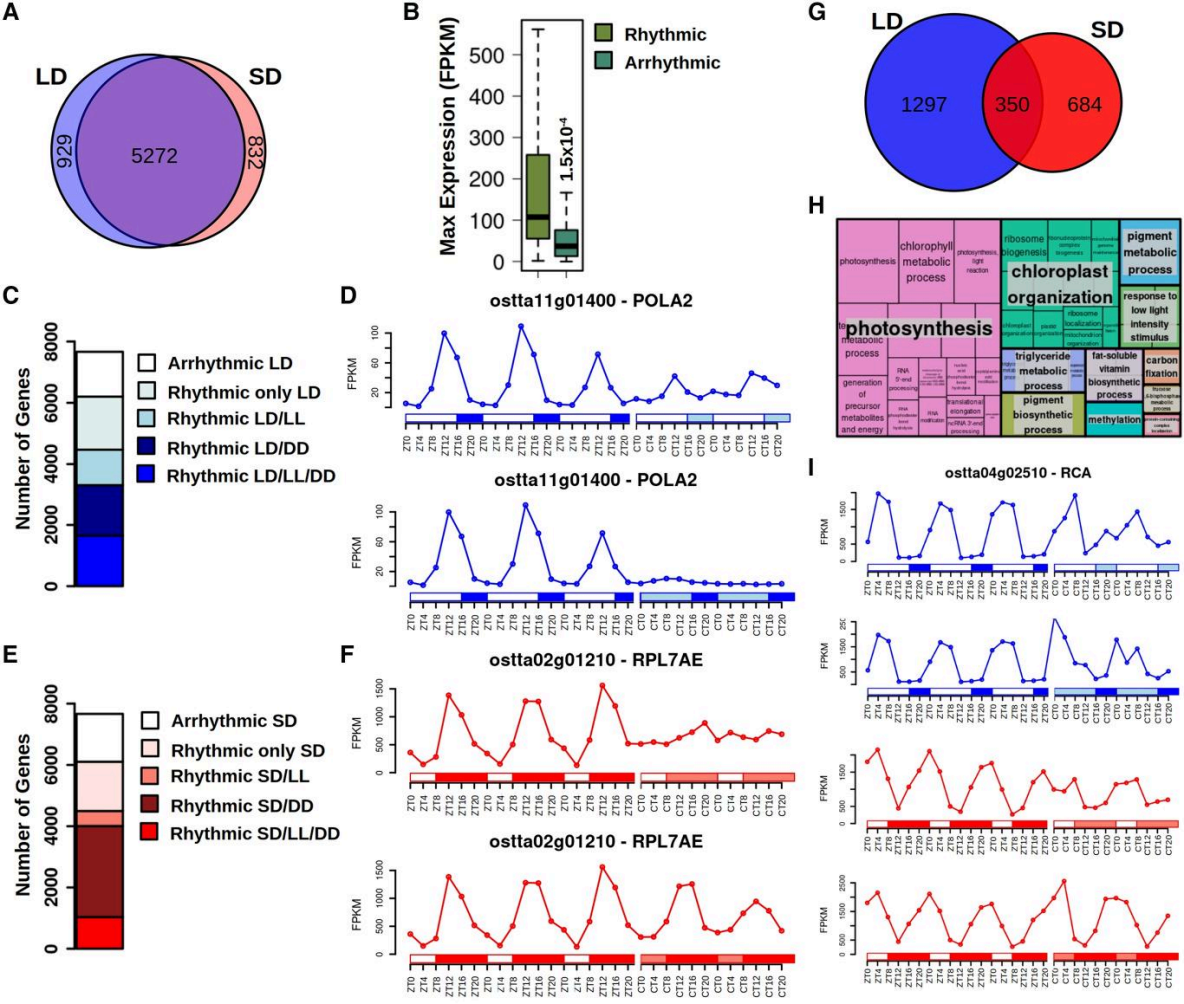
Transcriptome rhythmicity under alternating light/dark cycles and identification of circadian genes including free-running conditions. **A)** Venn diagram comparing rhythmic genes under LD (blue circle) and SD (light red circle). **B)** Boxplot representing the maximum expression level of rhythmic and arrhythmic genes. Medians are represented by central horizontal lines, upper and lower quartiles by boxes, minimum and maximum values by whisker ends. Gene expression levels are measured as FPKM. Significance was computed according to Mann–Whitney–Wilcoxon nonparametric test. **C)** Bar plot representing with blue colors different rhythmic gene sets under LD conditions. **D)** Gene expression profiles during 3 consecutive days under LD, 2 consecutive days under LL, and 2 consecutive days under DD of *DNA polymerase alpha subunit B* (*ostta11g01400*, *POLA2*). White rectangles represent photoperiods (light periods or days), blue-filled rectangles correspond to skotoperiods (dark periods or nights) under LD, light blue rectangles mark subjective nights or days under LL and DD, respectively after LD entrainment. ZTN, Zeitgeber time *N*, marks the time point *N* hours after dawn (lights on, ZT0). CTN, circadian time *N* denotes the time point *N* hours after subjective dawn. A discontinuity is shown on the time axis to indicate that samples were collected after 24 h acclimation to the corresponding free-running conditions. **E)** Bar plot representing with red colors different rhythmic gene sets under SD conditions. **F)** Gene expression profiles during 3 consecutive days under SD and 2 consecutive days under LL and DD of *Ribosomal protein L7Ae* (*ostta02g01210*, *RPL7AE*). White rectangles represent photoperiods, red-filled rectangles correspond to skotoperiods under SD, light red rectangles mark subjective nights or days under LL and DD, respectively, after SD entrainment. **G)** Venn diagram comparing circadian genes identified after LD entrainment (blue circle) and after SD entrainment (red circle). **H)** Treemap summarizing the biological processes significantly enriched over rhythmic genes under LD, SD, LL, and DD. Rectangle sizes represent significance levels. **I)** Gene expression profiles during 3 consecutive days under LD and SD; and 2 consecutive days under LL and DD of *RuBisCO Activase* (*ostta04g02510*) exemplifying rhythmic genes under LD, SD, LL, and DD involved in photosynthesis related processes.

In this study, photochemostats operated in a continuous regime (Supplementary Fig. S1A) were used simulating LD conditions (16 h light:8 h dark) or SD conditions (8 h light:16 h dark). The illuminating system simulated the progressive light intensity increase and decrease during solar daylight cycles with a maximum light irradiance of 1,500 μE m^−2^ s^−1^. Temperature and pH were maintained constant at 20 °C and 8. Samples were collected for 3 consecutive days every 4 h starting at ZT0, Zeitgeber time 0, the time point corresponding to the beginning of the photoperiod simulating dawn (Supplementary Fig. S1B). Robust global rhythmicity was detected in the transcriptomes under LD and SD conditions by using hierarchical clustering (HC) and principal component analysis (PCA) (Supplementary Fig. S1, C to E). A cyclic circular organization of the transcriptomes was revealed over diel cycles (Supplementary Fig. S1, D and E). Photoperiodic variations did not affect transcriptome rhythmicity, as the sets of rhythmic genes under LD and SD entrainment were near-identical, comprising ∼80% of the genome under either condition (Fig. 1A). Arrhythmic genes were either not expressed or lowly expressed. Indeed, rhythmic genes presented significant maximum expression levels 3-fold greater than arrhythmic genes (Fig. 1B). These genes are mainly involved in stress responses such as viral infection, and might not be expressed under these laboratory conditions.

In order to distinguish between circadian-regulated genes and those responding to the rhythmic environment, cultures were transferred to free-running conditions consisting of LL or DD. No samples were collected during the first day of constant conditions to allow culture acclimation, and samples were collected thereafter to generate transcriptomic data for 2 consecutive days starting at circadian time 0 (CT0), the time point corresponding to subjective dawn (Supplementary Fig. S1B). A clear reduction in rhythmicity was observed under constant conditions: ∼21% of the transcriptome was completely reliant on diel cycles to maintain rhythmicity since these genes lost their rhythms under both LL and DD (Fig. 1, C and E; Supplementary Table S1), independently from the entrainment regime. In contrast, the transcriptome proportion that maintained oscillations exclusively under LL or DD, but not both, was dependent on the previous entrainment. Whereas 15% kept cycling under LL after LD entrainment, only 6% were rhythmic after SD entrainment. The oscillating genes under LL, both after LD and SD entrainment, were found to be significantly involved in DNA replication and chromosome organization (Fig. 1D; Supplementary Fig. S2). The detrimental effect of DD over transcriptome rhythmicity was smaller than that of LL, with almost 22% maintaining oscillations after LD entrainment and 39% after SD entrainment. This observation is remarkable, as previous studies have found a complete lack of transcription under DD (O’-Neill et al. 2011). This can be attributed to the growth conditions in our experiments, which allow *Ostreococcus* cells to accumulate substantial amounts of starch to be used as energy source under DD to maintain oscillations (see section on starch content analysis). The biological processes RNA processing and ribosome biogenesis were found to be significantly enriched among the rhythmic genes under DD, both after LD and SD entrainment (Fig. 1F; Supplementary Fig. S3). Orthologues of the central components of the circadian clock, *ostta06g02340* (*CCA1*) and *ostta13g01820* (*TOC1*), exhibited rhythmicity under both LL and DD free-running conditions. CCA1 presented higher levels of expression under DD, in contrast to the halt in oscillations reported previously under different growth conditions (O’-Neill et al. 2011), while TOC1 showed higher expression levels under LL (Supplementary Fig. S4). Orthologues of the light receptors, *ostta15g0100* (*CRYPTOCHROME 1, CRY1*), *ostta01g06470* (*CRYPTOCHROME 3, CRY3*), and *ostta03g05620* (*UV RESISTANCE 2, UVR2*), were rhythmic under both LL and DD free-running conditions, with higher expression levels under LL than DD. Their rhythmic expression profiles were affected by photoperiodic variations except *CRY3*, which presented an identical pattern under both LD and SD but with an additional peak of expression at the beginning of the photoperiod under LD. This suggests a central position of these genes at the regulatory core of the circadian clock responding to photoperiodic variations in *Ostreococcus* (Supplementary Fig. S4).

Genes presenting rhythmicity under both LD and SD as well as maintaining their rhythmic expression profiles under both free-running conditions were considered in this study as those more directly regulated by the circadian clock in *Ostreococcus*. A clear dependence on the previous entrainment regime was observed, with 1,647 genes keeping rhythmicity under both free-running conditions after LD entrainment, versus 1,034 following SD entrainment. These 2 sets overlapped partially, identifying 350 genes corresponding to 4.6% of the *Ostreococcus* transcriptome (Fig. 1G; Supplementary Table S1). Functional enrichment analysis revealed that these genes are significantly involved in photosynthesis, chloroplast organization, and pigment metabolic processes (Fig. 1, H and I). The specific circadian genes detected after LD entrainment are significantly involved in ribosome biogenesis whereas those found after SD entrainment are primarily associated with cell cycle. This indicates a strong influence of the circadian clock over these processes. However, genes identified as circadian after LD entrainment lose rhythmicity under LL after SD entrainment, and the reverse for the specific circadian genes after SD entrainment that lose rhythmicity under DD after LD entrainment. This suggests a strong detrimental impact on rhythmicity of LL after SD entrainment with a short photoperiod, as well as constant darkness after LD entrainment with a short dark period (Supplementary Fig. S5).

**Figure 2.**
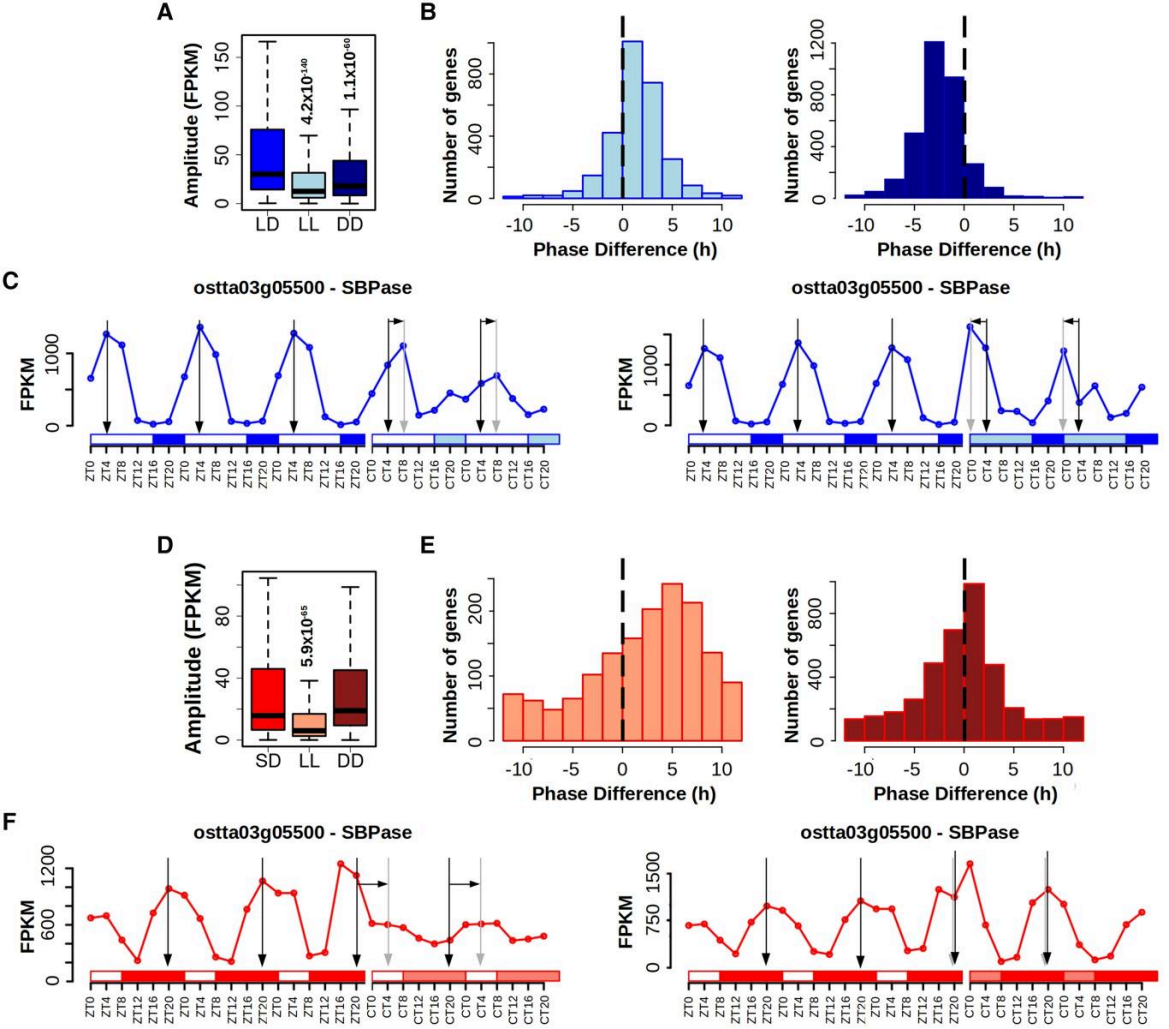
Free-running conditions effects over gene expression profiles. **A)** Boxplot representing rhythmic genes amplitude reached under LD conditions, when cultures were transferred to free-running conditions consisting of LL (light blue) and DD (dark blue) after LD entrainment. Medians are represented by central horizontal lines, upper and lower quartiles by boxes, minimum and maximum values by whisker ends. Gene expression levels are measured as FPKM. Significance was computed according to Mann–Whitney–Wilcoxon nonparametric test. **B)** Histograms showing the distribution of the number of genes exhibiting advanced and delayed phases when cultures are transferred from LD to LL (left) and DD (right). Vertical dashed lines mark no shift. **C)** Gene expression profiles during 3 consecutive days under LD and 2 consecutive days under LL and DD of *sedoheptulose-bisphosphatase* (*ostta03g05500*, *SBPase*). Vertical black arrows mark LD phases, vertical gray arrows mark LL and DD phases and horizontal black arrows represent delayed or advanced phases. White rectangles represent photoperiods (light periods or days), blue-filled rectangles correspond to skotoperiods (dark periods or nights) under LD, light blue rectangles mark subjective nights or days under LL and DD, respectively after LD entrainment. ZTN, Zeitgeber time *N*, marks the time point *N* hours after dawn (lights on, ZT0). CTN, circadian time *N*, denotes the time point *N* hours after subjective dawn. A discontinuity is shown on the time axis to indicate that samples were collected after 24 h acclimation to the corresponding free-running conditions. **D)** Boxplot representing rhythmic genes amplitude or maximum expression level reached under SD conditions, when cultures were transferred to LL and DD free-running conditions after SD entrainment. Significance was computed according to Mann–Whitney–Wilcoxon nonparametric test. **E)** Histograms showing the distribution of the number of genes exhibiting advanced and delayed phases when cultures are transferred from SD to free-running conditions consisting in LL (left) and DD (right). Vertical dashed lines mark no shift. **F)** Gene expression profiles during 3 consecutive days under LD and 2 consecutive days under LL and DD of *SBPase*. Vertical black arrows mark SD phases, vertical gray arrows mark LL and DD phases, and horizontal black arrows represent delayed phases.

In LD-entrained cultures, significant reductions in amplitude were detected under both LL and DD conditions for the LD/LL and LD/DD rhythmic genes, respectively (Fig. 2A). In SD-entrained cultures, amplitude reduction was only noted when transferred to LL for SD/LL rhythmic genes but no substantial change was found when transferred to DD for SD/DD rhythmic genes (Fig. 2D). Free-running conditions also affected the phase, or time point of maximum expression level, in rhythmicity patterns. Independently from the previous entrainment regime, phases were delayed when cultures were transferred to LL (Fig. 2, B and C, E and F, left). Whereas advanced phases (i.e. phases occurring earlier) were found when cultures were transferred to DD (Fig. 2, B and C, E and F, right), with this effect more pronounced for LD-entrained cultures and only slight for SD-entrained cultures. The phase delays in LL were more drastic for SD than LD-entrained cultures although the advances in DD were more evident for LD than SD-entrained cultures.

### Effects of photoperiodic variations over gene expression profiles

**Figure 3.**
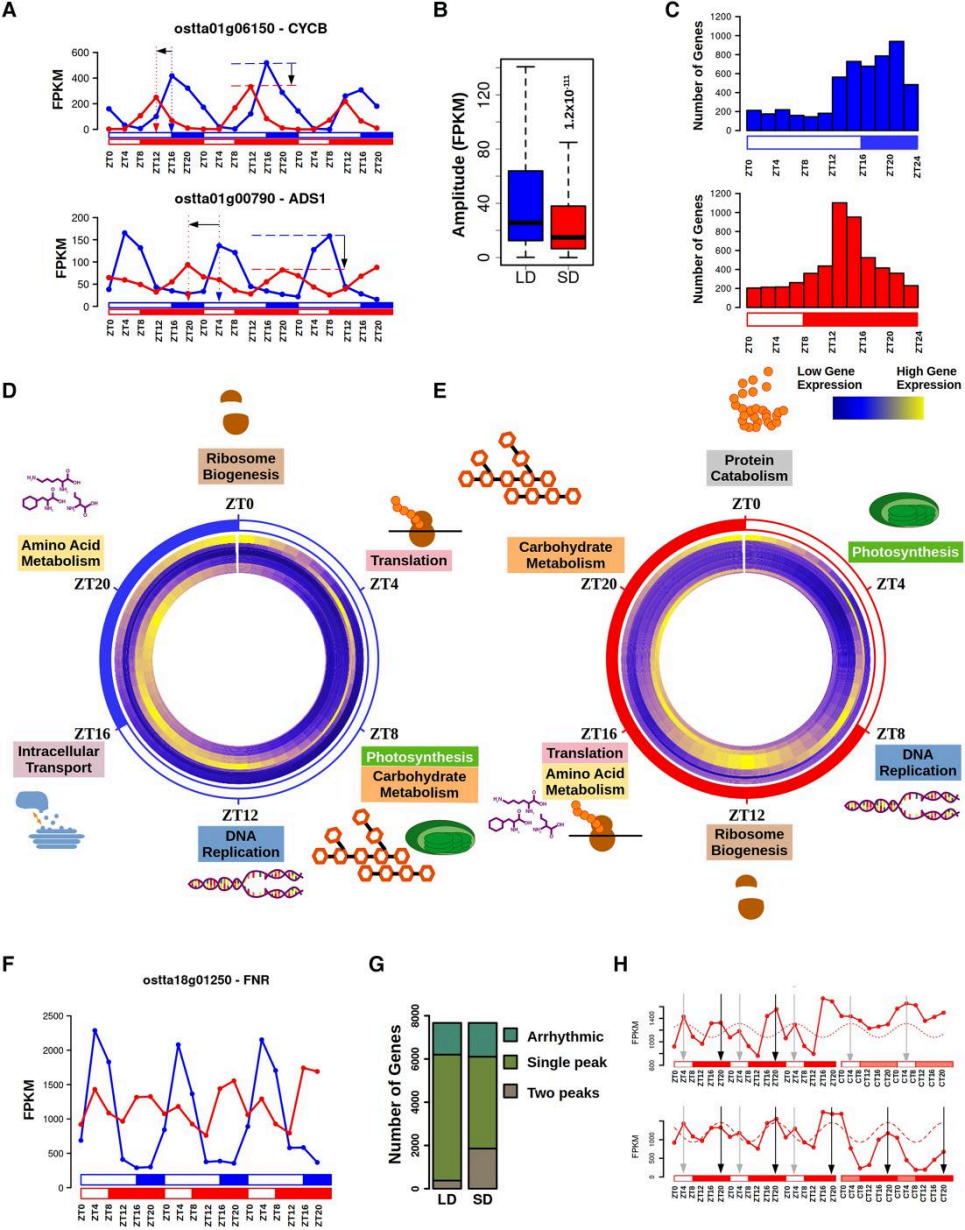
Photoperiodic effects over gene expression profiles. **A)** Gene expression profiles during 3 consecutive days under LD (blue) and SD (red) conditions of *CYCB* (*ostta01g06150*, top) and *delta-9 acyl-lipid desaturase 1* (*ostta01g00790*, *ADS1*, bottom). Blue and red vertical dotted arrows mark LD and SD phases. Horizontal black arrow represents advanced phase under SD when compared with LD. Blue and red horizontal dashed lines mark LD and SD amplitudes. Vertical black arrows represent the reductions in amplitude under SD with respect to LD. Light regimes are represented as described in Fig. 1. **B)** Boxplot representing rhythmic genes amplitude under LD in blue and SD in red. Medians are represented by central horizontal lines, upper and lower quartiles by boxes, minimum and maximum values by whisker ends. Gene expression levels are measured as FPKM. Significance was computed according to Mann–Whitney–Wilcoxon nonparametric test. **C)** Histograms showing the distribution of the number of genes with phase at specific time points during the day under LD (blue, top) and SD conditions (red, bottom). ZTN, Zeitgeber time *N*, marks the time point *N* hours after dawn (lights on). **D)** Circular heatmap representing the temporal organization of gene expression profiles under LD conditions. Dark blue stands for low expression, whereas yellow represents high expression. Genes are clustered depending on their phases. Genes with phase at ZT0 are located in the outer circle, while genes with phase at subsequent zeitgeber (ZT) are placed sequentially into inner circles. Biological processes enriched in the gene set with phase at each specific time point are depicted capturing the transcriptional program over diel cycles under LD condition. **E)** Similarly, circular heatmap representing the temporal organization of the transcriptional program under SD condition. **F)** Gene expression profiles during 3 consecutive days under LD (blue line) and SD (red line) of *Ferredoxin-NADP* *+* *reductase* (*ostta18g01250*, *FNR*). **G)** Bar plots representing in different green colors from top to bottom are the number of arrhythmic, single-peak rhythmic, and 2 peaks rhythmic genes under LD and SD conditions. **H)** Gene expression profiles during 3 consecutive days under SD and 2 consecutive days under free-running conditions consisting of LL or DD of *FNR* (*ostta18g01250*). This gene exemplifies how 2 peaks expression patterns under SD conditions could emerge as the combination of 2 distinct rhythmic profiles. One depending on the photoperiod (dotted line) with phase marked with a gray vertical arrow maintaining its rhythmicity only under LL (top). Another expression profile is apparent depending on the skotoperiod (dashed line) with phase marked with a black vertical arrow maintaining its rhythmicity only under DD (bottom).

Differences were identified in phase and amplitude of rhythmic gene expression patterns between LD and SD (Fig. 3A) with only 31 genes exhibiting the same expression profile under both photoperiods. A significant reduction in overall amplitude was observed in SD compared to LD-entrained cultures (Fig. 3B). Under both LD and SD, most genes peaked at night (Fig. 3C), indicating a “nocturnal transcriptome profile. Advanced phases were noted under SD with respect to LD conditions. Under LD entrainment, peak phases occur uniformly from the end of the day (ZT12) to the end of the night (ZT20), while under SD-entrainment peak phases predominantly occur during the first half of the night (ZT8 to ZT16; Fig. 3C).

Gene clusters were defined based on peak phases (Supplementary Table S2). Distinct diel timings of maximum gene expression levels for specific biological processes were identified by performing functional enrichment analysis over each gene cluster under LD (Fig. 3D; Supplementary Fig. S6) or SD conditions (Fig. 3E; Supplementary Fig. S7). Changes in the phase of biological processes under LD and SD conditions were detected. These differences in diel timings were produced by the previously mentioned advanced gene phases under SD with respect to LD. For example, genes involved in photosynthesis peak at ZT8 under LD and at ZT4 under SD, midday coinciding with the moment of maximal light irradiance in both conditions. Similarly, genes involved in DNA replication reached maximum expression level at late day (ZT12) under LD, and at early night (ZT8) under SD. Rearrangements in the order of peak phases for biological processes were also found. For instance, under LD conditions, genes involved in amino acid biosynthesis, ribosome biogenesis, and translation reached their highest level at ZT20, ZT0, and ZT4, respectively. However, in SD conditions, ribosome biogenesis genes were upregulated at ZT12, while both amino acid biosynthesis and translation-related genes reached maximum expression levels at ZT16.

**Figure 4.**
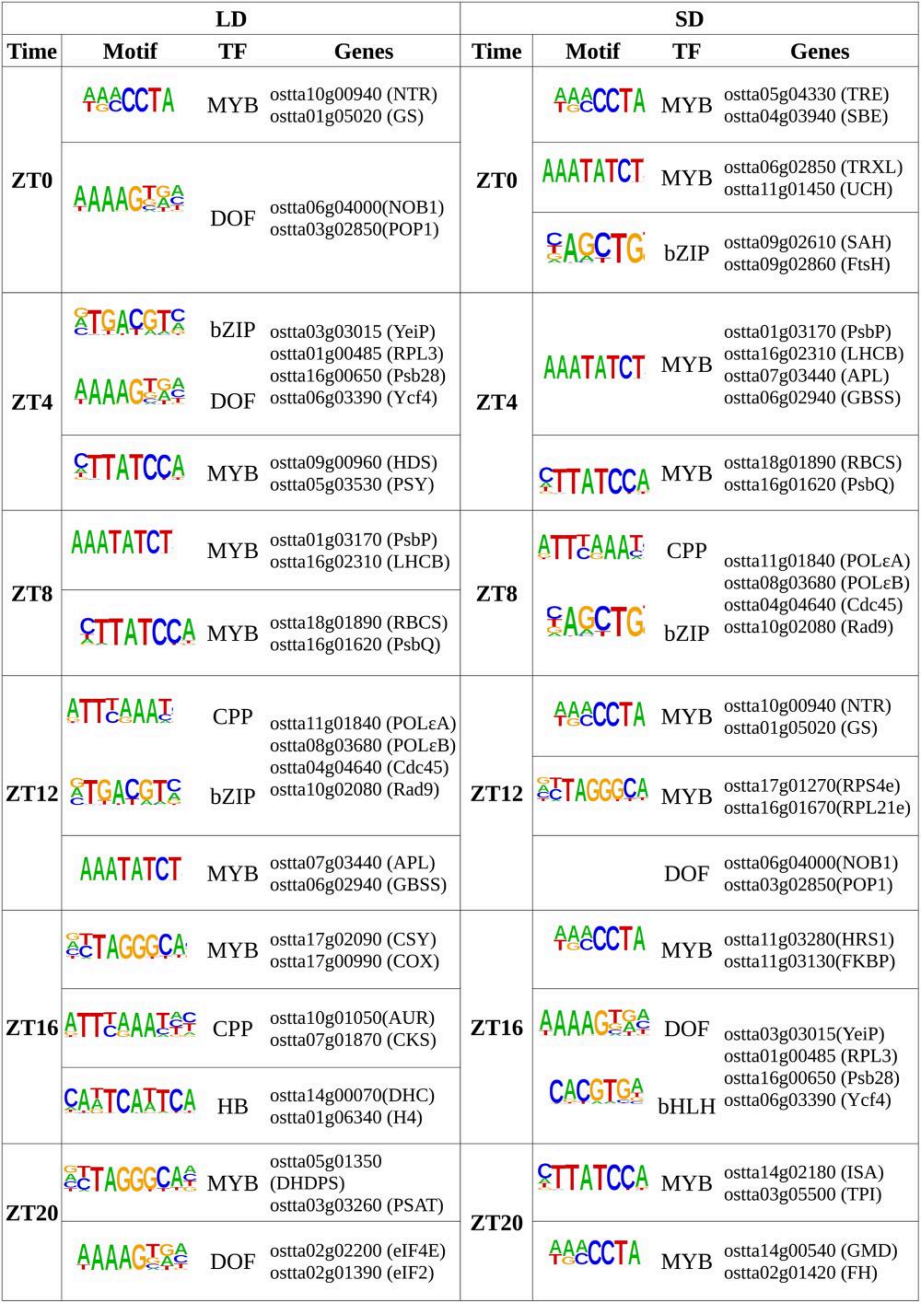
TFBS enriched in each time point gene cluster under LD and SD conditions.

To identify potential regulators of these distinct diel timings, transcription factor-binding sites (TFBS) enrichment analysis was performed over the promoters of genes in each time point cluster (Fig. 4). DNA motifs recognized by the families of plant transcription factors myeloblastosis (MYB), DNA-binding with one finger (DOF), basic leucine zipper (bZIP), cysteine-rich polycomb-like protein (CPP), and Homeobox were identified, suggesting a key role in regulating diel rhythmic gene expression patterns. Gene clusters corresponding to different time points under LD and SD conditions exhibited enrichment for the same DNA motifs, as these clusters share many genes in common due to the advanced gene phases under SD compared to LD. For instance, gene clusters corresponding to ZT12 under LD and ZT8 under SD showed enrichment for the DNA sequence recognized by the CPP transcription factor family. This family has been implicated in cell proliferation in *Arabidopsis* (Wang et al. 2018). Consistent with these findings the genes *ostta20g00800* and *ostta09g04220*, encoding CPP transcription factors, peaked at ZT12 under LD and ZT8 under SD conditions. Notably, the evening element (EE) motif, AAATATCT, was significantly identified in the promoters of the genes peaking at ZT12 under LD conditions and ZT0 and ZT4 under SD conditions. In *Arabidopsis*, the EE is associated with the binding site of CCA1 (Harmer et al. 2000). This transcription factor binds to its targets in the morning to repress their expression, resulting in maximum expression later in the evening (Kamioka et al. 2016). A similar gene expression pattern is observed in our transcriptomic data from *Ostreococcus*, suggesting a strong conservation at the central core of the circadian clock between *Arabidopsis* and *Ostreococcus*. Based on the presence of the EE in the gene promoters discussed above, CCA1 *Ostreococcus* orthologue, *ostta06g02340*, would be expected to bind in the morning to genes peaking at ZT12 under LD or late in the night to genes peaking at ZT0 or ZT4 under SD.

To capture the previously described changes in the phase and amplitude of rhythmic gene expression profiles resulting from photoperiodic variations, a co-sinusoidal dynamical model was developed. This model predicts the phase and amplitude of each rhythmic gene for a specific day of the year using linear interpolation between the phase and amplitude identified under LD or SD entrainment (Video 1). Previously published microarray data, generated under neutral day conditions (ND) (12 h light:12 h dark) (Monnier et al. 2010) were used to evaluate its predictive power. The phases of approximately two-thirds (63%) of the rhythmic genes were successfully predicted with ±4 h error. The limited predictive power of our model suggests that while two-thirds of the rhythmic genes respond to changes in photoperiod by gradually adjusting their phases, the remaining one-third exhibit a more complex response to photoperiod changes.

**Video 1.** Changes in the phase and amplitude of rhythmic gene expression profiles as a consequence of seasonal photoperiod lengthening/shortening. Animation illustrating how seasonal photoperiod lengthening may result in a gradually delayed phase coupled to a gradual increase in amplitude. Accordingly, seasonal photoperiod shortening may result in a gradually advanced phase coupled to a gradual decrease in amplitude. White rectangles represent photoperiods (light periods or days) and color-filled rectangles correspond to skotoperiods (dark periods or nights). Transitions from red to blue colors and vice versa represent seasonal changes in photoperiods and skotoperiods.

Another difference observed between LD and SD conditions was related to the occurrence of gene expression profiles with 2 daily peaks (Fig. 3F). Although gene profiles with 2 peaks were detected in both LD and SD cultures (Supplementary Table S3), a drastic increase in the number of genes with bimodal rhythmicity was observed under SD condition (1,855 versus 376 genes; Fig. 3G). Bimodal rhythmicity was not maintained under free-running conditions in which distinct single-peak profiles were apparent under LL and DD (Fig. 3H). Nonlinear squares (Baty et al. 2015) were applied to decompose the observed bimodal gene profile in SD-entrained cultures into 2 different single-peak profiles, one peaking during the photoperiod and the other during the skotoperiod. Under LL free-running condition, only the photoperiod-peaking profile was maintained, whereas under DD condition only the skotoperiod-peaking profile was present. For genes with bimodal rhythmicity, a dynamical model was developed combining 2 distinct co-sinusoidal profiles to capture changes in the phase and amplitude, resulting from changes in photoperiod length. Under LD conditions, these profiles overlap in time producing a single-peak profile, whereas under SD condition they become out of phase, resulting in a bimodal profile (Video 2). To test this model, predictions were run to assess rhythmicity profiles under ND conditions. The model accurately predicted the emergence of bimodal rhythmic profiles, as identified in microarray data from ND conditions, validating our model (Supplementary Fig. S8).

**Video 2.** Emergence of 2 peaks gene expression patterns as a consequence of seasonal photoperiod or day shortening (skotoperiod or night lengthening). Animation illustrating how seasonal day or photoperiod shortening (night or skotoperiod lengthening) may result in the emergence of 2 peaks gene expression patterns as a consequence of the 2 constituent gene expression profiles becoming out of phase. The observed gene expression pattern represented by a continuous thick line can result from the combination of 2 distinct expression profiles represented by thin dotted and dashed lines. One of these expression profiles (dotted line) may not respond to seasonal changes in photoperiod/skotoperiod, whereas the other one (dashed line) may experience phase shifts and amplitude changes. Under LD conditions (alternating 16 h light/8 h dark) the phases or maximum expression-level time point of both expression profiles may coincide resulting in a single-peak expression pattern. Whereas under SD conditions (alternating 8 h light/16 h dark) the phases may be reached at different time points producing a 2 peaks expression pattern. Transitions from red to blue colors and vice versa represent seasonal changes in photoperiods and skotoperiods resulting in gradual transitions from 2 peaks to a single-peak expression pattern.

### Proteome rhythmicity under photoperiodic variations and integration with transcriptomic rhythmic patterns

As proteins are the primary actors of biological processes, proteomic analyses provide a direct assessment of the functional consequences of environmental rhythms (Kay et al. 2021). In this study, a total of 3,672 proteins were successfully quantified under LD or SD conditions (Supplementary Fig. S9A), accounting for ∼48% of the predicted *Ostreococcus* proteome (Supplementary Table S4). The predicted subcellular location of these proteins indicates coverage of all subcellular locations (Supplementary Fig. S9B).

**Figure 5.**
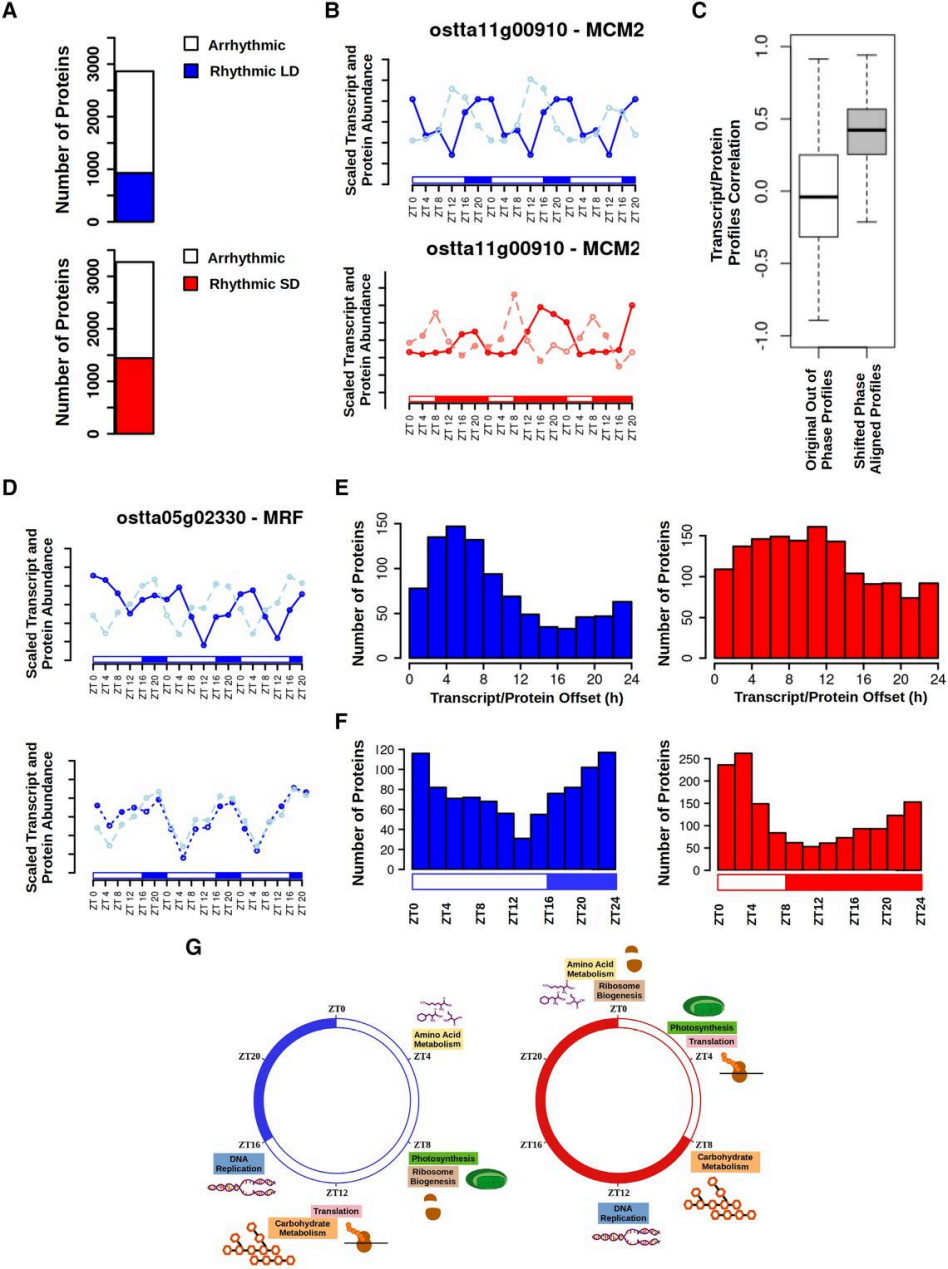
Proteome rhythmicity under alternating light/dark cycles and temporal offsets with respect to the corresponding transcriptome. **A)** Bar plots representing the number of identified proteins under LD (top) and under SD (bottom) conditions. The number of rhythmic proteins under LD condition is represented in blue and under SD condition in red. Arrhythmic proteins are represented in white. **B)** Scaled transcript (light line) and protein (dark line) abundance profiles during 3 consecutive days under LD (top, blue) and SD (bottom, red) conditions for *Minichromosome Maintenance 2* (*ostta11g00910*, *MCM2*). White rectangles represent photoperiods (light periods or days), blue- and red-filled rectangles correspond to skotoperiods (dark periods or nights) under LD and SD, respectively. ZTN, Zeitgeber time *N*, marks the time point *N* hours after dawn (lights on, ZT0). **C)** Boxplots representing the global distribution of the correlations between transcript and protein abundance profiles (white box) and shifted aligned profiles with coincident phases (gray). Medians are represented by central horizontal lines, upper and lower quartiles by boxes, minimum and maximum values by whisker ends. **D)** Top, protein abundance (continuous blue line) and gene expression (dashed light blue line) profiles during 3 consecutive days under LD condition for *MA3 domain-containing translation regulatory factor* (*ostta05g02330*). Bottom, phase-aligned protein abundance (dotted blue line) and gene expression (dashed light blue line) profiles. **E)** Histograms showing the distribution of the number of proteins with specific offsets between transcript and protein abundance phases (time points of maximum transcript/protein abundance) under LD condition (left, blue) and SD condition (right, red). **F)** Histograms showing the distribution of the number of proteins with phase or maximum abundance at specific time points under LD condition (left, blue) and SD condition (right, red). **G)** Temporal organization of biological processes based on the time point when proteins involved in the corresponding process reach its maximum abundance under LD condition, left in blue, and SD condition, right in red.

The estimated number of rhythmic proteins was notably much lower than that of rhythmic genes. Specifically, 928 and 1,442 rhythmic proteins were identified under LD and SD conditions, respectively (Fig. 5A; Supplementary Fig. S9, C and D and Table S5).

Globally, temporal phase offsets were detected between rhythmic protein abundance profiles and the corresponding transcript profiles with gene expression preceding protein abundance several hours (Fig. 5B). A direct relationship between transcript and protein abundance becomes clear upon calculating correlation following phase alignment of transcript and protein profiles (Fig. 5, C and D). This suggests that protein and transcript abundance relate linearly but with a clear temporal separation. In LD conditions, the distribution of transcript/protein phase temporal offsets centered around 5 to 6 h, consistent with protein abundance following transcript abundance. However, under SD, offsets followed a more uniform distribution and were significantly longer than under LD (Fig. 5E), indicating that many proteins do not follow on predictably from transcripts. Under both conditions, a “diurnal character was observed for the *Ostreococcus* proteome with most proteins reaching maximum abundance during the day (Fig. 5F). Notably, this is in contrast with the “nocturnal character observed for the transcriptome (Fig. 3B). Transcript/protein phase offsets result in different diel timings of biological processes based on the time point when the corresponding proteins reach their maximum abundances (Fig. 5G) compared to the diel timings of maximum gene expression levels (Fig. 3, D and E).

**Figure 6.**
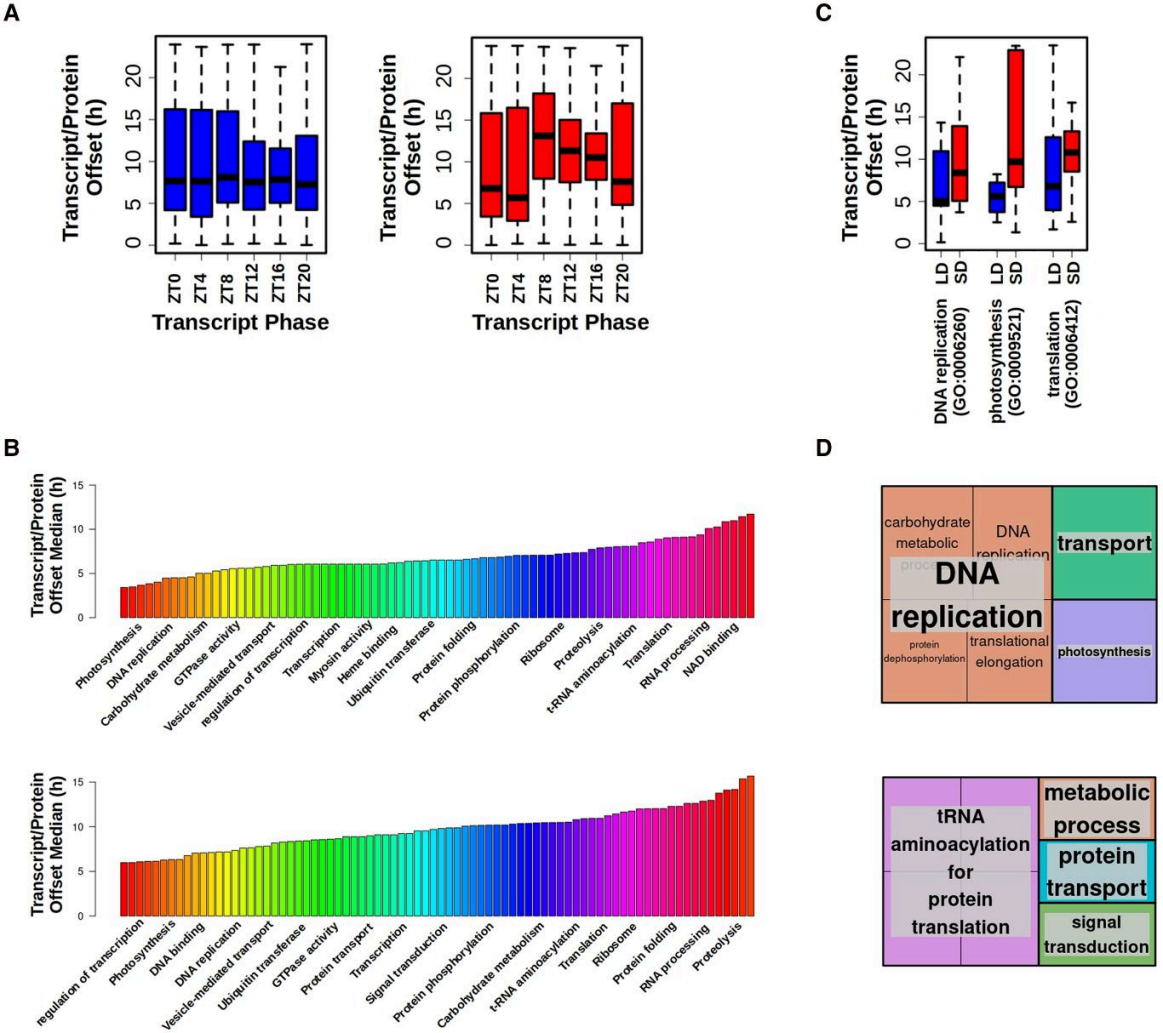
Analysis of the temporal offsets between transcript and protein abundance profiles. **A)** Boxplots representing transcript/protein offsets under LD (left, blue) and SD (right, red) conditions for different gene sets with specific phases. Medians are represented by central horizontal lines, upper and lower quartiles by boxes, minimum and maximum values by whisker ends. **B)** Median transcript/protein offset for gene sets annotated with the same GO term under LD conditions (top) and SD conditions (bottom). Different biological processes identified by specific GO terms present distinct short or long transcript/protein offsets. **C)** Boxplot illustrating how genes involved in different biological processes according to their GO annotation present distinct transcript/protein offsets that are longer under SD (red) than LD (blue) conditions. Medians are represented by central horizontal lines, upper and lower quartiles by boxes, minimum and maximum values by whisker ends. DNA replication (GO: 0006260), photosynthesis (GO: 0009521), and translation (GO: 0006412) are chosen as examples exhibiting short and long transcript/protein offsets. **D)** Treemaps summarizing the biological processes with shortest transcript/protein offsets (top) and with longest transcript/protein offsets (bottom). Semantically similar biological processes are grouped into the same colored rectangles. The most representative biological processes are shown for each rectangle.

Transcript/protein phase offsets did not correlate to any biochemical properties computed from protein sequences, such as amino acid composition, charge, or hydrophobicity (Supplementary Fig. S10). Similarly, transcript/protein phase offset did not correlate with transcript phase under LD condition. However, under SD condition transcript/protein offsets were significantly longer for transcripts peaking during the skotoperiod, ZT8, ZT12, ZT16, and ZT20, when compared to those genes with transcript phases during the photoperiod, ZT0 and ZT4 (Fig. 6A). This may be due to translation occurring preferentially during the light period as suggested in our analysis (Fig. 5G) and in previous studies for *Arabidopsis* (Seaton et al. 2018). Notably, distinct short or long transcript/protein phase offsets were observed between genes involved in different biological processes, as identified by gene ontology (GO) analyses (Fig. 6B). Specifically, biological processes associated with short offsets were DNA replication and photosynthesis. Translation on the other hand was one of the most representative biological processes exhibiting long transcript/protein offsets (Fig. 6, C and D).

### Proteome and transcriptome rhythmicity combine to orchestrate physiological rhythms

Rhythmic patterns have been described in microalgae, including *Ostreococcus*, for physiological processes such as cell division, photosynthesis, and metabolism (Mittag 2001; Moulager et al. 2007; Sorokina et al. 2011; Shi et al. 2022). In this study, transcriptomic and proteomic data have been integrated with measurements of cell-cycle progression and metabolism to elucidate the temporal orchestration that underlies dynamic physiology.

#### Cell-cycle progression

**Figure 7.**
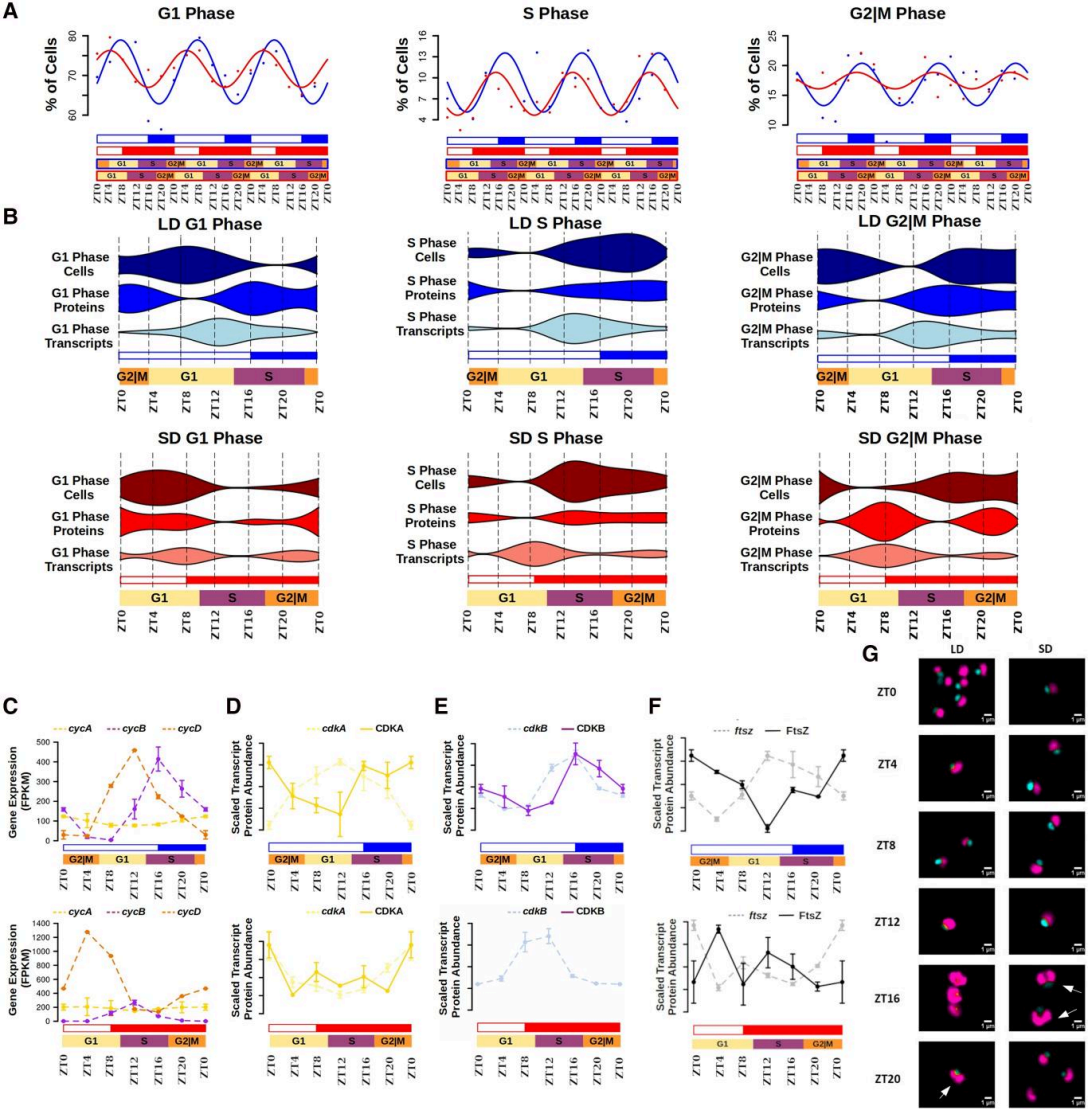
Cell cycle progression rhythmicity and integration with transcriptome and proteome dynamics under long and SD conditions. **A)** Dots correspond to flow cytometry measurements and lines represent the co-sinusoidal model best fitting these data. LD (in blue) and SD (in red) conditions are represented. White rectangles represent photoperiods (light periods or days), blue- and red-filled rectangles correspond to skotoperiods (dark periods or nights) under LD and SD, respectively. ZTN, Zeitgeber time *N*, marks the time point *N* hours after dawn (lights on, ZT0). Cell cycle phases are framed by blue and red rectangles for LD and SD, respectively. Gap 1 (G1) phase is represented by yellow rectangles, DNA synthesis (S) phase by purple rectangles, and Gap2 and Mitotic (G2|M) phase by orange rectangles. **B)** Violin plots integrating, from top to bottom, the percentage of cells in each cell cycle phase with protein and transcript abundance profiles under LD (top, blue) and SD (bottom, red) conditions. Violin widths represent the corresponding average percentage of cells, transcript and protein abundance. **C)** *Cyclin A* (*cycA*, yellow); *B* (*cycB*, purple); and *D* (*cycD*, orange) mean gene expression of 3 consecutive days measured as FPKM under LD (top, blue) and SD (bottom, red) conditions. For each time point standard errors are represented as vertical lines. **D to F)** Mean scaled transcript (dashed light) and protein (continuous line) abundance profiles of 3 consecutive days for CDKA, CDKB, and filamentous temperature-sensitive Z (FtsZ) under LD (top, blue) and SD (bottom, red) conditions. For each time point Se are represented as vertical lines. **G)** Confocal microscopy images of *Ostreococcus* cells at different time points (rows) under LD (left column) and SD (right column) conditions. Cyan marks nuclear DNA, while purple represents chloroplast fluorescence. White arrows point to cells with 2 chloroplasts.

Flow cytometry was used to assess the cell cycle based on total DNA content in *Ostreococcus* observing rhythmicity in all cell-cycle phases under both LD and SD conditions (Fig. 7A; Supplementary Table S6). A reduction of ∼24% was observed in the number of cells entering S phase under SD compared to LD. In SD conditions, cells remained in either G1 or G2 phase. Furthermore, significant delays of ∼4 h were observed in the cell-cycle phases under LD compared to SD (Fig. 7A). These observations are consistent with the shifts in the time points of maximum transcript and protein abundance found under short versus long photoperiods. For key genes associated with different cell-cycle phases (Robbens et al. 2005), the corresponding phase of transcript and protein abundance under LD and SD conditions were assessed (Supplementary Table S6). Transcript and protein abundance profiles were compared to the cell-cycle phases using violin plots. Clear temporal offsets were observed with gene expression preceding protein abundance levels and shorter temporal offsets between protein abundance and the cell percentage in each cell-cycle phase (Fig. 7B). These gene expression and protein abundance profiles refer to the genes associated with different cell-cycle phases in Supplementary Table S6.

Cyclins and cyclin-dependent kinases (CDKs) are essential components of the molecular machinery governing cell-cycle progression (Corellou et al. 2005). Their gene expression and protein abundance profiles were examined and integrated with the temporal cell-cycle phase progression under LD and SD (Fig. 7, C to E). *CyclinD* (*CYCD, ostta18g01570*) expression increased from the beginning to the middle of the G1 phase (ZT4—ZT9 under LD; ZT0—ZT5 under SD), consistent with a role in promoting cell-cycle entry and progression to the S phase. During the second half of the G1 phase and the first half of the S phase (ZT9—ZT18 under LD and ZT5—ZT14 under SD), *CyCD* expression decreased, while *Cyclin B* (*CYCB, ostta01g06150*) expression increased, consistent with its role in inducing progression toward G2|M phase. A similar pattern was observed for both transcript and protein abundance of *cyclin-dependent kinase B* (*CDKB, ostta15g00670*) under LD and SD. *Cyclin A* (*CycA, ostta02g00150*) was lowly expressed, peaking during G2|M phase (ZT0 under LD and ZT20 under SD). *Cyclin-dependent kinase A* (*CDKA, ostta04g00110*) transcript abundance peaked at the end of the G1 phase (ZT12) under LD conditions, preceding the protein abundance peak at ZT20 during the S phase. However, in SD conditions, *CDKA* gene expression and protein abundance both peaked at ZT0 at the end of the G2|M phase with no apparent temporal offset. Genes involved in chloroplast division, such as *Filamentous Temperature-Sensitive Z* (*FtsZ*, *ostta07g01610*), peaked at the end of the G1 phase (ZT12 in LD and ZT8 in SD), preceding the protein peaks reached during the transition S/G2|M phase (ZT0 in LD and ZT16 in SD; Fig. 7F). Confocal microscopy images validated these findings by identifying cells with 2 chloroplasts as a result of recent divisions at ZT20 under LD and ZT16 under SD (Fig. 7G).

#### Photosynthesis and starch biosynthesis

**Figure 8.**
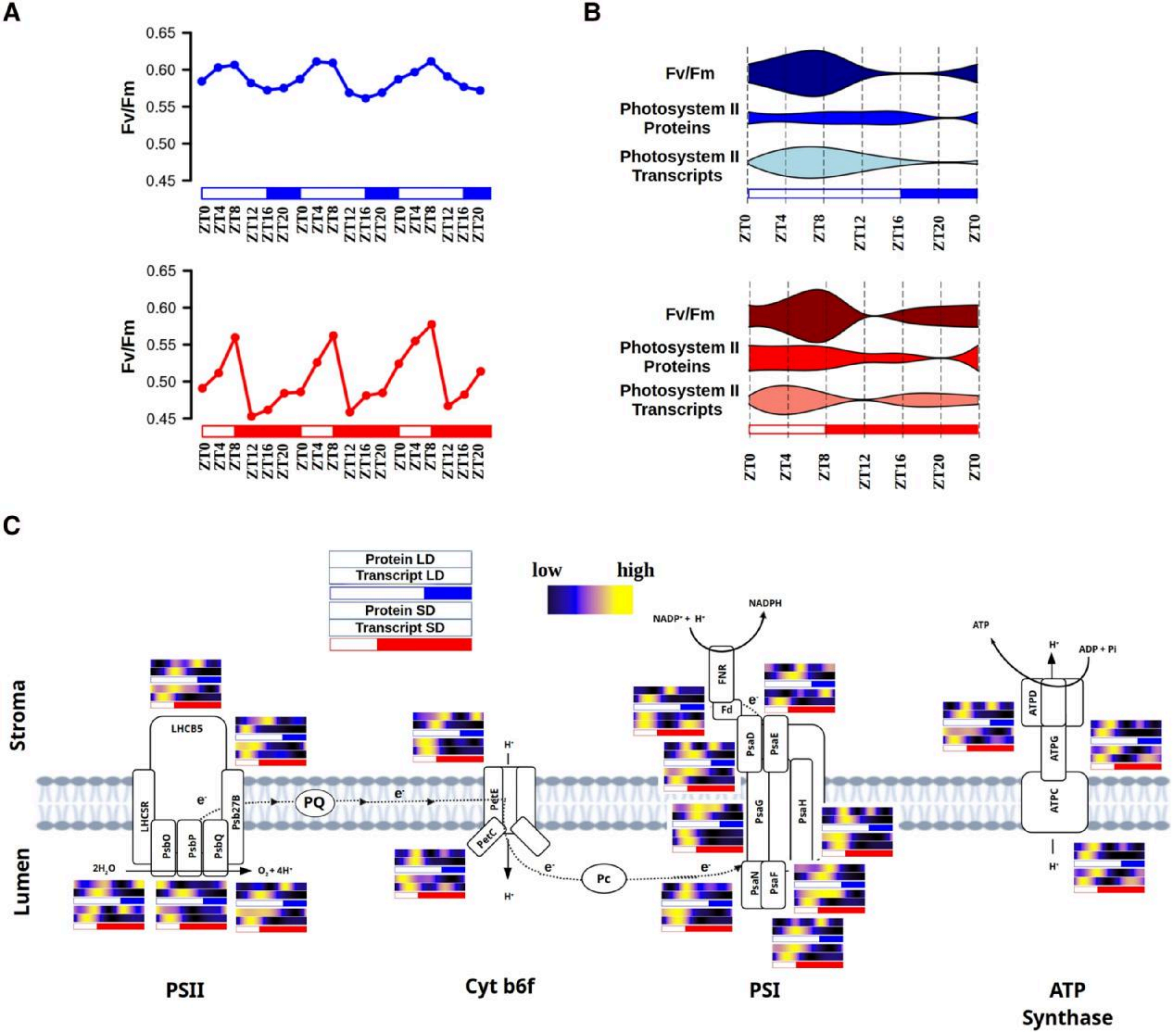
Integration of photosynthetic efficiency profiles with transcriptome and proteome dynamics under LD and SD conditions. **A)** PSII maximum quantum efficiency, Fv/Fm, during 3 consecutive days under LD (top in blue) and SD (bottom in red) conditions. White rectangles represent photoperiods (light periods or days), blue- and red-filled rectangles correspond to skotoperiods (dark periods or nights) under LD and SD. **B)** Violin plots integrating, from top to bottom, Fv/Fm values with PSII protein and transcript abundance profiles under LD (top, blue) and SD (bottom, red) conditions. Violin widths represent the corresponding average Fv/Fm values, PSII protein and transcript abundance. **C)** Graphical representation of photosynthesis processes involving PSII, cytochrome b6f (Cyt b6f), PSI, and ATP Synthase. For each enzyme its corresponding protein and transcript abundances under LD, top in blue, and SD conditions, bottom in red, are represented using heatmaps. Black represents low, blue medium, and yellow high abundances. White rectangles represent photoperiods, blue- and red-filled rectangles correspond to skotoperiods under LD and SD, respectively.

The photosynthetic machinery must dynamically adapt to seasonal photoperiodic variations in diel cycles to optimize energy conversion (Masojídek et al. 2021). To assess the overall integrity and efficiency of photosystem II (PSII), the maximum quantum efficiency, Fv/Fm, was measured throughout diel cycles under LD and SD conditions (Fig. 8A). Fv/Fm measurements exhibited rhythmicity under both LD and SD conditions, peaking at ZT8 in both the cases, albeit slightly lower (7%) under SD compared to LD. In contrast, Fv/Fm reached its minimum at the end of the photoperiod (ZT16) under LD but at early night (ZT12) under SD, with a 19% reduction. Violin plots were used to compare Fv/Fm measurements with transcript and protein abundance profiles of genes associated with PSII (Fig. 8B). Under LD conditions, no temporal offsets were observed between the time points of maximum transcript and protein abundances and the highest Fv/Fm values. However, under SD condition, short temporal offsets were identified between profiles. Specifically, the early increase in gene expression at dusk, observed in, for example, genes encoding components of the oxygen evolving complex *PSII subunits O*, *P*, and *Q* (*PsbO*, *ostta14g00150*; *PsbP*, *ostta14g02630*; and *PsbQ*, *ostta16g01620*) precede a concomitant increase in protein abundance and Fv/Fm values during the second half of the night (Fig. 8C; Supplementary Fig. S11). Similar expression patterns resulting in profiles with 2 peaks under SD conditions were found in genes such as *Protein Electron Transfer C* (*PetC*, *ostta01g06610*), *Ferredoxin* (*Fd*, *ostta17g00310*), and *ATPase delta subunit* (*ATPD*, *ostta07g01350*) coding for key components of Cytochrome b6f, photosystem I (PSI), and ATP synthase, respectively (Fig. 8C; Supplementary Fig. S11). These genes constitute examples of the emergence of 2 peaks under SD conditions, one induced by the photoperiod maintained only under LL, and the other one by the skotoperiod, maintained only under DD. Moreover, these genes maintained rhythmicity under all conditions except LL after SD entrainment indicating a strong control of the circadian clock over them and a detrimental effect of LL over their rhythmicity after an entrainment with short photoperiod.

**Figure 9.**
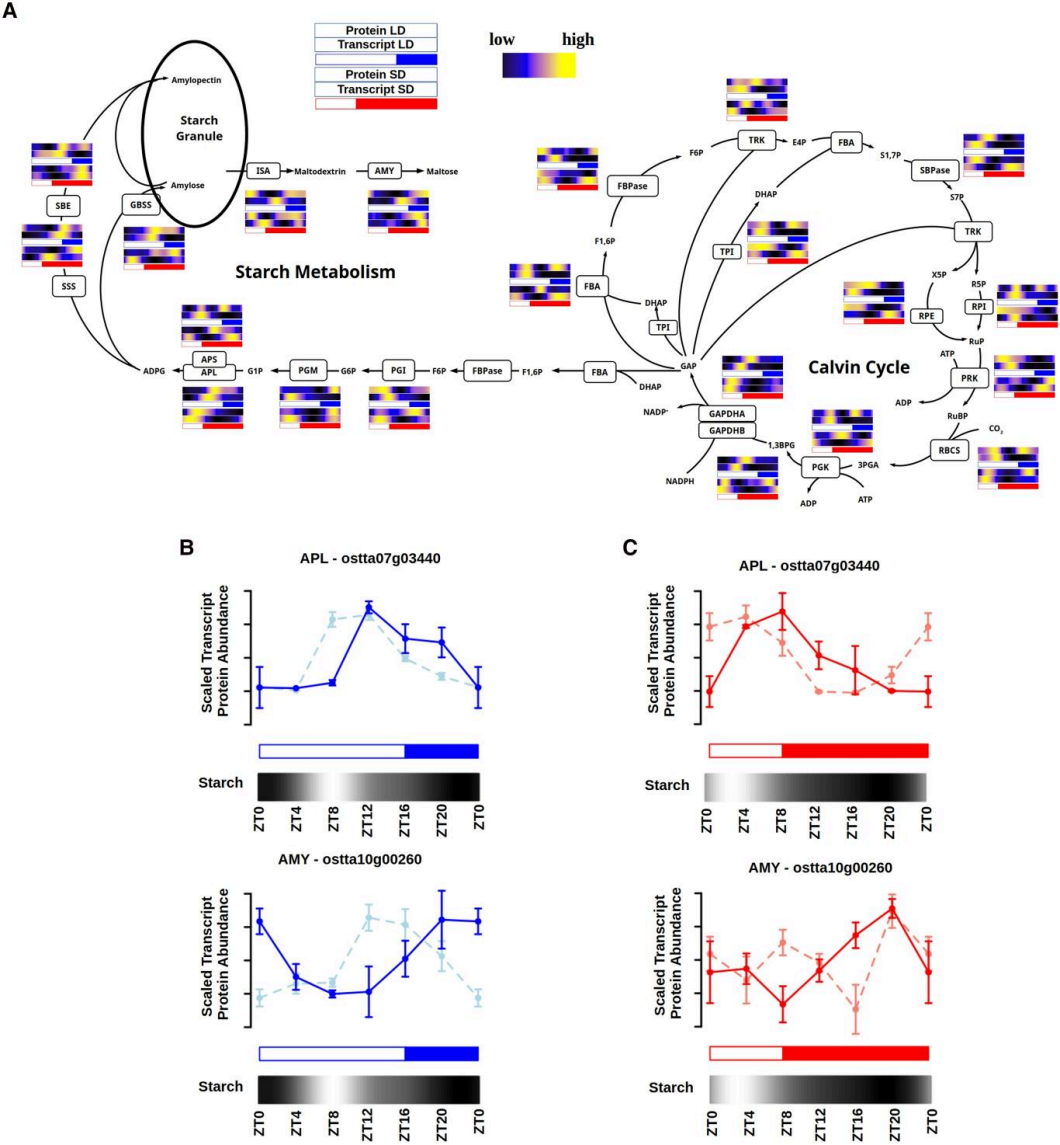
Integration of starch accumulation profiles with transcript and protein abundances for enzymes involved in the Calvin cycle and starch metabolism. **A)** Graphical representation of the Calvin cycle leading to starch metabolism. For each enzyme, its corresponding protein and transcript abundances under LD (top in blue) and SD (bottom in red) conditions are represented using heatmaps. Black represents low, blue medium, and yellow high abundances. White rectangles represent photoperiods (light periods or days), blue- and red-filled rectangles correspond to skotoperiods (dark periods or nights) under LD and SD, respectively. **B)** Mean scaled transcript (light blue line) and protein (continuous blue line) abundance profiles of 3 consecutive days for ADP-glucose pyrophosphorylase large subunit (APL) top and for AMY bottom under LD conditions. For each time point Se are represented as vertical lines. Starch content is represented using a heatmap where the lowest amount is represented in black and the highest amount in white. **C)** Mean scaled transcript (light red line) and protein (continuous red line) abundance profiles of 3 consecutive days for APL top and AMY bottom under SD conditions. Vertical lines represent Se. Starch content is represented using a heatmap, where the lowest amount is represented with black and the highest amount with white.

Genes coding for key components of the Calvin cycle such as *Glyceraldehyde-3-phosphate dehydrogenase A* (*GAPDHA*, *ostta01g01560*) and *Fructose-1,6-bisphosphate aldolase* (*FBA*, *ostta01g03040*) are examples of genes exhibiting rhythmicity under all conditions, LD, SD, LL, and DD, suggesting a direct control by the circadian clock. In all these examples no or short temporal offsets between transcript peaks (ZT8 under LD and ZT4 under SD) and protein peaks were found (Fig. 9A; Supplementary Fig. S11). Glucose, a product of carbon fixation, can be stored long-term as starch. To further investigate the orchestration between transcript and protein abundances with physiological measurements, starch content was assessed throughout diel cycles under LD and SD conditions. Starch content profiles exhibited rhythmicity under both LD conditions, reaching a peak of 41.2% of dry weight (DW) at ZT8; and under SD conditions, peaking at ZT4 with a value of 33.4% of DW (Supplementary Table S7). A clear temporal sequence in gene activation preceding protein accumulation was observed, with temporal offsets of ∼4 h for the enzymes involved in starch metabolism (Fig. 9A; Supplementary Fig. S11). Specifically, the first committed step in starch metabolism, the conversion of glucose-1-phosphate into ADP-glucose, is catalyzed by ADP-glucose pyrophosphorylase, with its small and large subunit genes (*APS*, *ostta07g03070* and *APL*, *ostta07g03440*) reaching maximum gene expression levels at ZT8 and ZT4 under LD and SD conditions, respectively. The activation of these genes preceded the peaks of the corresponding proteins by ∼4 h. Subsequent steps involve the synthesis of amylose by granule bound starch synthase (*GBSS*, *ostta06g02940*) and amylopectin by starch branching enzyme (*SBE*, *ostta03g00870*). The corresponding transcripts peaked at ZT12 under LD and presented 2 peaks at ZT20 and ZT4 under SD, while the encoded proteins reached their maximum abundances at ZT16 both under LD and SD conditions. The elongation of both amylose and amylopectin is performed by soluble starch synthase (*SSS*, *ostta16g02480*), which peaked shortly preceding protein abundance, at ZT16 (LD) and ZT12 (SD). Starch degradation involves the enzymes *debranching enzyme* or *isoamylase* (*ISA*, *ostta02g05060*) and *amylase* (*AMY*, *ostta10g00260*), which produce maltodextrin and maltose. The genes encoding these proteins reached maximum expression at ZT16 under LD and ZT8 under SD, preceding the accumulation of the corresponding proteins that reached their maximum by night (Fig. 9A). By integrating these gene expression and protein abundance patterns with starch content profiles over LD and SD diel cycles, it was patent that starch accumulation is the result of a balance between synthesis and degradation. Under both LD and SD conditions, starch content reached its maximum at midday (ZT8 under LD and ZT4 under SD), despite the enzymes involved in starch biosynthesis and their corresponding genes peaking several hours later, toward the end of the day. The halt in starch content increase and its subsequent decrease can be attributed to the activation of the genes encoding enzymes involved in starch catabolism, and the increased abundance of the corresponding proteins during the second half of the day, both under LD and SD conditions (Fig. 9, B and C).

Previous studies on *Ostreococcus* have reported that the clock components stop oscillating under DD (O’-Neill et al. 2011). However, in our cultures, key components such as CCA1 transcripts maintain oscillations under DD and LL after both LD and SD entrainment. The rhythmic gene expression of CCA1 under DD in our cultures was validated using RT-qPCR (Supplementary Fig. S12). Our analysis of the dynamics of starch content can explain this apparent discrepancy, which can be attributed to the significant disparities in growth conditions between our experiments and those previously published. Our cultures are maintained in continuous mode, with a steady supply of fresh high nitrate medium, illuminated simulating solar daylight cycles with a maximum light irradiance of 1,500 μE m^−2 ^s^−1^ and CO_2_ injection to regulate pH levels. In contrast, in previously published studies, cells are grown in batch mode without aeration, with low nitrate medium and blue light irradiance maintained constant at 11.5 μE m^−2 ^s^−1^ during the photoperiod. Under our growth conditions, *Ostreococcus* cells accumulate substantial amounts of starch, comprising 30% of DW (Supplementary Table S7). These starch reserves are gradually consumed under DD maintaining high levels after 48 h, indicating continued metabolic activity in *Ostreococcus* cells (Supplementary Fig. S12). The energy derived from starch catabolism likely prevents nutritional stress, thereby enabling the clock to function under DD. Conversely, in batch cultures without aeration and with low light irradiance, *Ostreococcus* cells synthesize starch constituting around 5% of DW during the light period, which is nearly entirely consumed during the subsequent 12 h night (Supplementary Fig. S12). This suggests that under such conditions, *Ostreococcus* cells accumulate sufficient starch reserves for one single night only, similar to Arabidopsis (Graf et al. 2010). Prolonged dark periods would likely induce nutritional stress halting the oscillations of clock components. Therefore, arrhythmicity under dark conditions would not be a direct response to darkness but rather a consequence of nutritional stress. In contrast, growth conditions used for this study mitigate this nutritional stress, enabling the study of rhythmicity under free-running conditions in DD.

#### Carotenoid biosynthesis

**Figure 10.**
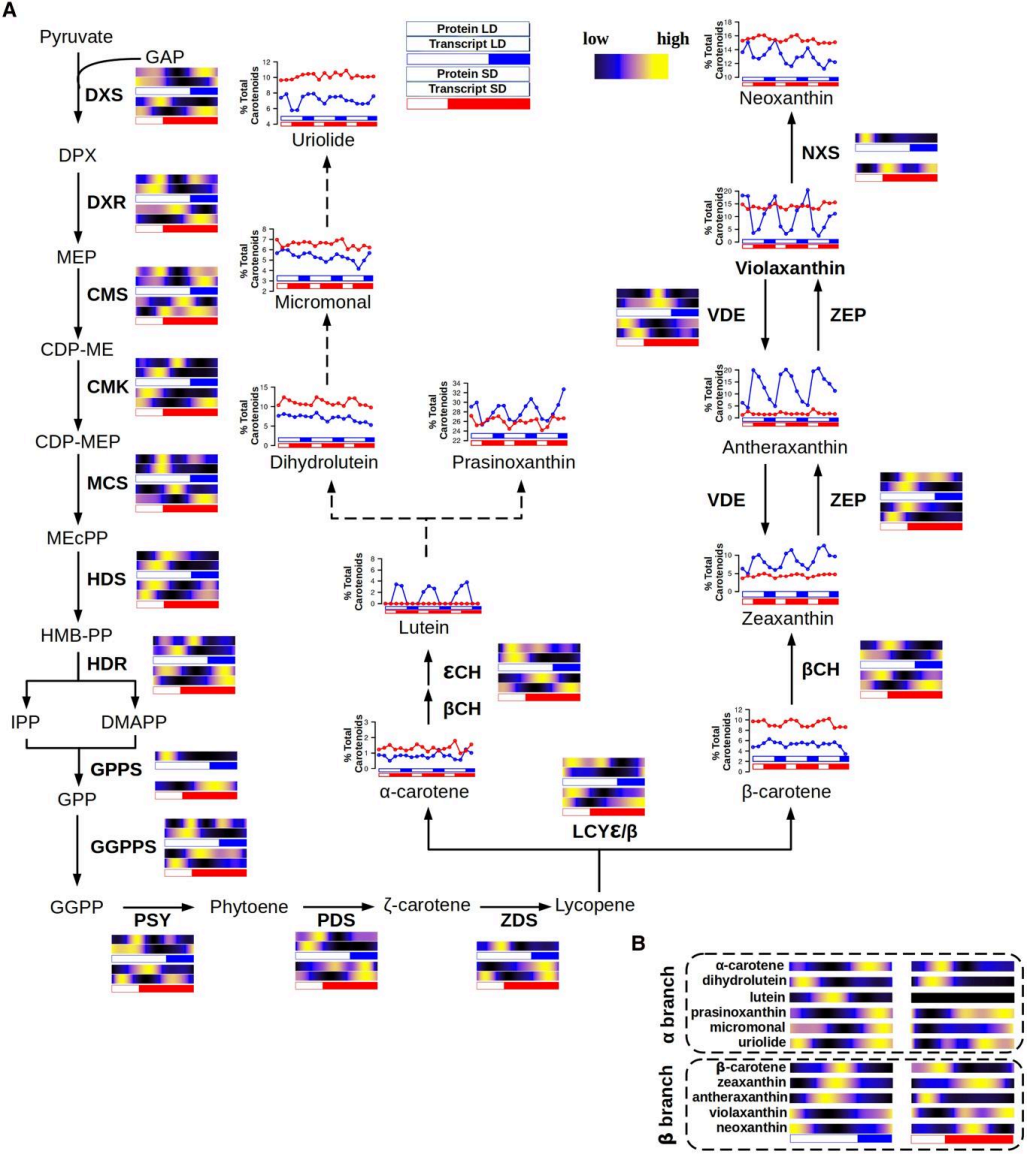
Integration of carotenoid content profiles with transcript and protein abundances for enzymes involved in carotenoids biosynthesis under long and SD conditions. **A)** Graphical representation of the carotenoid biosynthesis pathway in *Ostreococcus*. For each enzyme, its corresponding protein and transcript abundance under LD (top in blue), and SD (bottom in red) conditions, are represented using heatmaps, where black represents low, blue medium, and yellow high abundances, respectively. White rectangles represent photoperiods (light periods or days), blue- and red-filled rectangles correspond to skotoperiods (dark periods or nights) under LD and SD, respectively. Carotenoid content profiles during 3 consecutive days under LD (blue line) and SD (red line) are also displayed as percentage of total carotenoids. **B)** Heatmaps representing α branch carotenoids (top) and β branch carotenoids (bottom) under LD (left) and SD (right) conditions.

Carotenoids are pigments that play a central role in photosynthetic organisms, including phytoplankton, as essential components of the molecular machinery involved in light harvesting and photoprotection (Hashimoto et al. 2016). Transcript and protein abundance profiles of carotenoid biosynthesis genes in *Ostreococcus* were integrated with carotenoid content profiles to understand their plasticity in adjusting their rhythms to photoperiodic variations in diel cycles and their implications in optimizing light energy capture and photoprotection (Fig. 10). Most genes involved in this biological process maintained rhythmicity under all light regimes and free-running conditions indicating a strong control of the circadian clock over them.

Carotenoid biosynthesis begins with the methylerythritol phosphate (MEP) pathway, responsible for producing the carotenoid precursor’s isopentenyl diphosphate (IPP) and dimethylallyl diphosphate (DMAPP) (Fig. 10A). Most genes encoding enzymes involved in the MEP pathway, from *1-deoxy-*d*-xylulose 5-phosphate synthase* (*DXS*, *ostta02g01730*) as the first committed step, to *4-hydroxy-3-methylbut-2-en-1-yl diphosphate reductase* (*HDR*, *ostta08g01180*), peaked at ZT0 under LD and at ZT20 under SD, preceding the peaks of corresponding protein abundance by up to 4 h. Similar patterns were observed for the first enzymes in the carotenoid biosynthesis pathway leading to the committed step catalyzed by *Phytoene Synthase* (*PSY*, *ostta05g03530*) (Fig. 10A; Supplementary Fig. S11).

Carotenoid biosynthesis can follow 2 different branches, alpha and beta, depending on the activity of *lycopene epsilon/beta cyclase* (*LCYε/β*, *ostta14g00700*). The profiles of alpha-branch carotenoids, α-carotene, dihydrolutein, lutein, prasinoxanthin, micromonal, and uriolide, as well as beta branch carotenoids, β-carotene, zeaxanthin, antheraxanthin, violaxanthin, and neoxanthin, were determined over complete diel cycles under LD and SD conditions (Fig. 10B). All quantified carotenoids exhibited rhythmicity, except for violaxanthin, neoxanthin, and uriolide under SD condition (Supplementary Table S8).

The xanthophyll cycle, the interconversion between violaxanthin, antheraxanthin, and zeaxanthin as a response to light intensity (Fernández-Marín et al. 2021), was especially active under LD, although its activity was also detected under SD. The changes in these xanthophylls coincided with the accumulation of transcripts and proteins encoded by genes associated with the xanthophyll cycle, with short temporal offsets (Fig. 10A). Specifically, under LD condition, violaxanthin decreased sharply during the first half of the day (ZT0–ZT8), while antheraxanthin and zeaxanthin increased, corresponding to the accumulation of *Violaxanthin de-epoxidase* (*ostta16g00660*) transcripts and proteins with a short temporal offset. Subsequently, during the second half of the day and throughout night, antheraxanthin and zeaxanthin decreased, whereas violaxanthin increased, in agreement with the increment in gene expression and protein abundance of *Zeaxanthin epoxidase* (*ostta16g00670*).

A similar cycle was observed among alpha carotenoids, with prasinoxanthin, the most abundant carotenoid in *Ostreococcus*, decreasing as light intensity increased, concomitant with an accumulation of both lutein and dihydrolutein (Fig. 10B). The enzymes involved in the interconversion of these carotenoids remain to be identified, and the comparison of their gene expression and protein accumulation was not feasible.

#### Nitrate assimilation

**Figure 11.**
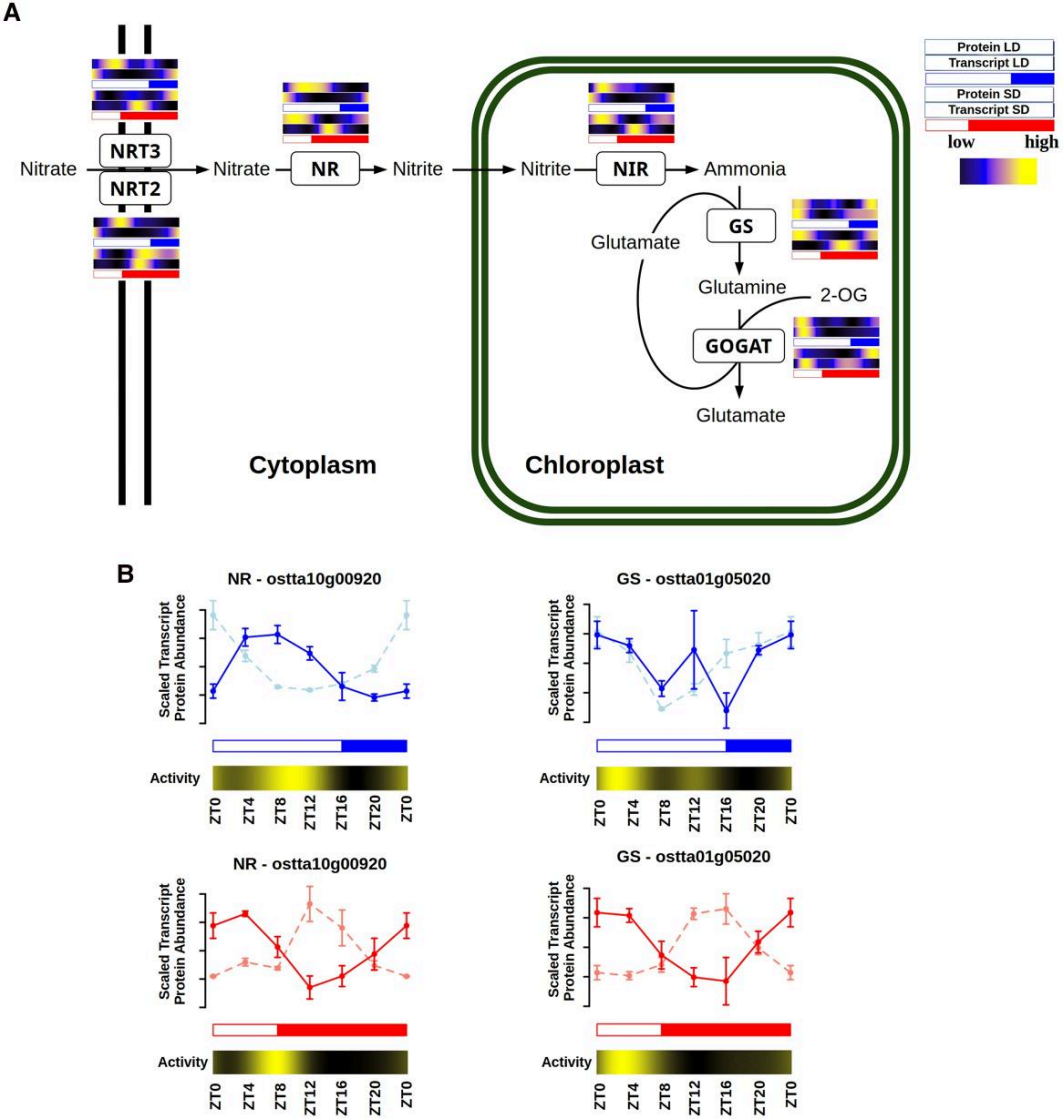
Integration of enzymatic activity profiles with transcript and protein abundances for transporters and enzymes involved in nitrate assimilation under LD and SD conditions. **A)** Graphical representation of the nitrate assimilation pathway in *Ostreococcus*. For each transporter and enzyme, its corresponding protein and transcript abundance under LD (top in blue) and SD (bottom in red) conditions are represented using heatmaps. Black represents low, blue medium, and yellow high abundances. White rectangles represent photoperiods (light periods or days), blue- and red-filled rectangles correspond to skotoperiods (dark periods or nights) under LD and SD, respectively. **B)** Mean scaled transcript (dashed line) and protein (continuous line) abundance profiles of 3 consecutive days for NR (left) and GS (right) under LD (top, blue) and SD (bottom, red) conditions. Vertical lines represent Se. Activity profiles for each enzyme under LD and SD conditions are represented using heatmaps, where black corresponds to low activity and yellow to high.

In this section, nitrate assimilation in *Ostreococcus* is examined as an example of a physiological process that exhibited long temporal offsets between transcripts and proteins. Nitrate assimilation is a fundamental process in the metabolism of photosynthetic organisms as it converts a fully oxidized nitrogen form into organic reduced nitrogen, apt for the synthesis of essential biomolecules to allow cell growth and biomass production (Sanz-Luque et al. 2015). To analyze the adaptive response of nitrate assimilation to photoperiodic variations in diel cycles and its implications for optimizing nutrient uptake and metabolism, the transcript and protein abundance profiles corresponding to this pathway in *Ostreococcus* were analyzed (Fig. 11). Genes in this pathway only maintained rhythmicity under LL being repressed under DD after both LD and SD entrainment. This suggests that, although the circadian clock controls partially these genes, there exists a strong regulation exerted by light over them.

Nitrate assimilation starts with the uptake of nitrate mediated by *Nitrate Transporters 2 and 3* (*NRT2*, *ostta10g00950* and *NRT3*, *ostta10g00940*), followed by its reduction to nitrite by *nitrate reductase* (*NR*, *ostta10g00920*). Nitrite is further reduced to ammonium by *nitrite reductase* (*NIR*, *ostta10g00930*). The central part of ammonium assimilation is played by the *glutamine synthetase* (*GS*, *ostta01g05020*) and *glutamine oxoglutarate aminotransferase* (*GOGAT*, *ostta14g01900*) cycle, which incorporates inorganic ammonium nitrogen first into glutamine and hence into glutamate, the central precursor for the biosynthesis of all other nitrogen-containing biomolecules (Fig. 11A; Supplementary Fig. S11).

Under LD conditions, *NRT2/3*, *NR*, and *NIR* gene expression reached their maximum at dawn (ZT0), while their protein abundances peaked 8 h later at midday (ZT8), coinciding with the time point of maximum irradiance. *GS* and *GOGAT* gene expression and protein abundance peaked at the beginning of the day (ZT4) without noticeable temporal offsets. However, a slight increase in protein abundance was detected at the end of the day (ZT12) for both GS and GOGAT (Fig. 11A; Supplementary Fig. S11). In contrast, under SD conditions, all genes encoding the transporters and enzymes involved in nitrate assimilation showed their maximum expression at midnight (ZT12–ZT16), preceding their protein abundance peaks, reached at dawn (ZT0) or midday (ZT4), by 8 h or more. Notably, *GOGAT* gene expression displayed a bimodal pattern under SD conditions, maintaining the peak observed under LD at ZT4 besides a new peak at ZT16 (Fig. 11A; Supplementary Fig. S11).

To validate these temporal offsets between transcripts and proteins, NR and GS activities were measured throughout complete diel cycles under both LD and SD conditions. Enzyme activity profiles were significantly rhythmic (Supplementary Table S9). NR maximal catalytic activity reached its peak at ZT8 under both LD and SD conditions, coinciding with the protein abundance peak under LD and exhibiting a short forward temporal offset under SD. Similarly, GS activity peaked at ZT0 under both LD and SD, concomitant with the protein abundance peaks (Fig. 11B).

## Discussion

Seasonality plays a key role in the natural geographical growth dynamics of marine phytoplankton, including the model picoalga *Ostreococcus* (Bolaños et al. 2020). Nevertheless, the molecular rhythms underpinning its responses to photoperiodic variations remained to be characterized. In this study, a multiomics approach has been adopted to unravel the plasticity in the orchestration between transcriptome and proteome rhythms governing cyclic physiological responses to changes in photoperiod length.

Transcriptomic analysis revealed that photoperiodic variations had no effect over the identity of rhythmic genes, which comprised 80% of the transcriptome. Arrhythmic genes were associated with stress responses and exhibited almost complete repression. These genes could become rhythmic once highly expressed under the corresponding stress condition resulting in a full rhythmic transcriptome. Our results are in agreement with previous studies (Monnier et al. 2010; Zones et al. 2015) indicating that transcriptome rhythmicity in chlorophyte phytoplankton is much higher than in other organisms such as *A. thaliana* 30% to 50% (Bläsing et al. 2005), *Solanum tuberosum* 18% to 45% (Hoopes et al. 2022), *Drosophila melanogaster* 24% (Ma et al. 2021), or *Mus musculus* 3% to 10% (Miller et al. 2007). Nevertheless, only a small fraction of the transcriptome was rhythmic under LL and DD free-running conditions. These genes were significantly involved in photosynthesis and chloroplast organization, indicating a predominant control by the autonomous circadian clock. Gene rhythmicity is strongly influenced by alternating light/dark cycles in this picoalga. For instance, DNA replication genes maintained rhythmicity under LL but were repressed under DD, while ribosome biogenesis genes exhibited rhythmicity under DD but lost it under LL. Generally, LL had a more detrimental effect on transcriptome rhythmicity than DD, in agreement with studies in plants such as *Medicago truncatula* (Wang et al. 2021), *S. tuberosum* (Hoopes et al. 2022), and *Hordeum vulgare* (Müller et al. 2020). Notably, rhythmic patterns under free-running conditions differed significantly from those under diel cycles. LL conditions reduced rhythmic amplitude, likely due to decreased cellular synchrony (Supplementary Fig. S13), as observed in *Arabidopsis* leaves (Yakir et al. 2011; Wenden et al. 2012), consistent with the cell-autonomous character of circadian clocks. Typical responses of “nocturnal character were found, such as advanced and delayed phases under DD and LL, respectively (Hundahl et al. 2012; Imai et al. 2020). Indeed, most transcripts peaked during the night, consistent with the “Escape from light theory (Pittendrigh 1993). During transcription, DNA is unwound leaving it in an exposed state more susceptible to light-induced damage. To mitigate this risk, *Ostreococcus* might concentrate transcription predominantly during the night.

Although rhythmic genes were almost identical under LD and SD conditions, significant effects on their profiles were observed. Specifically, advanced phases and reductions in amplitude under SD were found, as demonstrated at the level of individual genes in the model chlorophyte microalgae *Chlamydomonas reinhardtii* (Serrano et al. 2009). Simulations showed that two-thirds of the rhythmic genes with a single peak respond to changes in photoperiod by gradually adjusting their phases while the remaining third adjust their phases according to a more complex mechanism. Another response to photoperiod shortening was the emergence of rhythmic bimodal gene expression profiles. This type of rhythmicity has been suggested to be induced by the cyclic occurrence of tides in coastal environments resulting in circatidal rhythms in benthic diatoms (Bilcke et al. 2021). However, our experimental design did not simulate tidal conditions and *Ostreococcus* is described as a planktonic picoalga. Moreover, the exact period of circatidal rhythms is 12.4 h, which would result in a daily phase shift of ∼1 h, which was not observed in our data. If these expression patterns were truly circatidal, bimodality should have been maintained under free-running conditions. However, they disappeared resulting in the maintenance of only one of the peaks under LL and the other one under DD. This suggests the combination of 2 distinct profiles, 1 dependent on light and the other 1 on dark. Model simulations showed how, under LD conditions, the 2 distinct profiles overlap in time, producing a single peak, while as the photoperiod shortens they become out of phase producing a bimodal profile under SD conditions. Bimodal rhythmic gene expression profiles have also been identified in plants (Filichkin et al. 2011) and animals (Weger et al. 2021).

To further explore the seasonal responses in *Ostreococcus* at the molecular level, proteomic data were generated and integrated with the transcriptomic data. Our 48% proteome coverage represented an improvement compared to previous studies, such as 12% in *A. thaliana* (Seaton et al. 2018), 30% in *D. melanogaster* (Wang et al. 2020), and 9% in *M. musculus* (Chiang et al. 2014), and lies between published studies in *Ostreococcus* with 27% in Le Bihan et al. (2011) and 85% in Kay et al. (2021). In contrast to the high transcriptome rhythmicity, a drastic reduction in proteome rhythmicity was found consistent with (Kay et al. 2021). In general, no coincidences between rhythmic protein and transcript abundance profiles were observed with temporal offsets of several hours between them. This observation pointed to the decoupling of transcription and translation and to the existence of a significant regulation over translation initiation (Freeney et al. 2016; Kay et al. 2021). Temporal offset lengths were not uniform suggesting the existence of a differential posttranscriptional regulation for each specific biological process. Similar temporal shifts between transcripts and proteins have been reported in other organisms, such as *M. musculus* (Robles et al. 2014). Notably, a photoperiodic effect on transcript/protein temporal offsets was detected, with longer offsets under SD than LD. Moreover, in SD-entrained cultures, significantly longer temporal offsets for transcripts peaking during the skotoperiod were found.

Physiological measurements were integrated with transcriptome and proteome dynamics, aiming to elucidate the full temporal orchestration at different molecular levels underlying the responses to changes in photoperiod length. Cell-cycle progression, photosynthesis, starch accumulation, carotenoid biosynthesis, and nitrate assimilation presented advanced rhythmic patterns under SD compared to LD conditions, in agreement with transcriptome and proteome responses to photoperiod shortening. In line with the described *Ostreococcus* algal blooms in spring and summer (Bolaños et al. 2020; Chen et al. 2021; Mondal and Banerjee 2022), in LD-entrained cultures, increased number of cells entering the S phase, enhanced photosynthetic activity, greater starch accumulation, and a more active xanthophyll cycle were patent.

While temporal offsets of several hours have been observed between transcripts and proteins, almost coincident protein abundance profiles and rhythmic physiological measurements occur. These temporal offsets shed light on the physiological significance of the observed transcriptional programs over complete diel cycles. For instance, considering the temporal offset of 8 h under LD between the transcript and protein abundances of NR, an enzyme that requires light for its activation, peaking of its transcript at dawn (ZT0) suffices to make the protein peak coincide with maximum light irradiance (ZT8). However, under SD, with a photoperiod of only 8 h, to ensure that the protein peaks at ZT4 (the point of maximum light irradiance), the corresponding transcript must reach its maximum expression level around midnight (ZT12–ZT16). This suggests the existence of molecular mechanisms that allow *Ostreococcus* to adjust its transcriptome timing depending on the photoperiod length, considering the temporal offsets between transcripts and proteins to ensure that proteins are available at the appropriate moment of the day. Such photoperiodic plastic orchestration between transcriptome, proteome, and physiological timing might play a crucial role in the ability of *Ostreococcus* to thrive and optimize its physiological processes accordingly under photoperiodic variations in diel cycles.

**Figure 12.**
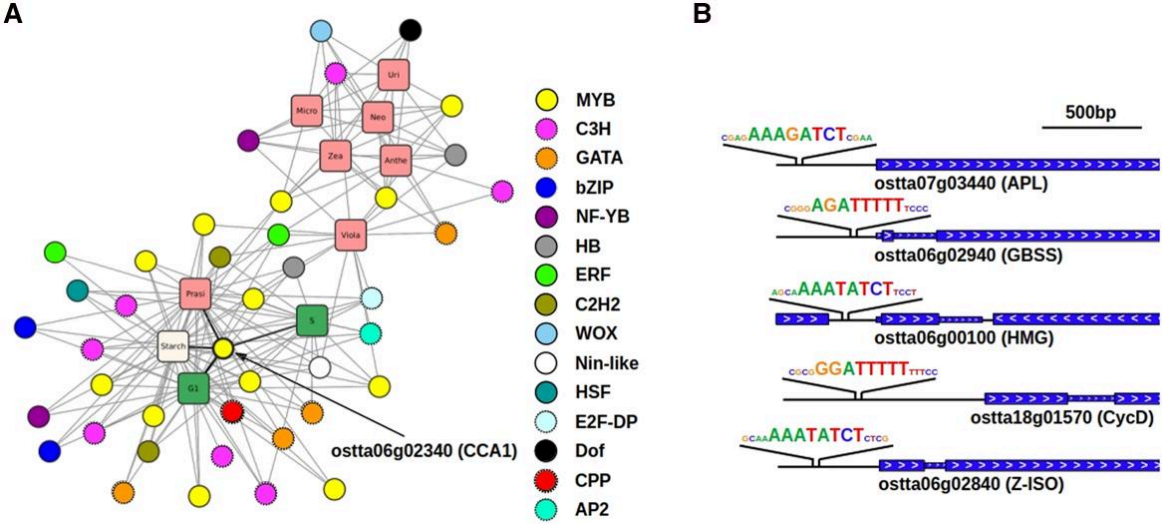
Integration of transcription factor transcriptomic data and physiological measurements. **A)** Network representation of a sPLS model integrating transcription factor gene expression as predictor variables with physiological measurements as response variables. Circular nodes represent genes encoding transcription factors from different families, indicated by distinct colors. Rounded square nodes represent physiological measurements: S and G1 cell cycle phases (in green), Starch content (in white) and carotenoid content (in pink) prasinoxanthin (Prasi), violaxanthin (Vio), antheraxanthin (Anthe), zeaxanthin (Zea), neoxanthin (Neo), micromonal (Micro), and uriolide (Uri). An edge between a transcription factor node and a physiological measurement node is drawn when the expression profile of the corresponding transcription factor has a significantly high predictive power over the corresponding physiological measurement, suggesting a potential regulatory role. The CCA1 orthologue (*ostta06g02340*) is identified as a potential central regulator. **B)** Identification of the EE motif, recognized by the transcription factor CCA1, in the promoters of genes encoding key proteins involved in starch metabolism, cell cycle progression, and carotenoid biosynthesis.

A network model identified specific transcription factor (TF) families as potential key regulators of the rhythmic physiology detected in *Ostreococcus* such as the families MYB, DOF, bZIP, and CPP. This was further supported by the TFBS enrichment analysis, which identified significant DNA motifs associated to these TF families in specific gene clusters (Fig. 4). Notably, the CCA1 orthologue, *ostta06g2340*, was found to have a significantly high predictive power over starch content, cell-cycle phases, and carotenoid content suggesting a regulatory role over the corresponding biological processes (Fig. 12A). Furthermore, the EE motif, recognized by this transcription factor in *Arabidopsis*, was found significantly enriched in genes peaking at the end of the day under LD conditions, some of which encode key proteins in starch metabolism, cell-cycle progression, and carotenoid biosynthesis (Fig. 12B). This indicates a conserved role of this transcription factor in regulating the core of the circadian clocks in *Ostreococcus* and *Arabidopsis*.

## Materials and methods

### Culture conditions

*O. tauri* (*Ostreococcus*) sequenced strain *RCC4221* was used for all experiments. The growth medium was prepared using artificial sea water (24.55 g NaCl, 0.75 g KCl, 4.07 g MgCl_2_·6H_2_O, 1.47 g CaCl_2_·2H_2_O, 6.04 g MgSO_4_·7H_2_O, and 0.21 g NaHCO_3_ per 1 L distilled water) supplemented with 1 mL of Solution I (100 g NaNO_3_ in 250 mL distilled water), 1 mL of Solution II (700 mg Na_2_HPO_4_ and 2.5 g K_2_HPO_4_ in 250 mL distilled water), 1 mL of Solution III with trace metals (2.68 g NH_4_Cl, 5.2 g Fe-EDTA, 37.2 g Na_2_-EDTA, 23 mg ZnSO_4_, 14 mg CoSO_4_, 7.89 mg Na_2_ MoO_4_ ·2H_2_O, 2.5 mg CuSO_4_, 1.7 mg H_2_SeO_3_, and 180 mg MnCl_2_·4H_2_O in 500 mL distilled water), and 1 mL of f/2 vitamin solution.

Experiments were performed in photochemostats consisting of water jacketed bubble columns with 2 L capacity (7 cm diameter and 50 cm height) containing 1.8 L of cell suspension continuously sparged with air to ensure culture homogenization. The flow of water through the jacket, from an external cooling device, maintained temperature at 20 °C. A pH probe was submerged into the culture and connected to a pH meter serving as input to a LabJack that controlled an electrovalve, allowing on demand injection of CO_2_ into the air stream entering the culture to maintain the pH at 8.0. Each photochemostat was illuminated during the corresponding light period using 6 Phillips PL-32 W/840/4p white-light fluorescent lamps. The illuminating system simulated the progressive light intensity increase and decrease during solar daylight cycles with a maximum light irradiance of 1,500 μE m^−2 ^s^−1^. Under LL conditions light intensity was maintained to this maximum level. Each photochemostat was kept within a wooden case and covered by a completely opaque fabric, to ensure that the illumination is only provided by the fluorescent lamps. The illumination regime was 16 h of light and 8 h of darkness for LD condition and 8 h of light and 16 h of darkness for SD condition. Photochemostats were operated in continuous mode adding fresh medium continuously during the light period at a flow rate of 45 mL h^−1^ with a peristaltic pump. Since the photoperiod under LD is twice as long as under SD, the dilution rates were 0.4 d^−1^ under LD and 0.2 d^−1^ under SD. Excess culture was removed at the same rate by the overflow in order to keep a constant volume.

For the comparative analysis of starch content, Ostreococcus cells were grown following the methodology outlined in O’-Neill et al. (2011).

### Sample collection, RNA extraction, and purification

Samples were collected for 3 consecutive days every 4 h at ZT0, ZT4, ZT8, ZT12, ZT16, and ZT20 where Zeitgeber time *N* (ZTN) marks the time point *N* hours after the beginning of the photoperiod simulating dawn. Specifically, ZT0 was collected immediately after lights were on; and ZT8 (under SD conditions) and ZT16 (under LD conditions) were collected immediately after light were off. Subsequently, cultures were transferred to free-running conditions consisting of LL and DD. No samples were collected during the first day to allow cultures acclimation, and then samples were collected for 2 consecutive days every 4 h at CT0, CT4, CT8, CT12, CT16, and CT20 where Circadian time *N* (CTN) denotes the time point *N* hours after the subjective dawn. For each time point, 50 mL of culture were collected for RNA extraction. Cells were washed with phosphate-buffered saline (PBS) solution using centrifugation for 1 min at 13,000 × *g* and 4 °C. After supernatant removal, cells were immediately flash frozen in liquid nitrogen and stored at −80 °C.

Frozen pellets were resuspended in 400 μL of disruption buffer (García-Domínguez and Florencio 1997) and added to a 1.5 mL Eppendorf tubes (RNAse free and phenol-proof) containing 400 μL of phenol:chloroform 1:1 and 100 μL of acid washed glass beads (0.25 to 0.3 mm diameter; Braun, Melsungen, Germany). Mechanical disruption was performed for 30 min by alternating cycles of vortexing for 60 s and ice incubation for 60 s. Extracts were centrifuged at 4 °C for 15 min at 13,000 × *g*, the upper aqueous phase was collected and mixed with 400 μL of phenol:chloroform 1:1 and centrifuged in the same conditions for 5 min. This process was repeated a total of 3 times. In the last wash, only chloroform was used to remove phenol from the samples. The supernatant was incubated overnight at −20 °C in a solution of 80 μL 10 m LiCl and 550 μL 100% EtOH for RNA precipitation. Subsequently, samples were centrifuged for 10 min at 13,000 × *g* at 4 °C. Pellets were dried to avoid EtOH contamination. Further RNA purification was performed using the Isolate II RNA Plant Kit (Bioline) following the manufacturer instructions. RNA concentration and integrity were measured using a Bioanalyzer 2100 (Agilent RNA 6000 Nano Kit).

### RNA-seq data generation and analysis

Library preparation was carried out following the manufacturer's instructions and sequencing was performed on an Illumina NextSeq500 sequencer for 3 replicates for each time point under LD and SD conditions. Approximately 10 million 75 nt long single end reads were generated for each sample. RNA-seq data were analyzed using our pipeline MicroAlgae RNA-seq and Chip-seq AnalysiS (MARACAS) (Romero-Losada et al. 2022). Specifically, the high quality of the sequencing data was assessed using the software package FASTQC. The *O. tauri* genome sequence and annotation v3.0 (https://phycocosm.jgi.doe.gov/Ostta4221_3/Ostta4221_3.home.html) were used as reference genome. Reads were mapped to the reference genome with HISAT2 (Kim et al. 2019). Transcript assembly and gene expression estimation measured as fragments per kilobase of exon and millions of mapped reads (FPKM) were performed using StringTie2 (Kovaka et al. 2019) and the bioconductor R package Ballgown (Frazee et al. 2015). PCA and HC were performed using the R package FactoMineR (Lê et al. 2008). The R code for this analysis is available from the GitHub repository SANDAL (https://github.com/fran-romero-campero/SANDAL).

RT-qPCR for CCA1 was performed using the forward primer (CTAGTACGTCGTCGAGC) and the reverse primer (CCACGAACGGACTCAT), as internal reference we used EF1 alpha with forward primer (GACGCGACGGTGGATCAA) and reverse primer (CGACTGCCATCGTTTTACC) following the methodology described previously in Corellou et al. (2009).

### Sample collection and protein extraction

Sample collection was performed as described for RNA analysis for 3 consecutive days under LD and SD conditions. For cell disruption, 1 mL of TRIsure, 100 μL of acid washed glass beads (0.25 to 0.3 mm diameter) and 40 μL of protein inhibitor cocktail (25×) were added onto frozen pellets, followed by 3 disruption cycles (60 s agitation—60 s incubation on ice) using a Mini-Beadbeater (BioSpec Products). Proteins were extracted using TRIsure Reagent (Sigma-Aldrich), according to the manufacturer's instructions. The resulting proteins pellets were resuspended with 2 mL of 0.3 m guanidine solution in 95% EtOH using 10 sonication cycles (30 s sonication—30 s of incubating at 4 °C) and then centrifuged at 4 °C during 5 min at 8,000 × *g*. This washing process was repeated twice, followed by 2 additional washes using 90% EtOH. The final pellets were resuspended in NH_4_HCO_3_ 50 mm/0.2% Rapidgest (Waters) and total proteins were quantified using a Qubit device. For each sample, 50 μg of proteins were incubated with dithiothreitol (final concentration 4.5 mm) for 30 min at 60 °C. Iodoacetamide was added to a final concentration of 10 mm and incubated for 30 min, under total darkness at room temperature. An overnight trypsin treatment was done at 37 °C in a 1:40 trypsin:protein. Subsequently, formic acid was added and incubated at 37 °C for 1 h. Finally, 2% acetonitrile (v/v) were added to reach a concentration of the digested sample of ∼0.5 μg of protein/μL.

### Sequential windowed acquisition of all theoretical mass spectra proteomics data generation and analysis

We utilized a label free quantification platform that employs independent data acquisition called Sequential Windowed Acquisition of all THeoretical Mass Spectra (SWATH-MS) (Ludwig et al. 2018) using a time-of-flight (TOF) triple quadrupole hybrid mass spectrometer MS (5600 plus, Sciex) equipped with a nanoelectrospray source coupled to a nano-HPLC Eksigent model 425. The Sciex software Analyst TF 1.7 was used for the equipment control and data acquisition. Peptides were first loaded onto a trap column (Acclaim PepMap 100 C18, 5 µm, 100 Å, 100 µm id × 20 mm, Thermo Fisher Scientific) under isocratical order in 0.1% formic acid/2% acetonitrile (v/v) at a flow rate of 3 μL/min for 10 min. Subsequently, they were eluted on a reversed-phase analytical column, Acclaim PepMap 100 C18, 3 µm, 100 Å, 75 µm id × 250 mm, Thermo Fisher Scientific, coupled to a PicoTip emitter (F360-20-10-N-20_C12 from New Objective). Formic acid 0.1% (v/v) was used as Solvent A and 2% acetonitrile with formic acid 0.1% (v/v) as Solvent B. Peptides were eluted with a linear gradient of 5% to 35% (v/v) of Solvent B in 120 min at a flow rate of 300 nL/min. The source voltage was selected at 2,600 V and the temperature was maintained at 100 °C. Gas 1 was selected at 20 PSI, Gas 2 at 0, and curtain gas at 25 PSI.

For proteins identification, a TOF-MS with a scan window of 400 to 1,250 m/z (accumulation time of 250 ms) was used followed by 50 MS/MS with a scan window of 230 to 1,500 m/z (accumulation time of 65 ms) and with a cycle time of 2,574 s.

Spectral libraries for LD and SD conditions were constructed by making one run with a mixture of the biological replicates corresponding to each time point. ProteinPilot v5.0.1 software (Sciex) was used to identify the proteins in the library. A pooled search of all runs was performed. The parameters of the Paragon method were: trypsin as enzyme and iodoacetamide as cysteine alkylating agent. The *O. tauri* annotated proteome v3.0 file from: https://phycocosm.jgi.doe.gov/Ostta4221_3/Ostta4221_3.home.html linked to a Sciex Contaminants database were used in library construction. A false positive analysis (FDR) was performed and those with FDR < 0.01 were considered.

For each sample, the equivalent of 1 µg of digested protein was injected into each run. Previously, the equipment was self-calibrated using a standard, MS synthetic peptide calibration kit from Sciex, to control sensitivity and chromatographic conditions. Protein identification and quantification were performed using SWATH runs with 60 ms of accumulation time and 3.7 s of cycle time. Three technical replicates for each 1 of the 3 biological replicates were analyzed resulting in 9 replicates per time point.

The generated libraries (1% FDR) were analyzed using the Sciex software PeaKView 2.2 with the microapp SWATH 2.0, together with the data obtained from the SWATH runs. Using this software, the chromatographic traces of the ions were extracted and dumped into the Marker view 1.2.1.1 program where the list of identified proteins with their corresponding areas was generated. The parameters for extraction of ions and areas were: 10 peptides per protein, 7 transitions of each peptide, threshold of confidence of the peptides set at 90, and FDR 1%. The R package NormalyzerDE (Willforss et al. 2019) was used to perform Quantile normalization. Data were imputed with mean imputation method between the 9 technical and biological replicates.

### Cell cycle data acquisition and analysis

A volume of 1.5 mL of cell suspension was harvested for each time point under LD and SD conditions and diluted 1:10 in PBS. Two milliliter of these dilutions were centrifuged and cells in the pellets were fixed with 10 mL of 100% EtOH before storage at −20 °C for, at least, 24 h. After fixation, cell suspensions were centrifuged for 5 min at 3,500 × *g* (room temperature) and resuspended in 1 mL of PBS, washed once with PBS and sonicated for 3 min in an Ultrasonic Cleaner (JSP, US21, ultrasonic power 50 W), in order to eliminate cell clumps and aggregates before staining. In the staining process, 2 μL of the Vibrant Dye Cycle Green (V35004, Thermo Fisher) (10 μM final stain concentration) were added to each sample and incubated 30 min (37 °C) for selective DNA labeling. After incubation, cells were washed and transferred to ﬂow cytometry tubes for cell cycle analysis. Flow cytometry acquisition was performed with a BD FACS Canto II (BD Biosciences) where stained DNA was excited by a 488 nm laser and emission was collected in a 530/30 nm photomultiplier tube (PMT). Flow rate was low and linear amplification was established for the acquisition. Data were analyzed using FlowJo v.10.6.1 (Becton Dickinson & Company BD). Analysis was performed using the Watson pragmatic algorithm provided by FlowJo (Watson et al. 1987) to adjust the data to the model.

### Confocal microscopy

Confocal microscopy images of *Ostreococcus* cells were acquired using a spectral Laser Scanning Confocal Microscope (Olympus FLOUVIEW FV3000). Excitation was performed with a 488 nm laser. Emission signals were detected within the green channel (500 to 540 nm, gain 504) and red channel (650 to 750 nm, gain 432).

### Photosynthetic activity: sample collection and data acquisition

Fresh cultures were harvested for each time point under LD and SD conditions. Samples were diluted 1:1 with growth medium and incubated at 20 °C in total darkness during 10 min. In order to analyze photosynthetic parameters, pulse-amplitude-modulation (PAM) fluorometry measurements were performed using a Waltz DUAL-PAM-100. After darkness incubation, non-actinic modulated light (450 nm, 2.8 μE m^−2^ s^−1^) was turned on to measure the fluorescence basal level, F_0_. A saturating red light pulse (655 nm and 5,000 μE m^−2^ s^−1^) was applied for 400 ms to determine the maximum fluorescence level, *F*_m_. Fv/Fm, the maximum potential quantum efficiency of PSII when all reaction centers are open, was calculated as Fv/Fm = (*F*_m_ − *F*_0_)/*F*_m_.

### Starch content determination

At each time point, 50 mL of fresh culture were harvested, centrifuged at 7,000 × *g* for 10 min. The pellets were washed with 1% ammonium formiate (p/v) to eliminate salts, and lyophilized. Approximately 2 to 3 mg of lyophilized biomass were added to hermetic tubes containing 1 mL of glass beads (0.25 to 0.3 mm diameter) and 2 mL of chloroform:methanol (2:1). Three disrupting cycles (60 s agitation−60 s incubation on ice) were applied using Mini-Beadbeater (BioSpec Products). Then, cell extracts were separated from the beads and saved in new tubes. Cell extracts were centrifuged for 4 min at 13,000 × *g* and the supernatant was discarded. Pellets were washed with chloroform:methanol (2:1) until they became white ensuring pigments and lipids removal. Pellets were dried and the spectrophotometric protocol described in García-Cubero et al. (2018) was used to determine starch content.

### Carotenoid content determination

Four milligrams of lyophilized biomass were added to a hermetic tube containing 1 mL of glass beads (0.25 to 0.3 mm diameter) and 1 mL of pure acetone. Three disrupting cycles (60 s agitation−60 s incubation on ice) were applied using Mini-Beadbeater (BioSpec Products). Carotenoids extraction was achieved following the protocol developed by Del Campo et al. (2004). A Hitachi HPLC (Elite LaChrom), equipped with a photodiode-array detector (Hitachi L-2455) was used. Separation was performed on a Waters NovaPak C-18 (3.9 × 150 mm, 4 µm particle size, 60 Å pore size) column. The eluents used to create a gradient through the mobile phase were: Eluent A (0.1 m ammonium acetate and 15:85 v/v H_2_O–methanol) and Eluent B (44:43:13 v/v methanol–acetonitrile–acetone). Temperature was maintained constant (20 °C) during the whole process and eluents flowed at 800 μL min^−1^. Different carotenoids were identified according to retention times and absorption profiles of known carotenoids. Quantification was determined as a percentage of the total peak area corresponding to carotenoids.

### Identification of genes, proteins and physiological measurements exhibiting rhythmic patterns and their statistical comparative analysis

The bioconductor R package rhythmicity analysis incorporating nonparametric methods (RAIN) (Thaben and Westermark 2014) was used to identify genes, proteins, and physiological measurements exhibiting rhythmic patterns. A Benjamini–Hochberg corrected *P*-value threshold equal to 0.05 was used in all the cases under study. Rhythmic patterns with a single peak per day were identified by setting the period parameter to 24 h. Rhythmic patterns exhibiting 2 peaks per day were identified by setting the period parameter to 12 h.

For statistical comparison, rhythmic patterns were fitted to a co-sinusoidal curve characterized by 3 parameters: mesor, amplitude, and phase. The statistical significance of the differences in amplitude and phase between different groups was performed using the R package CircaCompare (Parsons et al. 2020). A Benjamini–Hochberg corrected *P*-value threshold of 0.05 was used to determine statistically significant differences.

The nonparametric method implemented in RAIN was used first since it searches for any type of rhythmic patterns. The methods in CircaCompare were also employed identifying approximately the same rhythmicity. Once it was confirmed that our data could be further analyzed using the parametric methods in CircaCompare, significance analysis of differences in phases and amplitudes were performed using CircaCompare.

The significance of the global differences in these parameters was assessed using the Mann–Whitney–Wilcoxon nonparametric test implemented in the R function wilcox.test. Phase differences between transcript and protein expression profiles were computed assuming that transcript peaks precede those of proteins.

Significant differences between specific features not related to rhythmicity were also assessed using the Mann–Whitney–Wilcoxon nonparametric test implemented in the R function wilcox.test.

### Functional annotation of gene sets

Functional enrichment analysis over different gene sets was performed using our online tool AlgaeFUN, microALGAE FUNctional enrichment tool (Romero-Losada et al. 2022) that in turn is based on the bioconductor packages clusterProfiler, enrichplot, and pathview (Luo and Brouwer 2013; Wu et al. 2021) and the functional annotation package developed by our group org.Otauri.eg.db for *O. tauri* (https://github.com/fran-romero-campero/AlgaeFUN/tree/master/packages/annotation_packages).

### TFBS enrichment analysis

The identification of plant specific TFBS significantly enriched in the promoters of the genes peaking at specific time points was performed using the software for motif discovery and next generation sequencing analysis Hypergeometric Optimization of Motif EnRichment (Heinz et al. 2010). The function *findMotifsGenome.pl* was applied with the parameter for plant specific TFBS*-mset plants*. The length of the gene promoter was fixed to 500 nt upstream of the start codon and the motif lengths to 6 and 8 nt.

### Integrative model based on sparse partial least squares

The R package mixomics (Rohart et al. 2017) was used to develop a network model for the integration of physiological and transcription factor gene expression data based on the multivariate projection method sparse partial least squares (sPLS) (Lê Cao et al. 2009). Physiological data corresponding to cell cycle phases, starch and carotenoid contents were considered as response variables and gene expression data for transcription factors as predictors. The model was constructed with the function *spls* in regression mode and keeping 3 components in the projection. The function *network* with a cutoff of 0.45 was applied to generate the network representation of the model. The software tool Cytoscape (Shannon et al. 2003) was used for the graphical representation of this network using the *yFiles Organic Layout*.

### Enzyme activities

At each time point, 1 mL of fresh culture was used to measure enzyme activities. Specifically, NR and GS activities were measured as previously described by Herrero et al. (1981) and García-Domínguez and Florencio (1997), respectively.

### Accession numbers

The *O. tauri RCC4221* genome sequence used in this article can be found in the GenBank data libraries under accession number GCA_000214015.2.

## Supplementary Material

koaf033_Supplementary_Data

## Data Availability

RNA-seq data generated in this study are freely available from the Gene Expression Omnibus database under the accession number GSE155535. The SWATH-MS proteomics data generated in this study have been deposited to the ProteomeXchange Consortium via the PRIDE (Perez-Riverol et al. 2022) partner repository with the dataset identifier PXD046992. The data analysis code developed using the statistical programming language R is freely available from the following GitHub repository SANDAL: https://github.com/fran-romero-campero/SANDAL. The results presented in this paper can be further explored using the online tool MINOTAUR: https://greennetwork.us.es/MINOTAUR/.

## Supplementary data

The following materials are available in the online version of this article.

Supplementary Figure S1. Experimental design and transcriptomic RNA-seq data reliability.

Supplementary Figure S2. Biological processes significantly enriched in the genes exhibiting rhythmicity under alternating light/dark cycles and constant light.

Supplementary Figure S3. Biological processes significantly enriched in the genes exhibiting rhythmicity under alternating light/dark cycles and constant dark.

Supplementary Figure S4. Gene expression profiles of clock components and light receptors.

Supplementary Figure S5. Functional enrichment over circadian genes after LD and SD entrainment.

Supplementary Figure S6. Transcriptional temporal program of the distribution of biological processes over diel cycles under LD conditions (16 h light/8 h dark).

Supplementary Figure S7. Transcriptional temporal program of the distribution of biological processes over diel cycles under short day conditions (8 h light/16 h dark).

Supplementary Figure S8. Validation of predicted emergence of bimodal rhythmic profiles under neutral day conditions.

Supplementary Figure S9. Proteomic data normalization and reliability.

Supplementary Figure S10. Transcript/protein phase offsets did not correlate to any biochemical properties computed from protein sequences.

Supplementary Figure S11. Heatmaps integrating transcript and protein abundance under LD and SD conditions for different biological processes.

Supplementary Figure S12. Gene expression and starch content under constant dark.

Supplementary Figure S13. Reductions in amplitude under free-running conditions can be explained by a decline in culture synchrony.

Supplementary Table S1. Rhythmic genes under different light regimes.

Supplementary Table S2. Set of rhythmic genes peaking at each time point under long- and short-day conditions.

Supplementary Table S3. Set of rhythmic genes with 2 peaks.

Supplementary Table S4. Protein abundances under long- and short-day conditions.

Supplementary Table S5. Rhythmic proteins under long- and short-day conditions.

Supplementary Table S6. Cell cycle data under long- and short-day conditions.

Supplementary Table S7. Starch content data under long- and short-day conditions.

Supplementary Table S8. Carotenoid content data under long- and short-day conditions.

Supplementary Table S9. Nitrate reductase and glutamine synthetase activities under long- and short-day conditions.

## Dive Curated Terms

The following phenotypic, genotypic, and functional terms are of significance to the work described in this paper:

APL Gramene: ostta07g03440
APL Araport: ostta07g03440

CRY3 Gramene: ostta01g06470
CRY3 Araport: ostta01g06470

SBPASE Gramene: AT3G55800
SBPASE Araport: AT3G55800

HDR Gramene: AT4G34350
HDR Araport: AT4G34350

PETC Gramene: AT4G03280
PETC Araport: AT4G03280

CAROTENOID CHEBI: CHEBI:23044

starch CHEBI: CHEBI:28017

CPP Gramene: ostta09g04220
CPP Araport: ostta09g04220

starch content Planteome: TO:0000696

CCA1 Gramene: ostta06g02340
CCA1 Araport: ostta06g02340

TOC1 Gramene: ostta13g01820
TOC1 Araport: ostta13g01820

PRR7 Gramene: AT5G02810
PRR7 Araport: AT5G02810

ATPD Gramene: AT4G09650
ATPD Araport: AT4G09650

CRY1 Gramene: ostta15g0100
CRY1 Araport: ostta15g0100

TFBS Gramene: Transcription Factor Binding Sites (TFBSs)
TFBS Araport: Transcription Factor Binding Sites (TFBSs)

nitrate CHEBI: CHEBI:17632

Gigantea Gramene: BRADI2G05226
Gigantea Araport: BRADI2G05226

PRR5 Gramene: AT5G24470
PRR5 Araport: AT5G24470

NIR Gramene: ostta10g00930
NIR Araport: ostta10g00930

PSY Gramene: ostta05g03530
PSY Araport: ostta05g03530

PsbQ Gramene: ostta16g01620
PsbQ Araport: ostta16g01620

UVR2 Gramene: ostta03g05620
UVR2 Araport: ostta03g05620

CycD Gramene: ostta18g01570
CycD Araport: ostta18g01570

CycB Gramene: ostta01g06150
CycB Araport: ostta01g06150

CDKB Gramene: ostta15g00670
CDKB Araport: ostta15g00670

CycA Gramene: ostta02g00150
CycA Araport: ostta02g00150

CDKA Gramene: ostta04g00110
CDKA Araport: ostta04g00110

PsbO Gramene: ostta14g00150
PsbO Araport: ostta14g00150

PsbP Gramene: ostta14g02630
PsbP Araport: ostta14g02630

Fd Gramene: ostta17g00310
Fd Araport: ostta17g00310

APS Gramene: ostta07g03070
APS Araport: ostta07g03070

GBSS Gramene: ostta06g02940
GBSS Araport: ostta06g02940

SBE Gramene: ostta03g00870
SBE Araport: ostta03g00870

SSS Gramene: ostta16g02480
SSS Araport: ostta16g02480

ISA Gramene: ostta02g05060
ISA Araport: ostta02g05060

AMY Gramene: ostta10g00260
AMY Araport: ostta10g00260

LCY Gramene: ostta14g00700
LCY Araport: ostta14g00700

VDE Gramene: ostta16g00660
VDE Araport: ostta16g00660

ZEP Gramene: ostta16g00670
ZEP Araport: ostta16g00670

NRT2 Gramene: ostta10g00950
NRT2 Araport: ostta10g00950

NRT3 Gramene: ostta10g00940
NRT3 Araport: ostta10g00940

NR Gramene: ostta10g00920
NR Araport: ostta10g00920

GS Gramene: ostta01g05020
GS Araport: ostta01g05020

GOGAT Gramene: ostta14g01900
GOGAT Araport: ostta14g01900
