# Supplementary material for: Multiomics integration unveils photoperiodic plasticity in the molecular rhythms of marine phytoplankton
Source: Plant Cell. 2025 Feb 11;37(2):koaf033. doi: 10.1093/plcell/koaf033 (PMC12216367; doi:10.1093/plcell/koaf033)
Supplement: koaf033_Supplementary_Data [file koaf033_Supplementary_Data.zip › supplementary_figures.pdf]

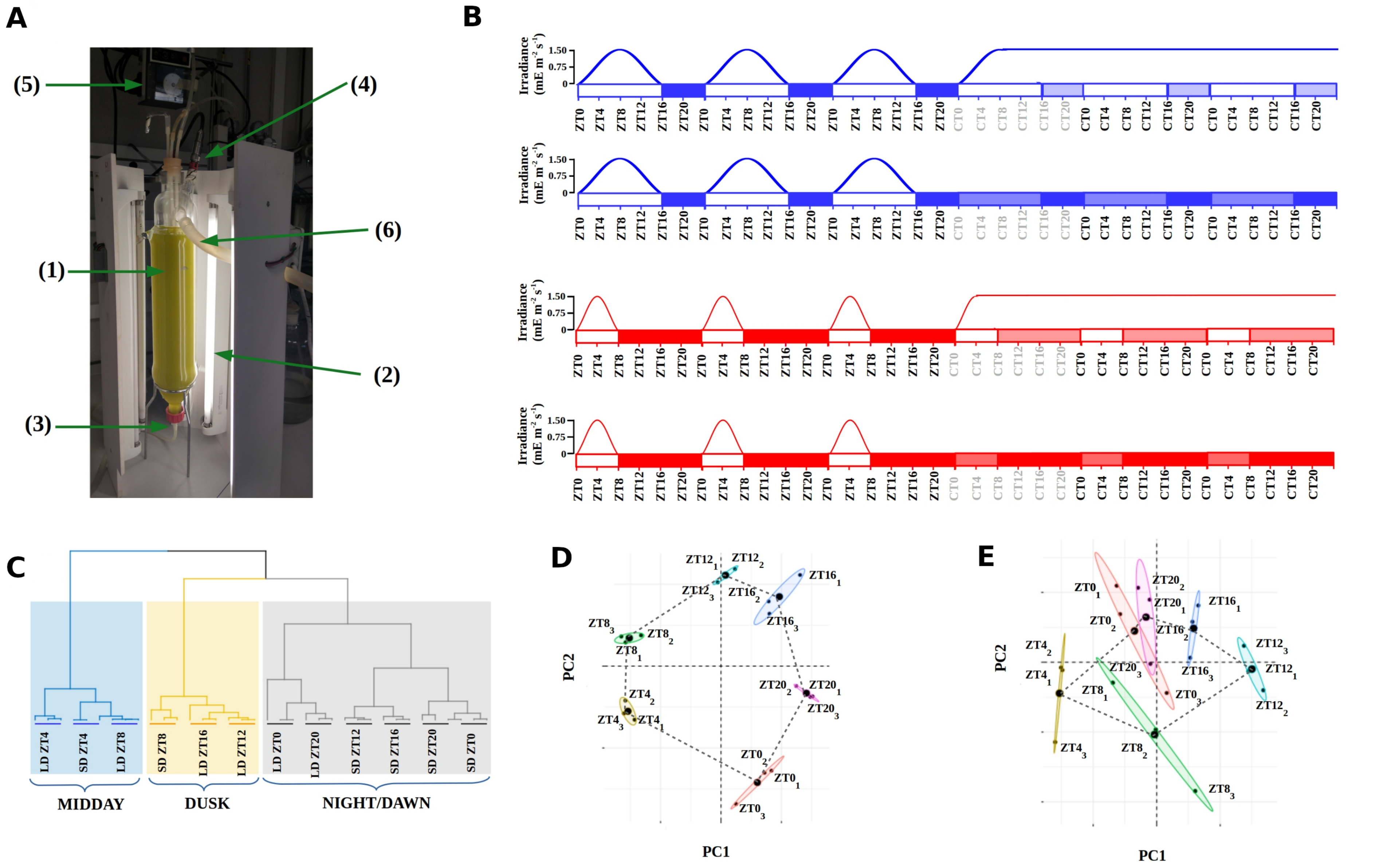
A
(5)
(4)
(6)
(1)
(2)
(3)
B
Irradiance (mE m^{-2} s^{-1})
1.50
0.75
0
ZT0 ZT4 ZT8 ZT12 ZT16 ZT20
CT0 CT4 CT8 CT12 CT16 CT20
C
LD ZT4
SD ZT4
LD ZT8
SD ZT8
LD ZT16
LD ZT12
LD ZT0
LD ZT20
SD ZT12
SD ZT16
SD ZT20
SD ZT0
MIDDAY
DUSK
NIGHT/DAWN
D
PC1
PC2
E
PC1
PC2

Supplementary Figure S1. Experimental design and transcriptomic RNA-seq data reliability. Supports Figure 1. **(A)** Photochemostats used for *Ostreococcus tauri* cultivation in continuous regime under diel and seasonal cycles. (1) Jacketed sterilized bubble column. (2) Light panels simulating the different light/dark cycles. (3) Continuous air is sparged to ensure culture homogenization from the bottom of the column. (4) Temperature and pH probe. (5) Peristaltic pump for fresh medium supply. (6) Overflow tube collecting excess culture. **(B)** The experimental settings to study transcriptome, proteome and physiological rhythmicity: three days under long summer day conditions (LD, 16h light : 8h dark) / short winter day conditions (SD, 8h light : 16h dark) followed by three days under free running conditions consisting of constant light or constant dark. ZTN, Zeitgeber Time N, marks the time point N hours after dawn (lights on, ZT0). Samples were collected every four hours during the three days of alternating light/dark cycles for transcriptomic, proteomic and physiological analysis. No samples were collected during the first day of free running conditions to allow culture acclimation. CTN, Circadian Time N, marks the time point N hours after subjective dawn (CT0). Samples were collected every four hours starting at subjective dawn during two days for transcriptomic analysis. Long day conditions are represented in blue and short day conditions in red. Photoperiods (light periods) correspond to white rectangles and skotoperiods (dark periods) to blue/red filled rectangles. Light blue and light red filled rectangles are used to represent subjective photoperiods and skotoperiods under free running conditions. **(C)** Hierarchical clustering of the RNA-seq data corresponding to the 36 time points collected under alternating dark/light cycles simulating long and short day conditions. The three global transcriptomes corresponding to the same time points from different days cluster together showing robust rhythmicity in our cultures. **(D)** Principal Component Analysis of the time point global transcriptomes under long day conditions. Small dots correspond to the 2D projection of each time point global transcriptome. Big dots correspond to the average of the three replicates 2D projections for each time point. Ellipses mark the 95% confidence regions corresponding to each time point global transcriptome. **(E)** Principal Component Analysis of the time point global transcriptomes under short day conditions. Points and ellipses are used as described before.

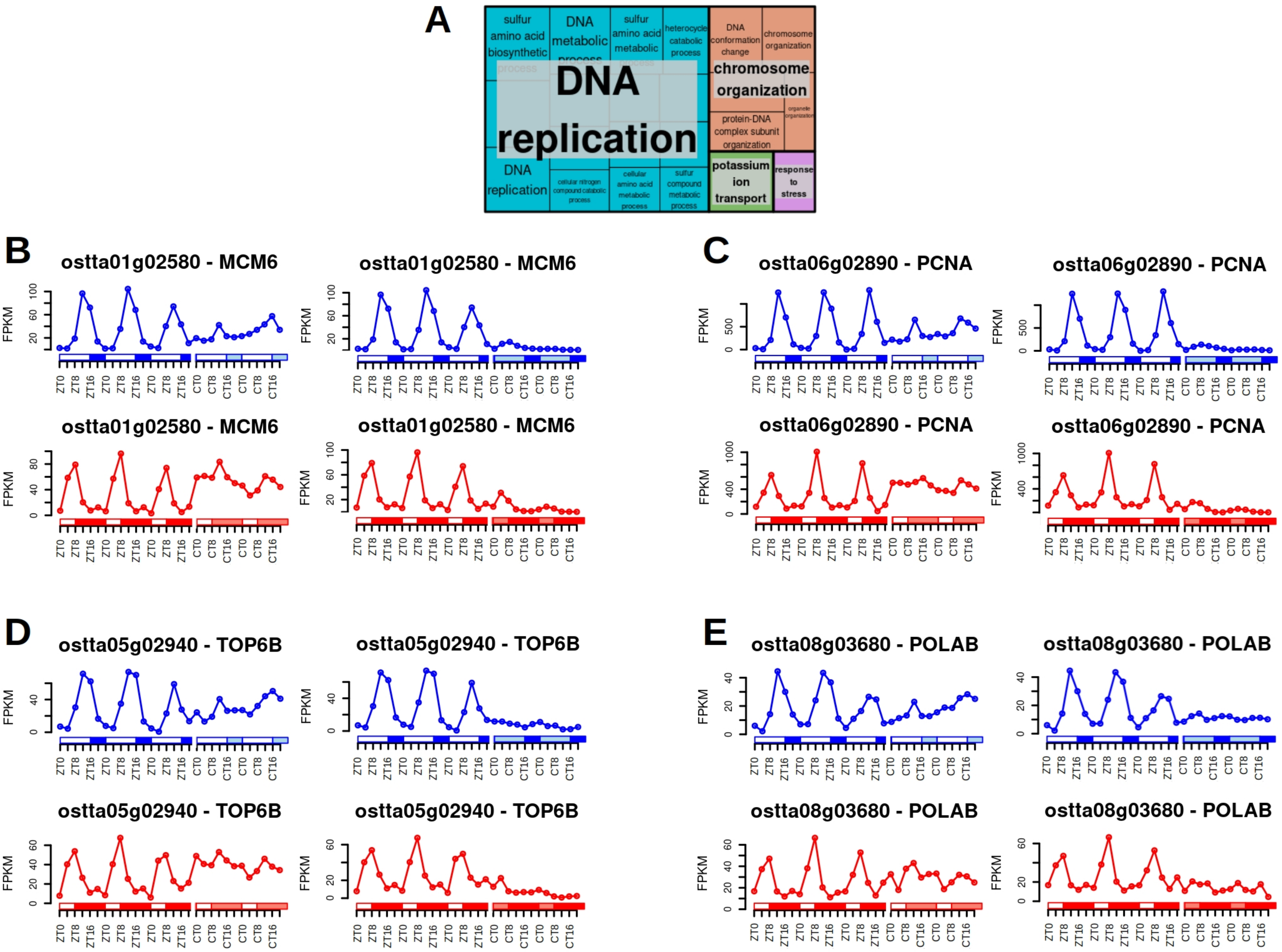
A
sulfur amino acid biosynthetic process
DNA metabolic process
sulfur amino acid metabolic process
heterocycle catabolic process
DNA replication
DNA replication
cellular nitrogen compound catabolic process
cellular amino acid metabolic process
sulfur compound metabolic process
DNA conformation change
chromosome organization
chromosome organization
protein-DNA complex subunit organization
organelle organization
potassium ion transport
response to stress
B
ostta01g02580 - MCM6
ostta01g02580 - MCM6
ostta01g02580 - MCM6
ostta01g02580 - MCM6
C
ostta06g02890 - PCNA
ostta06g02890 - PCNA
ostta06g02890 - PCNA
ostta06g02890 - PCNA
D
ostta05g02940 - TOP6B
ostta05g02940 - TOP6B
ostta05g02940 - TOP6B
ostta05g02940 - TOP6B
E
ostta08g03680 - POLAB
ostta08g03680 - POLAB
ostta08g03680 - POLAB
ostta08g03680 - POLAB
FPKM
ZT0 ZT8 ZT16 ZT0 ZT8 ZT16 ZT0 ZT8 ZT16 CT0 CT8 CT16 CT0 CT8 CT16

Supplementary Figure S2. Biological processes significantly enriched in the genes exhibiting rhythmicity under alternating light/dark cycles and constant light. Supports Figure 2. **(A)** Treemap summarizing the significantly enriched biological processes. Semantically similar biological processes are grouped into the same colored rectangle. The most representative biological process is shown for each rectangle. **(B), (C), (D) and (E)** Gene expression profiles during three consecutive days under long day (LD, 16h light / 8h dark, blue) and two consecutive days under constant light (LL) (top left), LD / constant dark (DD) (top right), short day conditions (SD, 8h light / 16h dark, red) / LL (bottom left) and SD / DD conditions (bottom right) for *Minichromosome Maintenance 6* (*MCM6*, *ostta01g02580*, **B**), *Proliferating Cell Nuclear Antigen* (*PCNA*, *ostta06g02890*, **C**), *Topoisomerase 6 subunit B* (*TOP6B*, *ostta05g02940*, **D**) and *DNA Polymerase Alpha subunit B* (*POLAB*, *ostta08g03680*, **E**). Gene expression levels (measured as FPKM (Fragments Per Kilobase of transcript per Million fragments mapped): White rectangles represent photoperiods (light periods or days), blue and red filled rectangles correspond to skotoperiods under LD and SD respectively (dark periods or nights), light blue rectangles mark subjective nights or days under LL and DD respectively after LD entrainment, and light red rectangles mark subjective nights or days under LL and DD respectively after SD entrainment. ZTN, Zeitgeber Time N, marks the time point N hours after dawn (lights on). CTN, Circadian Time N denotes the time point N hours after the subjective dawn. A discontinuity is shown on the time axis to indicate that samples were collected after 24h acclimation to the corresponding free-running conditions.

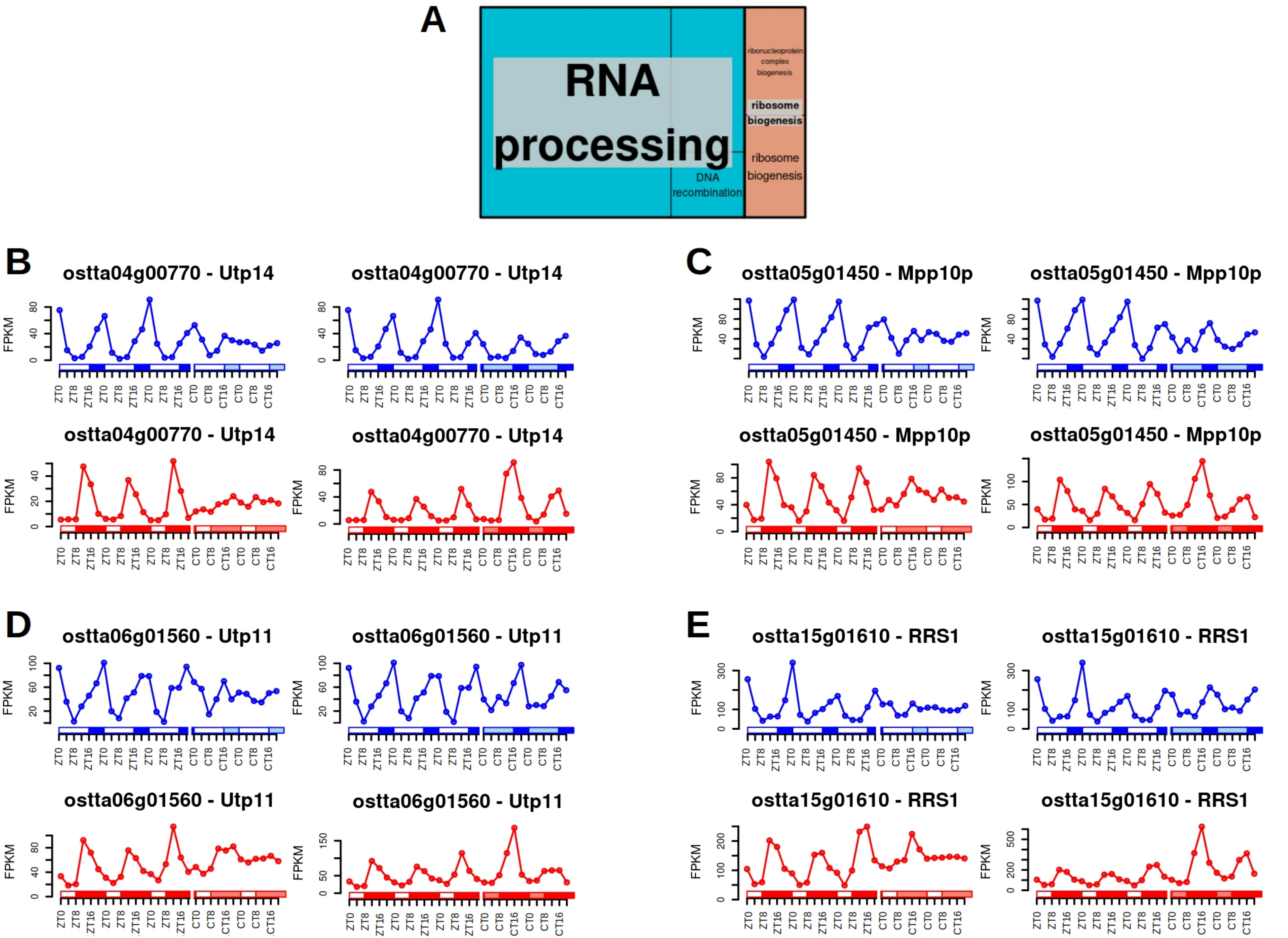

A
RNA processing
DNA recombination
ribonucleoprotein complex biogenesis
ribosome biogenesis
ribosome biogenesis
B
ostta04g00770 - Utp14
C
ostta05g01450 - Mpp10p
D
ostta06g01560 - Utp11
E
ostta15g01610 - RRS1
FPKM
ZT0 ZT8 ZT16
CT0 CT8 CT16

Supplementary Figure S3. Biological processes significantly enriched in the genes exhibiting rhythmicity under alternating light/dark cycles and constant dark. Supports Figure 2. **(A)** Treemap summarizing the significantly enriched biological processes. Semantically similar biological processes are grouped into the same colored rectangle. The most representative biological process is shown for each rectangle. **(B), (C), (D) and (E)** Gene expression profiles during three consecutive days under long day (LD, 16h light / 8h dark, blue) and two consecutive days under constant light (LL) (top left), LD / constant dark (DD) (top right), short day conditions (SD, 8h light / 16h dark, red) / LL (bottom left) and SD / DD conditions (bottom right) for *U3 small nucleolar RNA-associated protein 14* (*Utp14, ostta04g00770,* **B**), *M-phase phosphoprotein 10* (*Mpp10p, ostta05g01450,* **C**), *U3 small nucleolar RNA-associated protein 11* (*Utp11, ostta06g01560,* **D**) and *ribosome biogenesis regulator 1* (*RRS1, ostta15g01610,* **E**). Gene expression levels are measured as FPKM (Fragments Per Kilobase of transcript per Million fragments mapped). White rectangles represent photoperiods (light periods or days), blue and red filled rectangles correspond to skotoperiods under LD and SD respectively (dark periods or nights), light blue rectangles mark subjective nights or days under LL and DD respectively after LD entrainment, and light red rectangles mark subjective nights or days under LL and DD respectively after SD entrainment. ZTN, Zeitgeber Time N, marks the time point N hours after dawn (lights on). CTN, Circadian Time N denotes the time point N hours after the subjective dawn. A discontinuity is shown on the time axis to indicate that samples were collected after 24h acclimation to the corresponding free-running conditions.

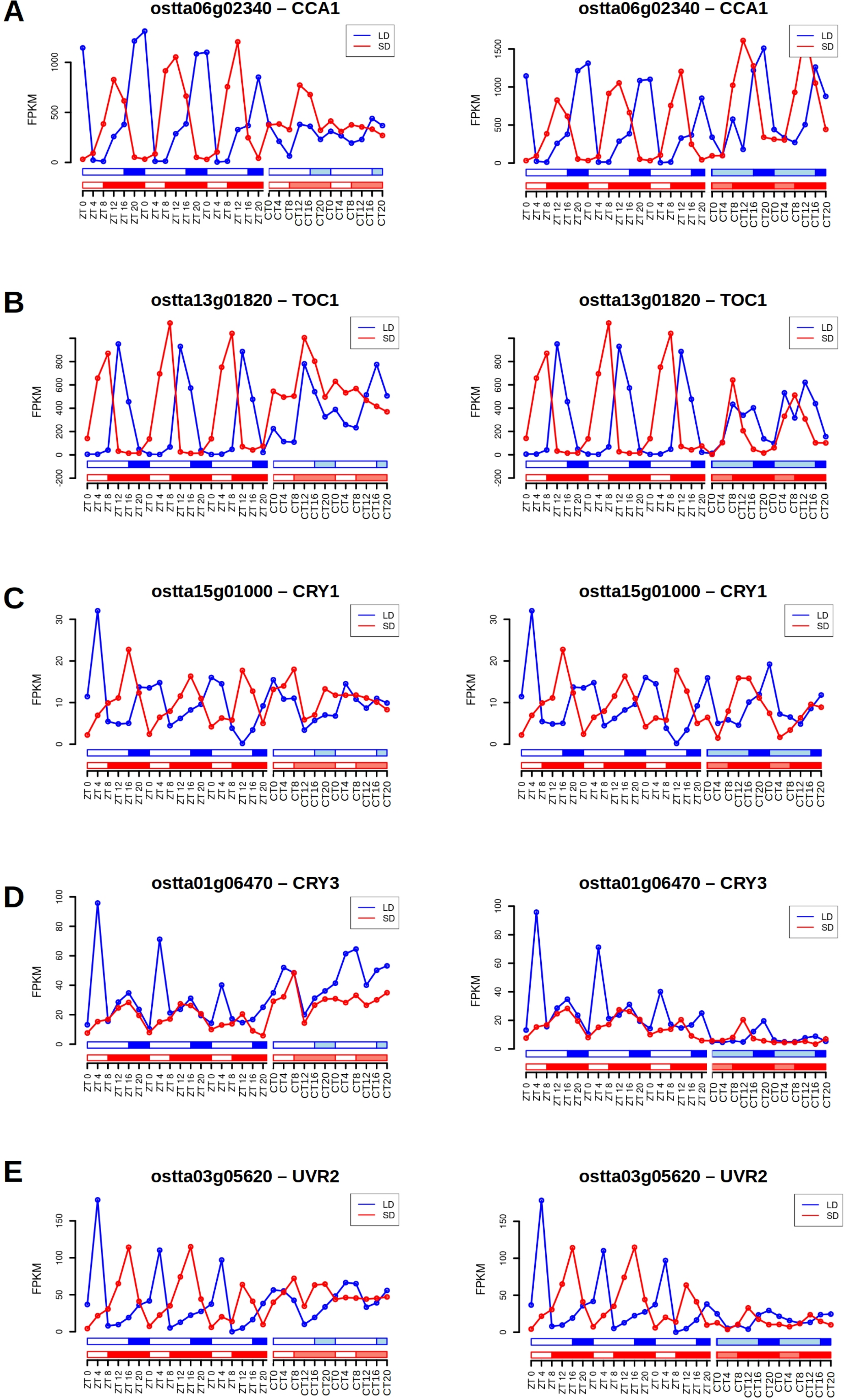
A
ostta06g02340 – CCA1
ostta06g02340 – CCA1
B
ostta13g01820 – TOC1
ostta13g01820 – TOC1
C
ostta15g01000 – CRY1
ostta15g01000 – CRY1
D
ostta01g06470 – CRY3
ostta01g06470 – CRY3
E
ostta03g05620 – UVR2
ostta03g05620 – UVR2
LD
SD
FPKM
ZT 0
ZT 4
ZT 8
ZT 12
ZT 16
ZT 20
CT0
CT4
CT8
CT12
CT16
CT20

Supplementary Figure S4. Gene expression profiles of clock components and light receptors. Supports Figure 2. The gene expression profiles during three consecutive days of clock components and light receptors are presented under long day conditions (LD, 16h light / 8h dark), in blue, and short day conditions (SD, 8h light / 16h dark), in red. Free-running conditions consisting of constant light and dark are also represented during two consecutive days. White rectangles represent photoperiods (light periods or days), blue and red filled rectangles correspond to skotoperiods under LD and SD (dark periods or nights), light blue rectangles mark subjective days or nights under LL and DD respectively after LD entrainment, light red rectangles mark subjective days or nights under LL and DD respectively after SD entrainment. ZTN, Zeitgeber Time N, marks the time point N hours after dawn (lights on). CTN, Circadian Time N, marks the time point N hours after subjective dawn under free-running conditions. A discontinuity is shown on the time axis to indicate that samples were collected after 24h acclimation to the corresponding free-running conditions. **(A)** CIRCADIAN CLOCK ASSOCIATED 1 (CCA1) ortholog exhibits rhythmicity under both LL and DD free-running conditions with higher levels of expression and amplitude under DD. **(B)** TIMING OF CAB EXPRESSION 1 (TOC1) ortholog exhibits rhythmicity under both LL and DD free-running conditions with higher levels of expression under LL. **(C)** CRYPTOCHROME 1 (CRY1) ortholog exhibits rhythmicity under both LL and DD free-running conditions. **(D)** CRYPTOCHROME 3 (CRY3) ortholog exhibits a bimodal rhythmicity specific to LD conditions with only one of these peaks present under SD and free running conditions. This gene was rhythmic under both LL and DD free-running conditions with higher levels of expression under LL. **(E)** UV RESITANCE 2 (UVR2) ortholog exhibits rhythmicity under both LL and DD free-running conditions with higher levels of expression under LL.

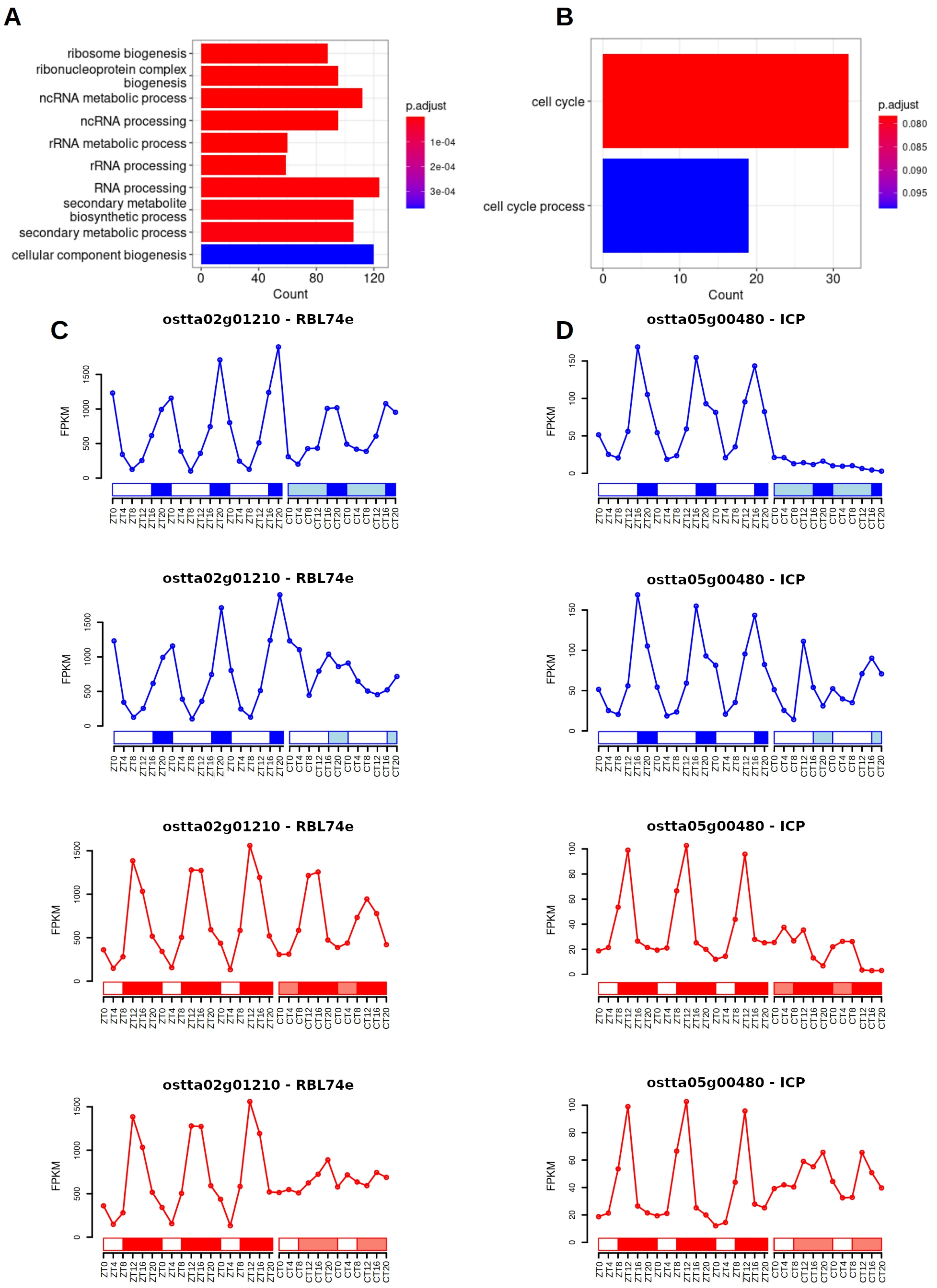
A
ribosome biogenesis
ribonucleoprotein complex biogenesis
ncRNA metabolic process
ncRNA processing
rRNA metabolic process
rRNA processing
RNA processing
secondary metabolite biosynthetic process
secondary metabolic process
cellular component biogenesis
0
40
80
120
Count
p.adjust
1e-04
2e-04
3e-04
B
cell cycle
cell cycle process
0
10
20
30
Count
p.adjust
0.080
0.085
0.090
0.095
C
ostta02g01210 - RBL74e
FPKM
D
ostta05g00480 - ICP
FPKM
ZT0 ZT4 ZT8 ZT12 ZT16 ZT20
CT0 CT4 CT8 CT12 CT16 CT20

Supplementary Figure S5. Functional enrichment over circadian genes after LD and SD entrainment. Supports Figure 2. **(A)** Barplot representing significantly enriched Gene Ontology (GO) terms over the circadian genes identified after LD conditions. Bar lengths represent the number of genes annotated with the corresponding term. Bar colors represent the level of significance. **(B)** Similarly for circadian genes identified after SD conditions. **(C)** Example of circadian gene identified after LD entrainment, *Ribosomal protein L7Ae* (*ostta02g01210*), losing its rhythmicity under constant light after SD entrainment. White rectangles represent photoperiods, blue and red filled rectangles correspond to skotoperiods under LD and SD, light blue rectangles mark subjective days or nights under LL and DD respectively after LD entrainment. Three consecutive days are considered under LD and SD and two under LL and DD. ZTN, Zeitgeber Time N, marks the time point N hours after dawn (lights on). CTN, Circadian Time N, marks the time point N hours after subjective dawn under free-running conditions. A discontinuity is shown on the time axis to indicate that samples were collected after 24h acclimation to the corresponding free-running conditions. **(D)** Example of circadian gene identified after SD entrainment, *Inner centromere protein* (*ostta05g00480*), losing its rhythmicity under constant light after SD entrainment.

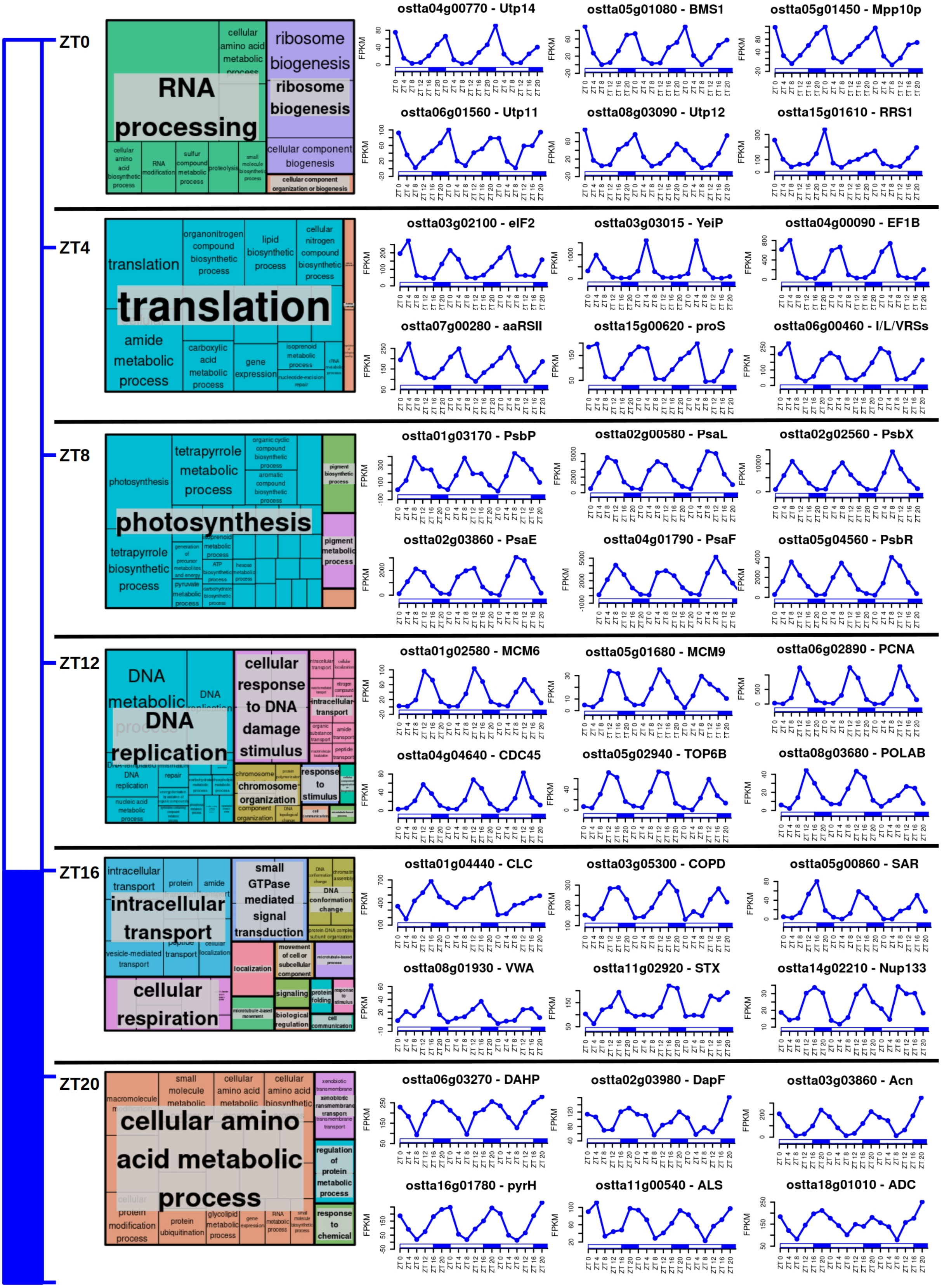
ZT0
RNA processing
ribosome biogenesis
cellular component biogenesis
cellular component organization or biogenesis
cellular amino acid metabolic process
ostta04g00770 - Utp14
ostta05g01080 - BMS1
ostta05g01450 - Mpp10p
ostta06g01560 - Utp11
ostta08g03090 - Utp12
ostta15g01610 - RRS1
ZT4
translation
amide metabolic process
organonitrogen compound biosynthetic process
lipid biosynthetic process
cellular nitrogen compound biosynthetic process
carboxylic acid metabolic process
gene expression
ostta03g02100 - eIF2
ostta03g03015 - YeiP
ostta04g00090 - EF1B
ostta07g00280 - aaRSII
ostta15g00620 - proS
ostta06g00460 - I/L/VRSs
ZT8
photosynthesis
tetrapyrrole metabolic process
tetrapyrrole biosynthetic process
pigment metabolic process
ostta01g03170 - PsbP
ostta02g00580 - PsaL
ostta02g02560 - PsbX
ostta02g03860 - PsaE
ostta04g01790 - PsaF
ostta05g04560 - PsbR
ZT12
DNA replication
DNA metabolic process
cellular response to DNA damage stimulus
intracellular transport
chromosome organization
response to stimulus
ostta01g02580 - MCM6
ostta05g01680 - MCM9
ostta06g02890 - PCNA
ostta04g04640 - CDC45
ostta05g02940 - TOP6B
ostta08g03680 - POLAB
ZT16
intracellular transport
cellular respiration
small GTPase mediated signal transduction
DNA conformation change
localization
ostta01g04440 - CLC
ostta03g05300 - COPD
ostta05g00860 - SAR
ostta08g01930 - VWA
ostta11g02920 - STX
ostta14g02210 - Nup133
ZT20
cellular amino acid metabolic process
regulation of protein metabolic process
response to chemical
protein modification process
protein ubiquitination
ostta06g03270 - DAHP
ostta02g03980 - DapF
ostta03g03860 - Acn
ostta16g01780 - pyrH
ostta11g00540 - ALS
ostta18g01010 - ADC
FPKM

Supplementary Figure S6. Transcriptional temporal program of the distribution of biological processes over diel cycles under long day conditions (16h light / 8h dark). Supports Figure 3. Three consecutive days are represented. The white rectangle represent the photoperiod (light period or day) whereas the blue filled rectangle corresponds to the skotoperiod (dark period or night). ZTN, Zeitgeber Time N, marks the time point N hours after dawn (lights on). Treemaps summarizing the significantly enriched biological processes at each time point. Semantically similar biological processes are grouped into the same colored rectangles. The most representative biological processes are shown for each rectangle. Specific gene expression profiles are represented for each time point illustrating the different biological processes. Gene expression levels are measured as FPKM (Fragments Per Kilobase of transcript per Million fragments mapped). **(ZT0)** RNA processing and ribosome biogenesis are the two most prominent biological processes whose genes reach maximum expression level at dawn under LD conditions. Examples for such genes involved in ribosome biogenesis are *U3 small nucleolar RNA-associated protein 14* (*Utp14, ostta04g00770*), *Ribosome Biogenesis Factor BMS1* (*BMS1, ostta05g01080*), *M-phase phosphoprotein 10* (*Mpp10p, ostta05g01450*), *U3 small nucleolar RNA-associated protein 11* (*Utp11, ostta06g01560*), *U3 small nucleolar RNA-associated protein 12* (*Utp12, ostta08g03090*) and *ribosome biogenesis regulator 1* (*RRS1, ostta15g01610*). **(ZT4)** Translation is the most prominent biological process whose genes reach maximum expression level early in the morning four hours after dawn under LD conditions. Examples for such genes are *eukaryotic Initiation Factor 2* (*eIF2, ostta03g02100*), *translation elongation factor P* (*YeiP, ostta03g03015*), *Elongation Factor 1 B* (*EF1B, ostta04g00090*), *alanyl-tRNA synthase II* (*aaRSII, ostta07g00280*), *proline-tRNA ligase* (*proS, ostta15g00620*) and isoleucyl, leucyl and valyl-tRNA synthetase (*I/L/VRSs, ostta06g00460*). **(ZT8)** Photosynthesis is the most prominent biological process whose genes reach maximum expression level at midday, eight hours after dawn under LD conditions. Examples for such genes are *Photosystem II subunit P* (*PsbP, ostta01g03170*), *Photosystem I subunit L* (*PsaL, ostta02g00580*), *Photosystem II subunit X* (*PsbX, ostta02g02560*), *Photosystem I subunit E* (*PsaE, ostta02g03860*), *Photosystem I subunit F* (*PsaF, ostta04g01790*) and *Photosystem II subunit R* (*PsbR, ostta05g04560*). **(ZT12)** DNA replication and chromosome organization are two prominent biological processes whose genes reach maximum expression level late during the day four hours before dusk under LD conditions. Examples for such genes are *Minichromosome Maintenance 6* (*MCM6, ostta01g02580*), *Minichromosome Maintenance 9* (*MCM9, ostta05g01680*), *Proliferating Cell Nuclear Antigen* (*PCNA, ostta06g02890*), *Cell Division Cycle protein 45* (*CDC45, ostta04g04640*), *Topoisomerase 6 subunit B* (*TOP6B, ostta05g02940*) and *DNA Polymerase Alpha subunit B* (*POLAB, ostta08g03680*). **(ZT16)** Intracellular transport and cellular respiration are the two most prominent biological processes whose genes reach maximum expression level at dusk under LD conditions. Examples for such genes are *Clathrin light chain* (*CLC, ostta01g04440*), *Coatomer delta subunit* (*COPD, ostta03g05300*), *Secretion-associated and Ras-related protein* (*SAR, ostta05g00860*), *von Willebrand factor, type A* (*VWA, ostta08g01930*), *Syntaxin/epimorphin* (*STX, ostta11g02920*), *Nucleoporin 133* (*Nup133, ostta14g02210*). **(ZT20)** Cellular aminoacid metabolic process is the most prominent biological process whose genes reach maximum expression level at midnight four hours before dawn under LD conditions. Examples for such genes are *3-Deoxy-D-arabinoheptulosonate 7-phosphate synthase* (*DAHP, ostta06g03270*), *Diaminopimelate epimerase* (*DapF, ostta02g03980*), *Aconitase/3-isopropylmalate dehydratase* (*Acn, ostta03g03860*), *Uridylate kinase* (*pyrH, ostta16g01780*), *Acetolactate synthase* (*ALS, ostta11g00540*) and *Orn/DAP/Arg decarboxylase* (*ADC, ostta18g01010*).

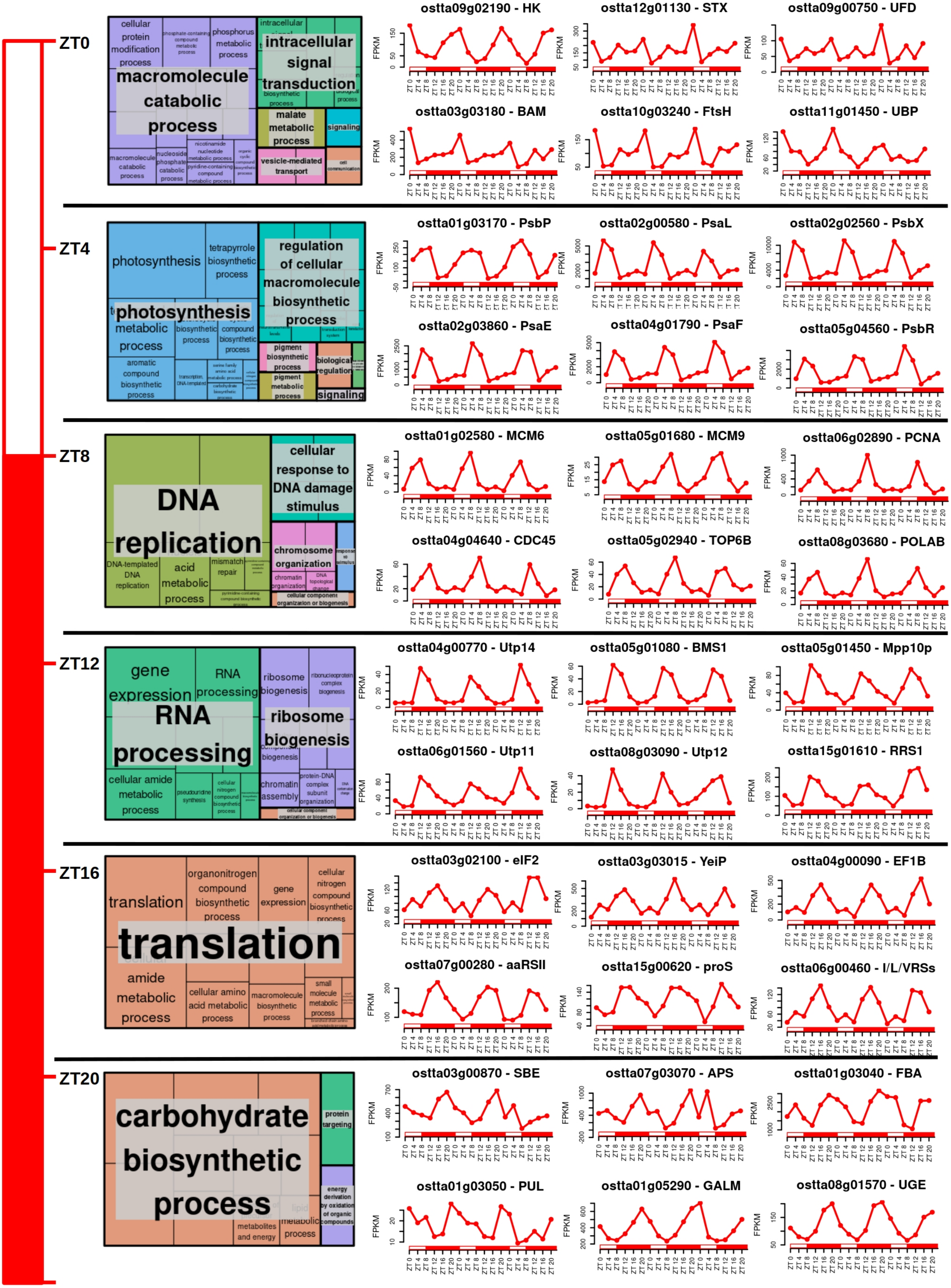
ZT0
macromolecule catabolic process
intracellular signal transduction
cellular protein modification process
phosphorus metabolic process
malate metabolic process
signaling
vesicle-mediated transport
ostta09g02190 - HK
ostta12g01130 - STX
ostta09g00750 - UFD
ostta03g03180 - BAM
ostta10g03240 - FtsH
ostta11g01450 - UBP
ZT4
photosynthesis
photosynthesis
tetrapyrrole biosynthetic process
regulation of cellular macromolecule biosynthetic process
metabolic process
aromatic compound biosynthetic process
pigment biosynthetic process
pigment metabolic process
biological regulation
signaling
ostta01g03170 - PsbP
ostta02g00580 - PsaL
ostta02g02560 - PsbX
ostta02g03860 - PsaE
ostta04g01790 - PsaF
ostta05g04560 - PsbR
ZT8
DNA replication
cellular response to DNA damage stimulus
DNA-templated DNA replication
acid metabolic process
mismatch repair
chromosome organization
cellular component organization or biogenesis
ostta01g02580 - MCM6
ostta05g01680 - MCM9
ostta06g02890 - PCNA
ostta04g04640 - CDC45
ostta05g02940 - TOP6B
ostta08g03680 - POLAB
ZT12
RNA processing
ribosome biogenesis
gene expression
RNA processing
ribosome biogenesis
ribonucleoprotein complex biogenesis
cellular amide metabolic process
pseudouridine synthesis
chromatin assembly
protein-DNA complex subunit organization
ostta04g00770 - Utp14
ostta05g01080 - BMS1
ostta05g01450 - Mpp10p
ostta06g01560 - Utp11
ostta08g03090 - Utp12
ostta15g01610 - RRS1
ZT16
translation
translation
organonitrogen compound biosynthetic process
gene expression
cellular nitrogen compound biosynthetic process
amide metabolic process
cellular amino acid metabolic process
macromolecule biosynthetic process
small molecule metabolic process
ostta03g02100 - eIF2
ostta03g03015 - YeiP
ostta04g00090 - EF1B
ostta07g00280 - aaRSII
ostta15g00620 - proS
ostta06g00460 - I/L/VRSs
ZT20
carbohydrate biosynthetic process
protein targeting
energy derivation by oxidation of organic compounds
metabolites and energy
metabolic process
ostta03g00870 - SBE
ostta07g03070 - APS
ostta01g03040 - FBA
ostta01g03050 - PUL
ostta01g05290 - GALM
ostta08g01570 - UGE
FPKM
ZT 0
ZT 4
ZT 8
ZT 12
ZT 16
ZT 20

Supplementary Figure S7. Transcriptional temporal program of the distribution of biological processes over diel cycles under short day conditions (8h light / 16h dark). Supports Figure 3. Three consecutive days are represented. The white rectangle represent the photoperiod (light period or day) whereas the red filled rectangle corresponds to the skotoperiod (dark period or night). ZTN, Zeitgeber Time N, marks the time point N hours after dawn (lights on). Treemaps summarizing the significantly enriched biological processes at each time point. Semantically similar biological processes are grouped into the same colored rectangles. The most representative biological processes are shown for each rectangle. Specific gene expression profiles are represented for each time point illustrating the different biological processes. Gene expression levels are measured as FPKM (Fragments Per Kilobase of transcript per Million fragments mapped). **(ZT0)** Macromolecule catabolism is the most prominent biological process whose genes reach maximum expression level at dawn under SD conditions. Examples for such genes are *Signal transduction Histidine Kinase* (*HK*, *ostta09g02190*), *Syntaxin/epimorphin* (*STX*, *ostta12g01130*), *Ubiquitin Fusion Degradation protein* (*UFD*, *ostta09g00750*), *Beta amylase* (*BAM*, *ostta03g03180*), *AAA protease FtsH* (*FtsH*, *ostta10g03240*) and *Ubiquitin carboxyl-terminal hydrolase* (*UBP*, *ostta11g01450*). **(ZT4)** Photosynthesis is the most prominent biological process whose genes reach maximum expression level at midday, four hours after dawn under SD conditions. Examples for such genes are *Photosystem II subunit P* (*PsbP*, *ostta01g03170*), *Photosystem I subunit L* (*PsaL*, *ostta02g00580*), *Photosystem II subunit X* (*PsbX*, *ostta02g02560*), *Photosystem I subunit E* (*PsaE*, *ostta02g03860*), *Photosystem I subunit F* (*PsaF*, *ostta04g01790*) and *Photosystem II subunit R* (*PsbR*, *ostta05g04560*). **(ZT8)** DNA replication and chromosome organization are two prominent biological processes whose genes reach maximum expression level at dusk eight hours after dawn under SD conditions. Examples for such genes are *Minichromosome Maintenance 6* (*MCM6*, *ostta01g02580*), *Minichromosome Maintenance 9* (*MCM9*, *ostta05g01680*), *Proliferating Cell Nuclear Antigen* (*PCNA*, *ostta06g02890*), *Cell Division Cycle protein 45* (*CDC45*, *ostta04g04640*), *Topoisomerase 6 subunit B* (*TOP6B*, *ostta05g02940*) and *DNA Polymerase Alpha subunit B* (*POLAB*, *ostta08g03680*). **(ZT12)** RNA processing and ribosome biogenesis are the two most prominent biological processes whose genes reach maximum expression level early during the night four hours after dusk under SD conditions. Examples for such genes involved in ribosome biogenesis are *U3 small nucleolar RNA-associated protein 14* (*Utp14*, *ostta04g00770*), *Ribosome Biogenesis Factor BMS1* (*BMS1*, *ostta05g01080*), *M-phase phosphoprotein 10* (*Mpp10p*, *ostta05g01450*), *U3 small nucleolar RNA-associated protein 11* (*Utp11*, *ostta06g01560*), *U3 small nucleolar RNA-associated protein 12* (*Utp12*, *ostta08g03090*) and *ribosome biogenesis regulator 1* (*RRS1*, *ostta15g01610*). **(ZT16)** Translation is the most prominent biological process whose genes reach maximum expression level during midnight eight hours before dawn under SD conditions. Examples for such genes are *eukaryotic Initiation Factor 2* (*eIF2*, *ostta03g02100*), *translation elongation factor P* (*YeiP*, *ostta03g03015*), *Elongation Factor 1 B* (*EF1B*, *ostta04g00090*), *alanyl-tRNA synthase II* (*aaRSII*, *ostta07g00280*), *proline-tRNA ligase* (*proS*, *ostta15g00620*) and isoleucyl, leucyl and valyl-tRNA synthetase (*I/L/VRSs*, *ostta06g00460*). **(ZT20)** Carbohydrate biosynthesis is the most prominent biological process whose genes reach maximum expression level at the end of the night four hours before dawn under SD conditions. Examples for such genes are *Starch Branching Enzyme* (*SBE*, *ostta03g00870*), *ADP-glucose Pyrophosphorylase Small subunit* (*APS*, *ostta07g03070*), (FBA, ostta01g03040), (PUL, ostta01g03050), (GALM, ostta01g05290) and (UGE, ostta08g01570).

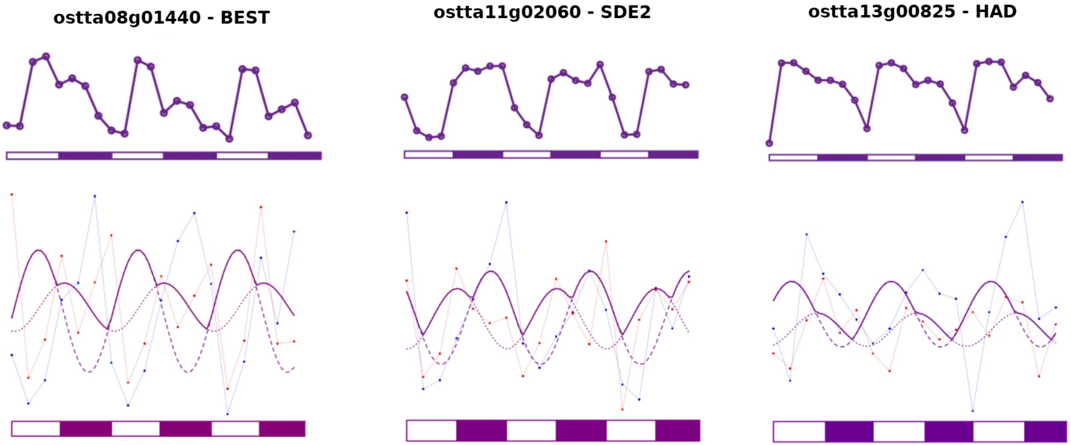

ostta08g01440 - BEST
ostta11g02060 - SDE2
ostta13g00825 - HAD

Supplementary Figure S8. Validation of predicted emergence of bimodal rhythmic profiles under neutral day conditions. Supports Figure 3. Three genes exemplifying the prediction of our model related to the emergence of bimodal expression profiles. Top, microarray data generated under neutral day condition (ND, 12h light / 12h dark) during three consecutive days used to validate our model. Bottom, our model prediction is shown using continuous purple line. Dotted and dashed purple lines represent the two independent waves whose combination produces the observed bimodal rhythmicity. Red and blue line represent the expression profile under short day condition (SD, 8h light / 16h dark) and long day condition (LD, 16h light / 8h dark) during three consecutive days respectively used to generate our predictive model.

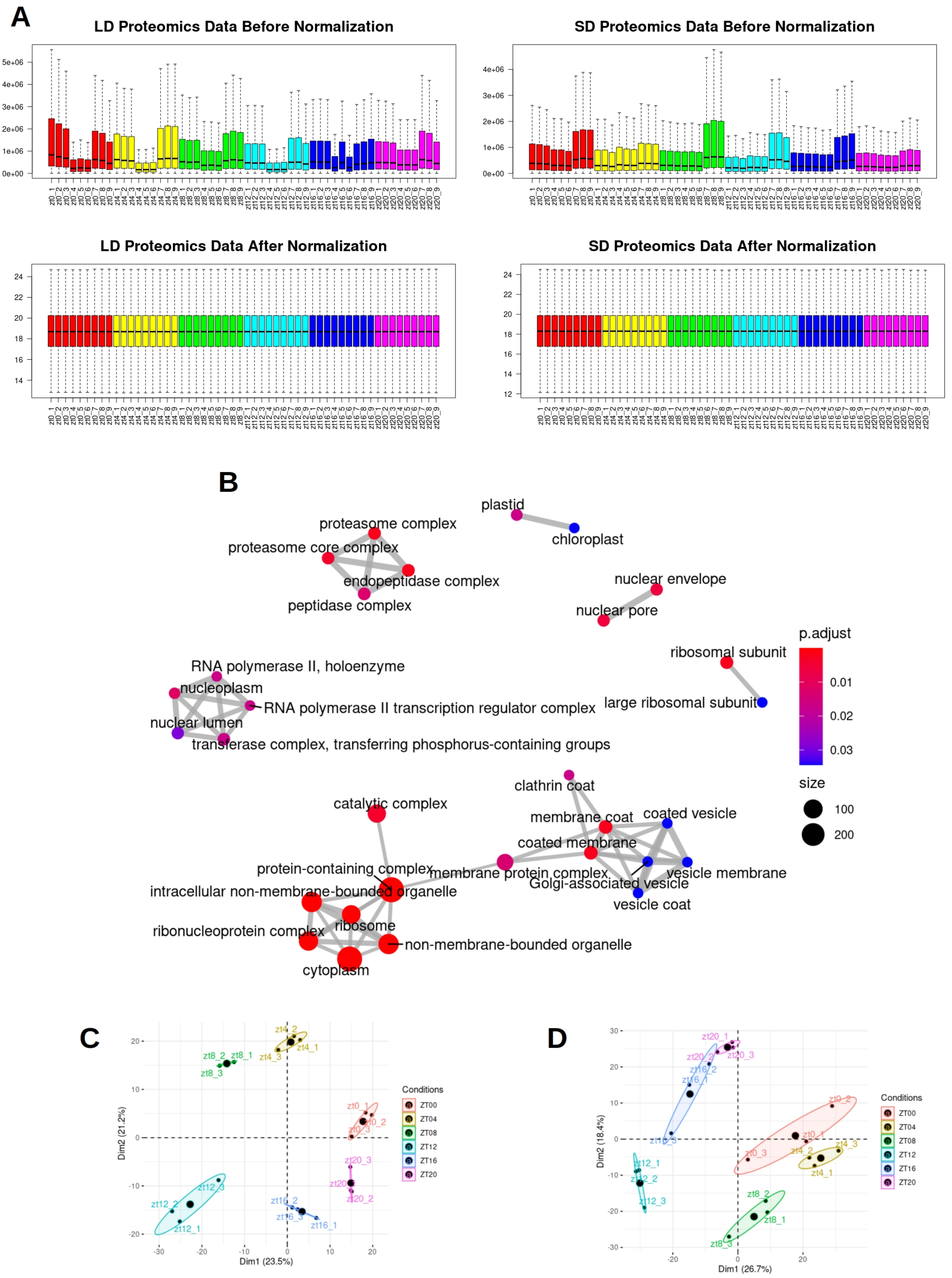
A
LD Proteomics Data Before Normalization
SD Proteomics Data Before Normalization
LD Proteomics Data After Normalization
SD Proteomics Data After Normalization
B
plastid
chloroplast
proteasome complex
proteasome core complex
endopeptidase complex
peptidase complex
nuclear envelope
nuclear pore
ribosomal subunit
large ribosomal subunit
RNA polymerase II, holoenzyme
nucleoplasm
RNA polymerase II transcription regulator complex
nuclear lumen
transferase complex, transferring phosphorus-containing groups
clathrin coat
catalytic complex
membrane coat
coated vesicle
coated membrane
protein-containing complex
membrane protein complex
vesicle membrane
Golgi-associated vesicle
intracellular non-membrane-bounded organelle
vesicle coat
ribonucleoprotein complex
ribosome
non-membrane-bounded organelle
cytoplasm
p.adjust
0.01
0.02
0.03
size
100
200
C
Dim1 (23.5%)
Dim2 (21.2%)
Conditions
ZT00
ZT04
ZT08
ZT12
ZT16
ZT20
D
Dim1 (26.7%)
Dim2 (18.4%)
Conditions
ZT00
ZT04
ZT08
ZT12
ZT16
ZT20

Supplementary Figure S9. Proteomic data normalization and reliability. Supports Figure 4. **(A)** Boxplots representing global distributions of protein abundances over the three technical replicates of the six time points corresponding to three consecutive days under long day conditions (LD, 16h light / 8h dark) on the left and under short day conditions (SD, 8h light / 16h dark) on the right. Top graphs correspond to raw data and bottom graphs correspond to log2 quantile normalized data. Medians are represented by central horizontal lines, upper and lower quartiles by boxes, minimum and maximum values by whisker ends. ZTN, Zeitgeber Time N, marks the time point N hours after dawn (lights on). **(B)** Enrichment map representing the cellular components or organelle significantly covered by the proteins detected in our data. Dots or nodes sizes represent the number of proteins identified located in the corresponding organelle. A blue to red gradient is used to represent the level of significance. Lines or edges link dots or nodes representing related organelle. **(C)** Principal Component Analysis of the time point global rhythmic proteomes under LD conditions. Small dots correspond to the 2D projection of each time point global rhythmic proteome. Big dots correspond to the average of the three replicates 2D projections for each time point. Ellipses mark the 95% confidence regions corresponding to each time point global rhythmic proteome. **(D)** Principal Component Analysis of the time point global rhythmic proteomes under SD conditions. Points and ellipses are used as described before.

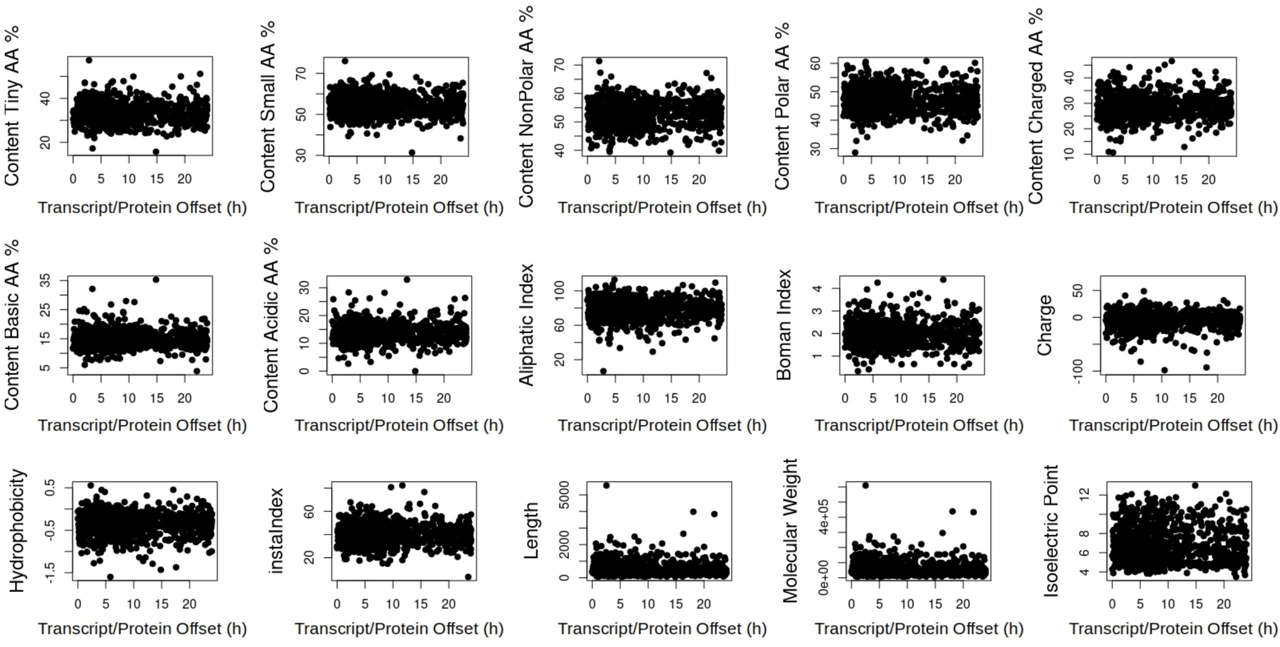

Content Tiny AA %
20
40
Content Small AA %
30
50
70
Content NonPolar AA %
40
50
60
70
Content Polar AA %
30
40
50
60
Content Charged AA %
10
20
30
40
Content Basic AA %
5
15
25
35
Content Acidic AA %
0
10
20
30
Aliphatic Index
20
60
100
Boman Index
1
2
3
4
Charge
-100
0
50
Hydrophobicity
-1.5
-0.5
0.5
instaIndex
20
60
Length
0
2000
5000
Molecular Weight
0e+00
4e+05
Isoelectric Point
4
6
8
12
0
5
10
15
20
Transcript/Protein Offset (h)

Supplementary Figure S10. Transcript/protein phase offsets did not correlate to any biochemical properties computed from protein sequences. Supports Figure 5. Scatter plots where each dot stands for a protein, x-coordinates represent transcript/protein offsets and y-coordinates different protein indexes or properties computed from their amino acid sequences.

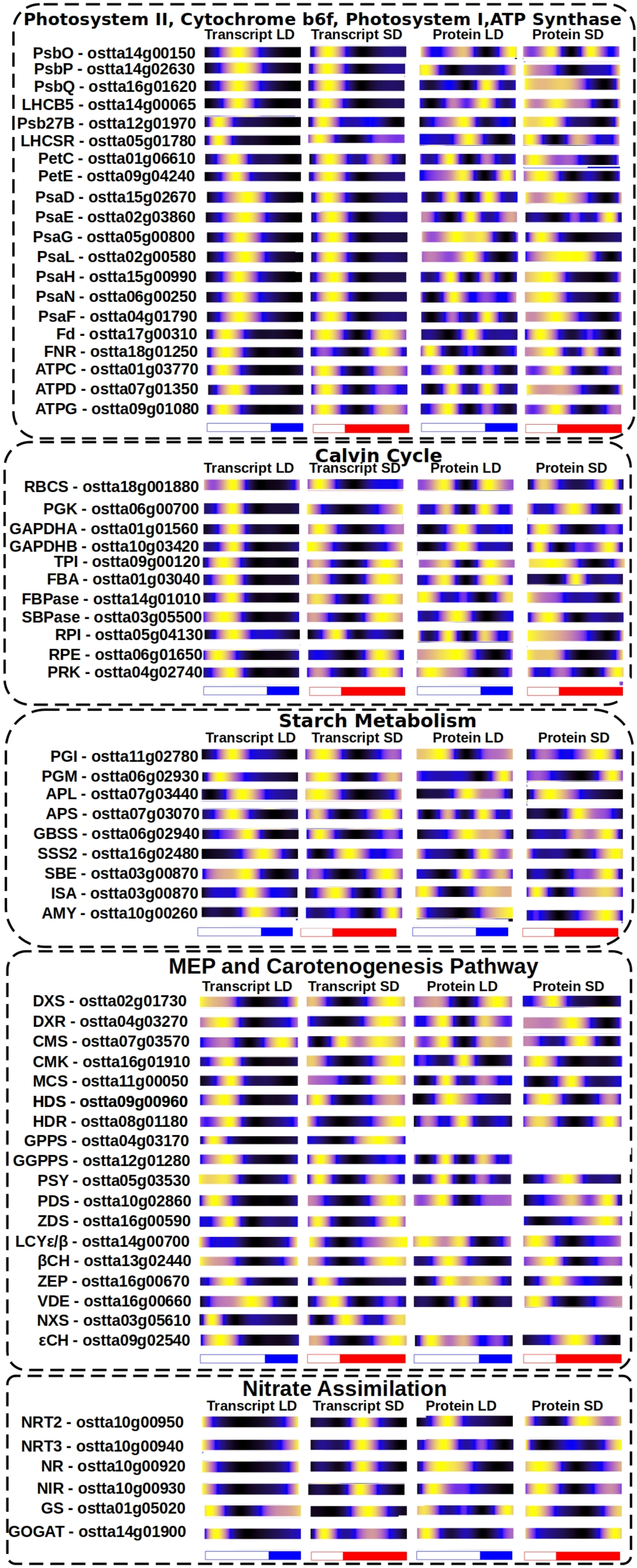
Photosystem II, Cytochrome b6f, Photosystem I,ATP Synthase
Transcript LD
Transcript SD
Protein LD
Protein SD
PsbO - ostta14g00150
PsbP - ostta14g02630
PsbQ - ostta16g01620
LHCB5 - ostta14g00065
Psb27B - ostta12g01970
LHCSR - ostta05g01780
PetC - ostta01g06610
PetE - ostta09g04240
PsaD - ostta15g02670
PsaE - ostta02g03860
PsaG - ostta05g00800
PsaL - ostta02g00580
PsaH - ostta15g00990
PsaN - ostta06g00250
PsaF - ostta04g01790
Fd - ostta17g00310
FNR - ostta18g01250
ATPC - ostta01g03770
ATPD - ostta07g01350
ATPG - ostta09g01080
Calvin Cycle
Transcript LD
Transcript SD
Protein LD
Protein SD
RBCS - ostta18g001880
PGK - ostta06g00700
GAPDHA - ostta01g01560
GAPDHB - ostta10g03420
TPI - ostta09g00120
FBA - ostta01g03040
FBPase - ostta14g01010
SBPase - ostta03g05500
RPI - ostta05g04130
RPE - ostta06g01650
PRK - ostta04g02740
Starch Metabolism
Transcript LD
Transcript SD
Protein LD
Protein SD
PGI - ostta11g02780
PGM - ostta06g02930
APL - ostta07g03440
APS - ostta07g03070
GBSS - ostta06g02940
SSS2 - ostta16g02480
SBE - ostta03g00870
ISA - ostta03g00870
AMY - ostta10g00260
MEP and Carotenogenesis Pathway
Transcript LD
Transcript SD
Protein LD
Protein SD
DXS - ostta02g01730
DXR - ostta04g03270
CMS - ostta07g03570
CMK - ostta16g01910
MCS - ostta11g00050
HDS - ostta09g00960
HDR - ostta08g01180
GPPS - ostta04g03170
GGPPS - ostta12g01280
PSY - ostta05g03530
PDS - ostta10g02860
ZDS - ostta16g00590
LCYε/β - ostta14g00700
βCH - ostta13g02440
ZEP - ostta16g00670
VDE - ostta16g00660
NXS - ostta03g05610
εCH - ostta09g02540
Nitrate Assimilation
Transcript LD
Transcript SD
Protein LD
Protein SD
NRT2 - ostta10g00950
NRT3 - ostta10g00940
NR - ostta10g00920
NIR - ostta10g00930
GS - ostta01g05020
GOGAT - ostta14g01900

Supplementary Figure S11. Heatmaps integrating transcript and protein abundance under LD and SD conditions for differente biological processes. Supports Figures 7, 8, 9 and 10. For each enzyme its corresponding protein and transcript abundances under LD, in blue, and SD conditions, in red, are represented using heatmaps. Black represents low, blue medium and yellow high abundances. White rectangles represent photoperiods, blue and red filled rectangles correspond to skotoperiods under LD and SD, respectively.

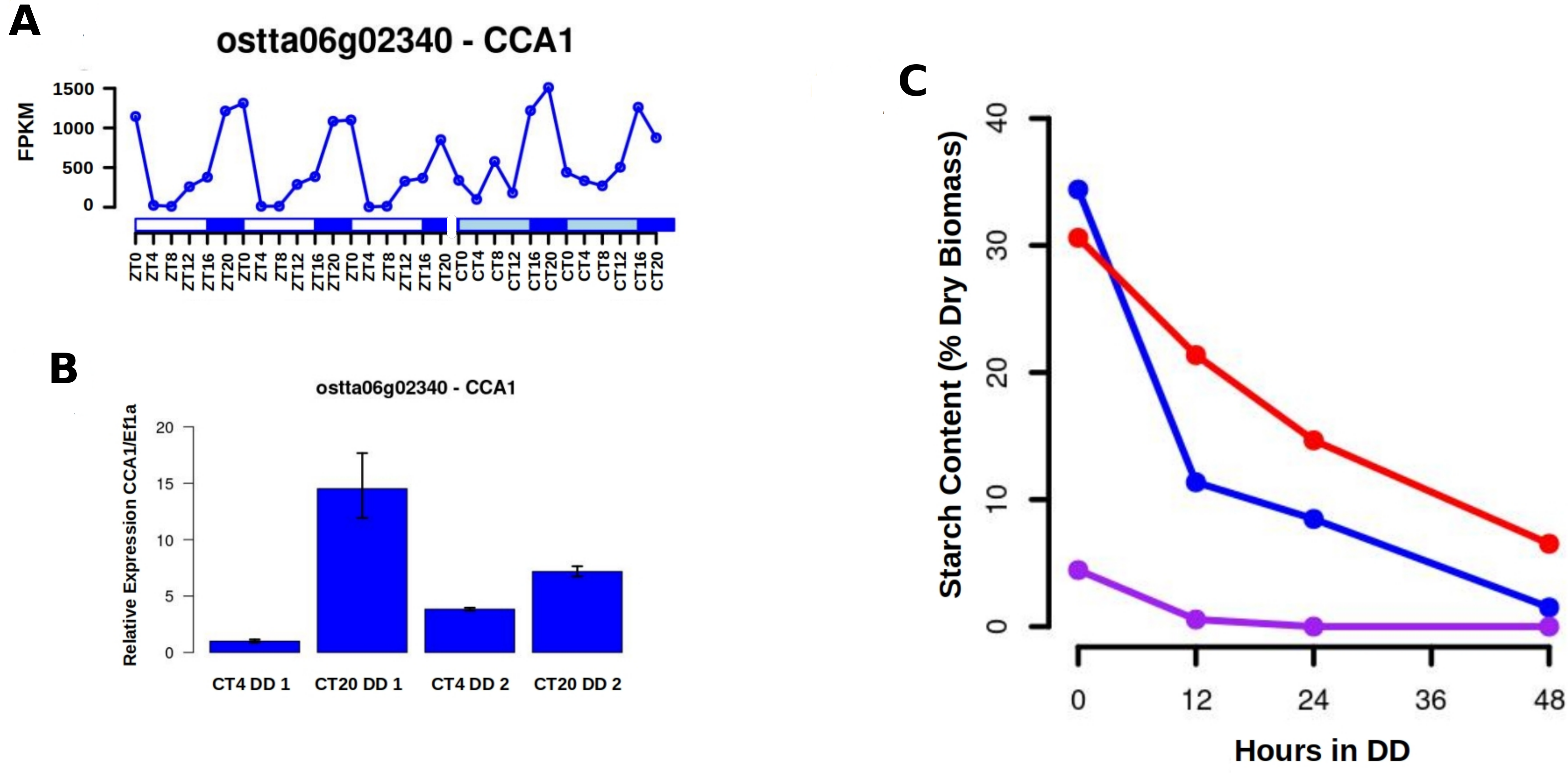
A
ostta06g02340 - CCA1
FPKM
1500
1000
500
0
ZT0 ZT4 ZT8 ZT12 ZT16 ZT20 ZT0 ZT4 ZT8 ZT12 ZT16 ZT20 ZT0 ZT4 ZT8 ZT12 ZT16 ZT20 CT0 CT4 CT8 CT12 CT16 CT20 CT0 CT4 CT8 CT12 CT16 CT20
B
ostta06g02340 - CCA1
Relative Expression CCA1/Ef1a
20
15
10
5
0
CT4 DD 1
CT20 DD 1
CT4 DD 2
CT20 DD 2
C
Starch Content (% Dry Biomass)
40
30
20
10
0
0
12
24
36
48
Hours in DD

Supplementary Figure S12. Gene expression and Starch content under constant dark. Supports Figure 8. **(A)** RNA-seq gene expression profile during three consecutive days under long day (LD, 16h light / 8h dark) and two consecutive days under constant dark (DD) of Ostreococcus CCA1 ortholog (ostta06g02340). White rectangles represent photoperiods (light periods or days), blue filled rectangles correspond to skotoperiods (dark periods or nights) under LD, light blue rectangles mark subjective days under DD. ZTN, Zeitgeber Time N, marks the time point N hours after dawn (lights on, ZT0). CTN, Circadian Time N, denotes the time point N hours after subjective dawn. A discontinuity is shown on the time axis to indicate that samples were collected after 24h acclimation to the corresponding free-running conditions. **(B)** CCA1 RT-qPCR gene expression estimates relative to Ef1a (Elongation factor 1a) at CT4 and CT20 for two consecutive days under DD. Vertical lines represent standard errors. **(C)** Starch content profile measured during two consecutive days under DD. Blue and red lines represent starch content in LD and SD entrained cultures grown in continuous mode with CO2 injection on demand and high nitrate medium. Purple line represents starch content in neutral day condition (12h light / 12h dark) entrained cultures grown in batch mode without aeration and low nitrate medium.

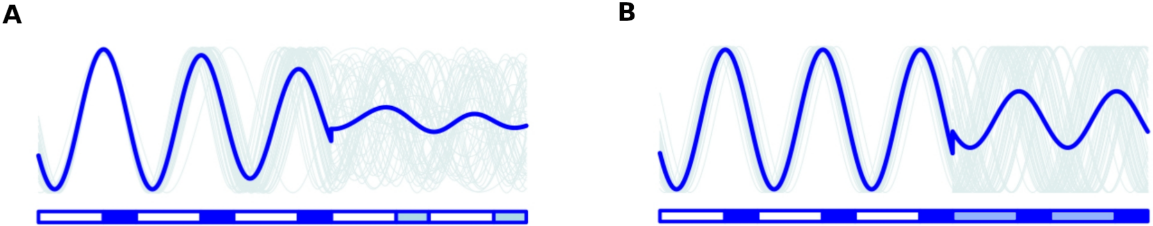
A
B

Supplementary Figure S13. Reductions in amplitude under free running conditions can be explained by a decline in culture synchrony. Supports Figure 2. **(A)** Culture average gene expression profile during three consecutive days under long day (LD, 16h light / 8h dark) and during two days under constant light (LL) is represented by a thick blue line. Examples of individual cell gene expression profiles under LD and LL are represented by thin grey lines. White rectangles represent photoperiods (light periods or days), blue filled rectangles correspond to skotoperiods (dark periods or nights) and light blue filled rectangles stand for subjective nights under LL free running conditions. When cultures are transferred to LL, cells get desynchronized and their individual gene expression profiles become largely out of phase. This results in a drastic reduction in the amplitude of the culture average gene expression profile although individual gene expression profiles maintain the same amplitude. **(B)** Culture average gene expression profile under LD and constant dark (DD) is represented by a thick blue line. Examples of individual cell gene expression profiles under LD and DD are represented by thin grey lines. When cultures are transferred to DD, cells get mildly desynchronized and their individual gene expression profiles become moderately out of phase. This results in a slight reduction in the amplitude of the culture average gene expression profile although individual gene expression profiles maintain the same amplitude.
