# Supplementary material for: Multiomics integration unveils photoperiodic plasticity in the molecular rhythms of marine phytoplankton
Source: Plant Cell. 2025 Feb 11;37(2):koaf033. doi: 10.1093/plcell/koaf033 (PMC12216367; doi:10.1093/plcell/koaf033)
Supplement: koaf033_Supplementary_Data [file tpc.00564.2024-s17.pdf › tpc.00564.2024-s17.pdf]

THE PLANT CELL

Unveiling Photoperiodic Plasticity in Molecular Rhythms in Marine Phytoplankton through Multiomics Integration

Ana Romero-Losada, Christina Arvanitidou, Elena García-Gómez, María Morales-Pineda, María Castro-Pérez, Yen Chew, Gerben van Ooijen, Mercedes García-González, and Francisco Romero-Campero

Review Timeline:

Submission Date:	12-Jan-2024
Editorial Decision:	23-Jan-2024
Resubmission Received:	07-Jun-2024
Editorial Decision:	08-Jul-2024
Revision Received:	08-Oct-2024
Editorial Decision:	15-Nov-2024
Revision Received:	17-Dec-2024
Accepted:	22-Dec-2024

1st Editorial Decision 23-Jan-2024

Dear Dr. Romero-Campero:

Thank you for submitting your manuscript entitled "Unveiling Seasonal Adaptive Molecular Rhythms in Marine Phytoplankton during Diel Cycles through Multiomics Integration" to The Plant Cell. Your submission has been evaluated by members of the editorial board, and we regret to inform you that we are not recommending that your manuscript in its current form proceed further in the review process.

We feel it best to not send your paper out for review until you have had sufficient time to complete this work. However, we encourage you to resubmit a revised version of your manuscript to The Plant Cell.

This article contains a large number of new data and results that should be of interest to the wide community of photobiologists and chronobiologists. However, there is a conundrum with respect to prior literature that needs to be addressed. as reviewers will have difficulties without some more information and/or experiments. This work reports the existence of a transcriptional oscillator in Ostreococcus active under conditions of continuous light and darkness. However, work by O'Neill et al. show that in Ostreococcus the transcriptional clock does not function in the dark, demonstrated using a number of lines of evidence (doi: 10.1038/nature09654). As such, these two reports seem to be discordant. We feel it it will be important to verify and describe the expression of CCA1 and TOC1 to provide reason or evidence for this discordance. This will be important to successfully navigating the review process.

If you can attend to these items we will be happy to reconsider the manuscript. We thank you for your interest in and support of The Plant Cell. We look forward to your next submission and hope we will be able to render a more positive decision on future work.

On behalf of the editorial board,

Blake Meyers, Editor-in-Chief

The Plant Cell

Instituto de Bioquímica Vegetal y Fotosíntesis
(Consejo Superior de Investigaciones Científicas-
Universidad de Sevilla)

Dear Editor,

Please find enclosed our manuscript entitled **"Unveiling Seasonal Adaptive Molecular Rhythms in Marine Phytoplankton during Diel Cycles through Multiomics Integration"** to be reconsidered for publication in *The Plant Cell*.

In our previous submission we described rhythmicity in gene expression under constant dark (DD) in *Ostreococcus tauri* which was contradictory with previously published results by (O'Neill et al., 2011). We thank the editor for pointing out this discrepancy that has helped us to deepen our studies in *Ostreococcus*. This has allowed us to better explain the effect of DD free-running conditions and confirm the suitability of our experimental design for studying rhythmicity in *Ostreococcus* while removing potential nutritional stresses.

Specifically, to solve this apparent conundrum we have established a collaboration with Gerben van Ooijen one of the authors in (O'Neill et al., 2011) and performed some experiments together and interpreted the results. Indeed, two new authors has been included in our paper, Gerben van Ooijen and Yen Peng Chew. We found that this apparent discrepancy can be attributed to the significant disparities in growth conditions between our experiments and those previously published. Our cultures are maintained in continuous mode, with a steady supply of fresh, high nitrate medium and CO2 injection to regulate pH levels. In contrast, in previously published studies, cells are grown in batch mode without aeration and low nitrate. Under our growth conditions, *Ostreococcus* cells accumulate substantial amounts of starch, comprising 30% of dry weight. These starch reserves are gradually consumed under DD maintaining high levels after 48h, indicating continued metabolic activity in *Ostreococcus* cells, see Figure below.

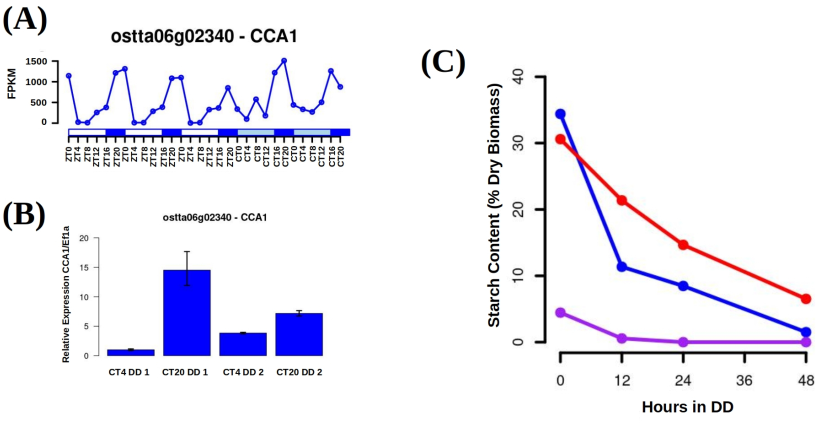

Gene expression and Starch content under DD. (A) RNA-seq gene expression profile under long day (LD, 16h light / 8h dark) and constant dark (DD) of Ostreococcus CCA1 ortholog. White rectangles represent photoperiods, blue filled rectangles correspond to skotoperiods, light blue rectangles mark subjective days under DD. ZTN, Zeitgeber Time N, marks the time point N hours after dawn (lights on, ZT0). CTN, Circadian Time N, denotes the time point N hours after subjective dawn. **(B)** CCA1 RT-qPCR gene expression estimates relative to Ef1a (Elongation factor 1a) at CT4 and CT20 for two consecutive days under DD. **(C)** Starch content profile measured during two consecutive days under DD. Blue and red lines represent starch content in LD and SD entrained cultures grown in continuous mode with CO2 injection on demand and high nitrate medium. Purple line represents starch content in neutral day condition (12h light / 12h dark) entrained cultures grown in batch mode without aeration and low nitrate medium.

The energy derived from starch catabolism likely prevents nutritional stress, thereby enabling the clock to function under DD. Conversely, in batch cultures without aeration and with low nitrate medium, *Ostreococcus* cells synthesize starch constituting around 5% of DW during the light period, which is nearly entirely consumed during the subsequent 12h night. Prolonged dark periods would likely induce nutritional stress halting the oscillations of clock components. Therefore, arrhythmicity under dark conditions would not be a direct response to darkness but rather a consequence of nutritional stress. In contrast, growth conditions used for this study mitigate this nutritional stress, enabling the study of rhythmicity under free-running conditions in DD.

We believe that these new experiments and analysis clearly explains why we observed active transcription under DD and demonstrate the suitability of our experimental design for studying rhythmicity in *Ostreococcus* while removing potential nutritional stresses. Therefore, we hope our work can now proceed to the reviewers for evaluation and consideration for publication in The Plant Cell.

Yours sincerely,

Francisco J. Romero-Campero   Mercedes García-González

Dr. Francisco J. Romero-Campero
Universidad de Sevilla
Computer Science and Artificial Intelligence
Sevilla, Sevilla
Spain

Dear Dr. Romero-Campero:

We have received reviews of your manuscript entitled "Unveiling Seasonal Adaptive Molecular Rhythms in Marine Phytoplankton during Diel Cycles through Multiomics Integration." Thank you for submitting your best work to The Plant Cell. The editorial board agrees that the work you describe is substantive, falls within the scope of the journal, and may become acceptable for publication, pending revision and potential re-review.

We ask you to pay attention to the following points in preparing your revision:

All reviewers agree that your work provides a quality dataset, which will be useful in advancing our understanding of photoperiod regulation in algae and plants. However, the reviewers also expressed some concerns about the descriptive nature of the work and made some useful suggestions to improve it.

More specifically, the reviewers consider that the paper lacks critical comparisons with similar studies already carried out in other systems. After consultation of the reviewers with the editorial board, we recommend extending the computational analysis of your data to identify conserved or possibly novel regulators involved in photoperiodic regulation in Ostreoccoccus. If possible, on the basis of the genomic information available in Ostreococcus, the authors could also consider analyzing potential cis-regulatory elements in the promoter regions of the rhythmic genes. When compared to results from land plants, this analysis could provide some potential regulatory insight. Any effort at a comparative analysis, bioinformatic and editorial, will help the general audience of the journal grab onto the manuscript and strengthen its impact.

Reviewers also made suggestion to improve the presentation of your results.

Please contact us if there are ambiguous comments or if you wish to discuss the revision.

A note from the Editor-in-Chief: We are trying to make a concerted effort to change green/red comparisons to green/magenta to make our figures understandable to those with color vision deficiencies. We noticed that a small number of your figures utilize red/green contrasts, so as you prepare the final version of the figures, please check the figures for red/green color use. Magenta is a good substitute for red, and a light blue substitutes for green even if the data are labeled as "RFP" or "mCherry" - readers will understand that colors can be changed, and indeed, it can be adjusted relatively quickly by using the "hue" setting in Photoshop or similar software. For example, the following figures may need to be corrected: Figure 6H; you might want to check over the Supplemental Figures for similar comparisons. Please note that color changes do not need to be highlighted or tracked in the revised manuscript, but could be noted in the cover letter or response document.

Also, I noticed that your figure panel labels utilize an oversized serif font (Times, I think) in parentheses, and the journal requirement generally is a sans serif font such as Arial. More specifically, we don't use parenthesis, and the panel marker is just a bold capital letter; we can also fix this later, but it might save some work to do it now. Please check your supplemental figures too.

Given the nature of the comments, we are offering you 60 days from when we have issued this decision to complete the revision. If a revision is not returned within this time frame, and if you have not been granted an extension, we will withdraw the manuscript, which will leave you free to submit the work elsewhere. If you need an extension, we encourage you to contact us at any point before the 60 days have passed.

When you are ready to submit the revised version, please upload a highlighted copy that indicates all changes made in response to the editor and reviewer recommendations. Include an itemized list of all changes made in response to each of the reviewer's suggestions in the "Response to Reviewers" section; please note that reviewers do not have access to your cover letter, nor was this decision letter shared with them.

Thank you for the privilege of reviewing your work. We look forward to receiving your revised manuscript.

On behalf of the editorial board,

Reviewing Editor, Board of Reviewing Editors
Daniel Kliebenstein, Senior Editor
Blake Meyers, Editor-in-Chief

The Plant Cell

Please note the following:

-The Plant Cell now requires authors to complete and submit an author revisions checklist upon submission of a revised manuscript. The aim of the checklist is to aid authors in preparing a high-quality manuscript, facilitate the review and assessment of revised manuscripts, and help to ensure that journal standards are maintained across the board. If your manuscript is accepted, the completed checklist will be published as supplemental material attached to the article online. Please download a copy of the checklist (pdf fillable form) at this link, for submission with your revised manuscript: https://tpc.msubmit.net/html/Author_Revisions_Checklist.pdf.

-Supplemental materials should be restricted to large datasets and tables, presentation of replicates, and validation of reagents, methods, or genotypes. Any data that are used to support the major claims must be in the main manuscript. Supplemental figure legends must indicate what figure in the main manuscript is supported by the supplemental data presented. Please justify how each of the supplemental figures meet the criteria.

-Sampling methods and nature of "biological replicates" should be described precisely (i.e. different plants, parts of plants, pooled tissue, independent pools of tissue, sampled at different times, etc), along with a clear description of and rationale for any statistical analyses conducted. The reader should know exactly what was sampled; what forms the basis of the calculation of any means and statistical parameters reported. This is also necessary to ensure that proper statistical analysis was conducted.

-Want to add this revision deadline to your calendar? Click below!

-- Reviewer comments:

Reviewer #1 (Comments for the Author):

This article describes the generation and analysis of an extensive transcriptomic, proteomic, and physiological dataset profiling diel cycles in the picoeukaryote Ostreococcus tauri. The availability of different omics layers allowing studying specific responses, their (dis)agreements or temporal shifts at the transcript and protein level, and the effect diel cycles have on different gene functions and pathways.
Overall, the manuscript is well written and easy to follow. The authors wrote a good Introduction which does a great job on explaining key concepts (but see comment 1). While the study is overall sound, I am not fully convinced of the ground-braking nature and novelty of this manuscript. While the generated dataset is unique and present a great resource for the research community, the Results section is very descriptive. A missed opportunity is that no/limited new insights are reported about e.g. the mechanisms of the underlying gene regulation (transcriptional and/or at the protein level). Furthermore, as similar datasets exist in related unicellular/green eukaryotes (e.g. Kingdom-wide comparison reveals the evolution of diurnal gene expression in Archaeplastida ; https://www.nature.com/articles/s41467-019-08703-2), an in-depth cross-species analysis has the potential to uncover new findings about e.g. conserved genes or functional features, which could generate new testable hypotheses.

Major
1. Introduction: while the different terms and problem statement is clearly defined, I am missing information on what previous papers have reported on the molecular analysis of diel rhythms (lines 56-57 and 73-75 remain vague). Please add a few sentences explaining the key findings of these papers, which will give more context to the newly presented results.
2. While information on replicates is available for the proteome profiling, information on replicates is missing for the RNA-Seq transcript profiling (unless I missed it).
3. Lines 205-... The observation of two peaks per day (bimodal rhythmic expression) is interesting and reminds my of a similar paper published in diatoms some years ago (https://pubmed.ncbi.nlm.nih.gov/33901335/). While the authors here briefly cite two papers in plants and animals (line 585), extending this analysis, and/or the discussion, would allow to put these results in a broader context and could confirm/refute hypotheses formulated about the biological significance of bimodal rhythmic expression.
4. Methods: for both the RAIN and CircaCompare rhythmic analyses, it is mentioned that a "p-value threshold was used". What kind of multiple testing correction was performed for these analyses, to correct for significance testing of a large number of genes, or proteins, or physiological parameters? Please explain the multiple-testing strategy clearly in the Methods.
5. Methods: RAIN was used to determine the significance of rhythmicity, while circaCompare was used to compare rhythmic parameters (phase, amplitude etc). Why not use only circaCompare for everything since it is also able to determine the significance of rhythmicity? Explain the rationale.

6. Line 159: it is mentioned that a transfer to DD would increase the amplitude, but this is not shown in the figure 2 D (in other panels, a significant change is shown by the p-value). Are you sure it was significant?
7. Line 159: I wonder if only the amplitudes of the significantly rhythmic genes are being considered here? Because the number of rhythmic genes is much lower in DD (post SD) compared to SD, you would expect that the average amplitude should drop considerably. If it is true that you only consider the amplitudes of the significantly rhythmic genes, this should be stated more clear, as it might simply reflect that only the genes with the highest amplitudes are still rhythmic in DD.
8. Figure 5, panel C: this is not the best visualization as the GO identifiers are very small/hard to read and nobody knowns these identifiers by hard. Therefore, add GO description next to all or a selection of relevant term and consider removing some terms (e.g. those that are redundant with selected terms or those that are not discussed in the text).
Minor
9. Line 164: Please explain what "advanced phases" are
10. Line 266: Revise the title of this section: Combining proteome and ... to
11. Line 578: "dependent" instead of "dependant"
12. Line 891: I cannot open/access the AlgaeFUN github URL (the SANDAL github URL works).

Reviewer #2 (Comments for the Author):

The paper is focused in the investigation of photoperiod in orchestration of Ostreococcus tauri gene RNA and protein content. In addition, the authors present data on free running conditions, both constant dark and constant light, after entrainment under either short or long photoperiods. The authors also provide data on diel oscillations of start content and cell cycle dynamics. This is a high quality dataset and will be an important tool to study the role of rhythmic expression in the physiology of photosynthetic eukaryotes. The authors highlight expression patterns of genes and proteins in a some key processes (photosynthesis, starch metabolism and the cell cycle). Interestingly they observe robust rhythms under constant dark conditions (DD), although previous studies had suggested there were no transcriptional or translational rhythms in DD in O. tauri (O'Neill et al., 2011). To my understanding free running rhythms of cell division were also only detectable under very low light intensities (Moulager et al., 2007). These differences are probably due to the growth condition differences in these studies and also suggest differences in how free running rhythms of different processes are regulated. These observations highlight the importance of the environment in algal physiology and indicates we need to need to study rhythms in the framework of the biology of these organisms under their natural environment.

The manuscript describes high quality datasets, is well organized and clearly written. However, the result section is tedious to read, in particular, the second half when the authors describe some specific processes in detail. In addition, the authors do not go into detail for the apparent discrepancies in the rhythms between different studies until the discussion.

Major issues:
1. I would strongly suggest the authors to focus on the main general outcomes of this study. It is important for the reader to understand the rationale of why the authors look into particular details of the data but also what the authors conclude from those analyses as they read. I think merging the results and discussion sections and having a shorter conclusion section would help highlight the impact of the discoveries made in this study. These are the key outcomes of this study:
a. Robust rhythmicity in constant conditions although for specific sets of genes is present under some conditions. I think showing the differences between studies in starch content is key here as the authors highlight in the discussion. Small marine plankton are likely to be under extended periods of low light at some point, so they need longer term carbon storage in a way that most land plants and other algae living on the surface don't. I would expect that the dawn level of starch varies in O.t. with light intensity, such that they build a 'reserve' under higher light conditions that is not consumed during the regular light/dark cycle but allows them to survive longer times in darkness/low light. Supplemental Figure 9 (and all experimental results) should be addressed first in the results section and not only in the discussion.
b. There a differences in the offset between transcript and protein content for different proteins and different conditions. The section in which they conduct a general assessment of the differences is useful but the details of the specific processes are harder to follow. These extended descriptions are good and important but only of interest to particular researchers and could be moved to the supplemental text. For example, the authors describe in Line 259 that genes peaking during the night period in SD have stronger delays in the peak of protein than transcripts peaking early in the day. Is that because translation might occur preferentially during the light period as suggested by Seaton et al., 2018? This dataset can be used to actually address some of the hypothesis raised by previous studies. That would provide a simpler context to describe this large dataset. I would also suggest also make Fig. 3D and Fig. 3E using their proteomic data, since they make the point there are strong delays between RNA and proteins. The transcriptomic data phases are quite similar to other previous studies in other photosynthetic organisms so they do not add so much new information. The authors should emphasize their more novel and interesting results in the main figures.

2. As the experimental conditions are so critical the authors should:
a. Add panel in Figure 1 showing the actual light intensity at the different times.
b. Add the light intensity used in the constant conditions to the methods section.
c. Describe whether the ZT0 and ZT8/ZT16 samples were collected in the dark or light.
d. Indicate whether cell density or culture absorbance remained constant as it seems that the used the same median replenishment cycles in both LD and SD. Did culture grow faster in LD than SD?

3. When describing the free running conditions the authors should highlight the rhythms of clock components and light receptors and briefly compare their results with the previous studies.

Minor Issues:
*Page 3, Lines 60-65: "The molecular mechanisms that have evolved to anticipate..." are "circadian clocks" and not "circadian rhythms". The molecular mechanisms give rise to circadian rhythm and is not the rhythm itself.

*Line 97: I would just say Short and Long photoperiods and not summer or winter in this particular sentence in which the authors present their particular dataset, as summer and winter would assume changes in temperature. I think it is valid to use summer and winter in a general sense in the discussion.

*Line 116: In the PCAs it seems that the LD transcriptome (and proteome) changes are much better separated than the ones in SD, do the authors have an idea why? Is it correlated with the robustness of the rhythms?

* Figure 1H: the font is much too small to read. Please show only as much can be actually read in the main figure, the other details can be shown in the supplemental.

*Lines 136-143: I think the numbers are wrong as stated. They don't count the genes that were rhythmic in all regimes. These sentences need to be reworded or numbers adjusted. Currently it reads as only X% remains rhythmic when moved to Y condition from Z condition, whereas the numbers refer (based on Figure 1E) seem to refer to X% remains rhythmic exclusively when transferred to Y condition from Z condition.

*Line 143: should be 'see', not 'See'.

*Page 6, Lines 164-167: There seems to be no directional bias on the phase shift for SD DD conditions in the figure referenced.

* Line 180: I would not refer here to 'activation of biological processes' since as the authors here show only RNA content and as their results demonstrate that does not match necessarily with protein content. In line 359 the authors also use 'activate' when describing steady state protein and RNA levels. I would use 'peak' or 'reach maximum levels', activate suggests an activity which is a bit confusing when describing enzymatic genes as their activity could actually be measured as the authors did for NR and GS. In the case of NR and GS (Line 466) it is important to indicate that the maximal catalytic activity was measured, activity in vivo could be influenced by the level of regulatory metabolites.

*Line 386: The authors provide little context of why the cycling of pigments would be of interest including briefly mention their function for the non-expert reader. They could cycle in order to adapt to the differences in light intensity or to accommodate for plastid growth. For all the physiological processes highlighted it would be useful to indicate their degree of cycling under constant conditions, which would suggest circadian control.

*Line 183: it is unclear what the authors mean with 'differences' in this sentence.

* Line 234: There does not seem to be any subcellular localization data in Supp. Figure 7.

*When describing phase differences between RNA and proteins the authors make the assumption that the peak of protein will always be after the RNA and therefore have phase delays of more 12 h. This does not necessarily be the case, however, I understand that it might be ok to make that assumption but then that assumption should be explicitly stated in the results and methods section.

* Page 17, Lines 495-499: Again, clarity in language is needed to emphasize that the authors are only talking about the genes found rhythmic in all the conditions.

* Page 18, Lines 518-520 CCA1 not defined previously.

* In text references: all of the references have the wrong spacing after the citation, some have a space missing before the citation.

Reviewer #3 (Comments for the Author):

Overall, the manuscript provides a detailed, integrated view of circadian and photoperiod-dependent molecular responses in the picoeukaryote Ostreococcus tauri. Although much is already known about molecular circadian rhythms in Ostreococcus, this article expands our understanding of photoperiod-dependent responses by integrating transcriptomic, proteomic, enzymatic, metabolite, and other physiological analyses. In my opinion, the data is of good quality and thus adds value to the community. Despite the manuscript's descriptive nature, I believe it deserves to be published in The Plant Cell, considering the quality and exhaustiveness of the presented data.

Below are some of my comments:

The manuscript should be presented in a way that contributes to the understanding of chronobiology in chlorophytes, beyond just Ostreococcus. Efforts should be made to explain how this paper adds to the existing knowledge of chlorophytes (plants and green algae). I would also appreciate a paragraph discussing the possible mechanisms regulating photoperiodic responses, although this is not compulsory given the extensive nature of the paper. There is substantial bibliography in land plants (green lineage) about many processes controlled in a photoperiod-dependent manner (flowering time, growth, etc.) and by which means (co-incidence mechanisms between the clock and the environment, such as the EC complex and PhyB or GI/ZTL).

Minor comments:

Title:
Adaptation typically refers to genetic changes. In this case, "acclimation" or "phenotypic plasticity" would better describe photoperiod-related changes in molecular rhythms.
Introduction:
Lines 52-54: Seasons occur only at certain latitudes of the planet.
Lines 60-61: Biological rhythms are not restricted to circadian rhythms. This phrase is somewhat awkward; the molecular mechanisms that have evolved to anticipate circadian cycles are referred to as circadian clocks.
Lines 64-65: Of about 24 hours.
At the end of the introduction, the authors enumerate all the chronobiological studies in Ostreococcus; however, it reads more like a list than an introductory paragraph. The authors should make a better effort to explain the importance of all these studies and how the presented work adds to this data. Information about the composition of the Ostreococcus clock is not introduced at all. It has a reduced plant-like clock (LHY-TOC), which should be explained.

Results:

Lines 126-127: No samples were collected during the first day of constant conditions. This information should be shown in the figures. This means that samples in free-running conditions were collected from CT24 onwards (and not CT0).
Line 205: The phases of approximately two-thirds (63%) of the rhythmic genes were successfully predicted with {plus minus}4h error. The error seems quite large; can the authors comment on this and thus on the strength of the predictive model?
Bimodal oscillations: Do all bimodal oscillations observed in SD show a single peak in LD? and are all the bimodal oscillations explained by the SD to LD model?
Cell Cycle: The authors should specify in the results that different phases of the cell cycle were determined by assessing total DNA content.
Figures 7C/8A/8D: The boxes with the different gene/protein expressions in LD and SD are not very self-explanatory.

Discussion:
Can the authors comment on the "nocturnal nature of the transcriptome"? Could the escape from light theory be advanced here?
Line 515: Could the fact that animal cells maintain stronger oscillations be explained by the fact that these are multicellular organisms and that the assessed cells are likely those of the SCN (which don't sense light changes)?

Reviewing Editor comments:
We would recommend mitigating the environmental implications of your study in the abstract as well as in the main text, and to focus more on photoperiodic regulation than seasonal adaptation. Ostreococcus is indeed a powerful experimental model system for green algae, but unlike other pico-eukaryotes such as Bathycoccus or Micromonas, it is not abundant in marine environments. It has been found in some lakes and in very few coastal regions. We advise the authors to refer to the recent bibliography reporting abundance of different phytoplankton groups at a global scale level and also at already available studies on seasonality of marine picoeukaryotes (e.g., Lambert S, et al., ISME J. 2019, and others).

<u>Response to Editors:</u>

More specifically, the reviewers consider that the paper lacks critical comparisons with similar studies already carried out in other systems. After consultation of the reviewers with the editorial board, we recommend extending the computational analysis of your data to identify conserved or possibly novel regulators involved in photoperiodic regulation in Ostreoccoccus. If possible, on the basis of the genomic information available in Ostreococcus, the authors could also consider analyzing potential cis-regulatory elements in the promoter regions of the rhythmic genes. When compared to results from land plants, this analysis could provide some potential regulatory insight. Any effort at a comparative analysis, bioinformatic and editorial, will help the general audience of the journal grab onto the manuscript and strengthen its impact.

We highly appreciate the editorial work as they have effectively summarized all reviewers' comments and suggested specific new analysis that have definitely broadened and enhanced the study presented in our manuscript. As suggested by the editors, we have made an effort to provide regulatory mechanisms to facilitate comparisons with other photosynthetic organisms. We have performed a Transcription Factor Binding Site enrichment analysis over the promoters of genes peaking at specific time points under LD and SD conditions. A new table, Table 1, has been added with the corresponding results. Notably, the Evening Element (EE) motif, associated to the binding site of CCA1 in Arabidopsis, was found significantly enriched in the gene sets corresponding to ZT12 under LD and ZT0/ZT4 under SD. We have also developed a model based on sPLS (sparse Partial Least Squares) integrating the transcriptomic and physiological data generated in this study. This model identified some TFs belonging to specific families as potential regulators of photoperiodic responses in rhythmic physiology. This model also identified CCA1 as a potential regulator for starch content, cell cycle progression and carotenoid content as a response to photoperiod length. The identification of the EE motif in the promoters of genes encoding key proteins in these processes further supported the key role played by the CCA1 ortholog ostta06g02340. A new figure, Figure 11, has been added representing this model and a final paragraph discussing this model has been added to the Discussions section. We have also included comparisons with similar studies in the Introduction, Results and Discussions.

Also, I noticed that your figure panel labels utilize an oversized serif font (Times, I think) in parentheses, and the journal requirement generally is a sans serif font such as Arial. More specifically, we don't use parenthesis, and the panel marker is just a bold capital letter; we can also fix this later, but it might save some work to do it now. Please check your supplemental figures too.

We have changed all figure panel labels to capital letters in Arial in the current version.

We noticed that a small number of your figures utilize red/green contrasts, so as you prepare the final version of the figures, please check the figures for red/green color use. Magenta is a good substitute for red, and a light blue substitutes for green.

Figure colors has been adjusted as suggested by the editor to magenta and light blue instead of red and green.

We would recommend mitigating the environmental implications of your study in the abstract as well as in the main text, and to focus more on photoperiodic regulation than seasonal adaptation. Ostreococcus is indeed a powerful experimental model system for green algae, but unlike other pico-eukaryotes such as Bathycoccus or Micromonas, it is not abundant in marine environments. It has been found in some lakes and in very few coastal regions. We

advise the authors to refer to the recent bibliography reporting abundance of different phytoplankton groups at a global scale level and also at already available studies on seasonality of marine picoeukaryotes (e.g., Lambert S, et al., ISME J. 2019, and others).

Following the editors' suggestion we have removed "seasonal adaptation" from the title which reads now: "Unveiling Photoperiodic Plasticity in Molecular Rhythms in Marine Phytoplankton through Multiomics Integration".

The sentences regarding the abundance of Ostreococcus in the ocean has been removed from the abstract and introduction as suggested by the editors.

<u>Response to Reviewer #1:</u>

This article describes the generation and analysis of an extensive transcriptomic, proteomic, and physiological dataset profiling diel cycles in the picoeukaryote Ostreococcus tauri. The availability of different omics layers allowing studying specific responses, their (dis)agreements or temporal shifts at the transcript and protein level, and the effect diel cycles have on different gene functions and pathways.
Overall, the manuscript is well written and easy to follow. The authors wrote a good Introduction which does a great job on explaining key concepts (but see comment 1). While the study is overall sound, I am not fully convinced of the ground-braking nature and novelty of this manuscript. While the generated dataset is unique and present a great resource for the research community, the Results section is very descriptive. A missed opportunity is that no/limited new insights are reported about e.g. the mechanisms of the underlying gene regulation (transcriptional and/or at the protein level). Furthermore, as similar datasets exist in related unicellular/green eukaryotes (e.g. Kingdom-wide comparison reveals the evolution of diurnal gene expression in Archaeplastida ; https://www.nature.com/articles/s41467-019-08703-2), an in-depth cross-species analysis has the potential to uncover new findings about e.g. conserved genes or functional features, which could generate new testable hypotheses.

We appreciate the reviewer's recognition of our integrative analysis of diel cycles which reveals photoperiod specific multiomics rhythms underlying key physiological processes. We are pleased s/he valued our study of the global orchestration between transcriptome, proteome, and physiological dynamics with photoperiod specific temporal offsets between the timing of transcripts, proteins, and physiological responses. We appreciate his/her comments and suggestions, all of them has been taken into account, as discussed below, and this has definitely improved the manuscript.

We understand that the reviewer finds the work very descriptive and to provide regulatory mechanisms to facilitate comparisons with other photosynthetic organisms, we have performed a Transcription Factor Binding Site enrichment analysis on the promoters of genes peaking at specific time points under LD and SD conditions. Additionally, we have developed an integrative model based on sPLS (sparsed Partial Least Squares) between our omic and physiological data. Both analysis have revealed specific families of transcription factors and a central role played by the CCA1 ortholog, indicating a high level of conservation at the core of the circadian clocks in Ostreococcus and Arabidopsis.

We have included in the current version of the manuscript a reference to the excellent work in "Kingdom-wide comparison reveals the evolution of diurnal gene expression in Archaeplastida" (https://www.nature.com/articles/s41467-019-08703-2). Although our approach is markedly different, this referenced paper only considers transcriptomics, whereas our analysis shows that the relationship between transcriptomic, proteomic and physiological dynamics is not straightforward. Our analysis suggests strong post-transcriptional regulation resulting in different time offsets between rhythmic transcripts and proteins depending on the photoperiod and the specific biological process, with shorter offsets observed between proteome and physiological dynamics. This highlights the need of multiomics analysis to characterize the photoperiodic plasticity of molecular rhythms in the green lineage. Nonetheless, this comparative analysis exceeds the scope of the study presented here. Nonetheless, this paper could serve as a basis for such studies since the experimental design and analysis methods used here could be extended to other organisms in the green lineage. This is the current line of research in our group, to study the evolutionary conservation or diversification in the multiomic response to photoperiod in the green lineage for which this study is the basis from the methodological and analytical standpoint.

Major

1. Introduction: while the different terms and problem statement is clearly defined, I am missing information on what previous papers have reported on the molecular analysis of diel rhythms (lines 56-57 and 73-75 remain vague). Please add a few sentences explaining the key findings of these papers, which will give more context to the newly presented results.

We agree that a brief description of the previous results was missing at this point. We have added a few sentences explaining them, as suggested by the reviewer.

2. While information on replicates is available for the proteome profiling, information on replicates is missing for the RNA-Seq transcript profiling (unless I missed it).

We apologise for having forgotten to include the number of replicates in the RNA-seq data. Three different replicates were analysed for each data point under LD and SD conditions. This information has now been added to the materials and method section.

3. Lines 205-... The observation of two peaks per day (bimodal rhythmic expression) is interesting and reminds me of a similar paper published in diatoms some years ago (https://pubmed.ncbi.nlm.nih.gov/33901335/). While the authors here briefly cite two papers in plants and animals (line 585), extending this analysis, and/or the discussion, would allow to put these results in a broader context and could confirm/refute hypotheses formulated about the biological significance of bimodal rhythmic expression.

This is an excellent point raised by the reviewer. Indeed, we initially considered that bimodal expression patterns could be circatidal. However, we discarded this possibility because our experimental design did not simulate tidal conditions in any way and because Ostreococcus is described as a planktonic picoalga. More importantly, if bimodal expression patterns were truly circatidal, the 12 h period expression patterns should have been maintained under free-running conditions. Instead, the bimodal expression patterns disappeared under free-running conditions resulting in profiles that exhibited a single peak with a 24 h period.
These comments and the corresponding reference have now been included in the discussions section as we agree this broaden the context of our manuscript.

4. Methods: for both the RAIN and CircaCompare rhythmic analyses, it is mentioned that a "p-value threshold was used". What kind of multiple testing correction was performed for these analyses, to correct for significance testing of a large number of genes, or proteins, or physiological parameters? Please explain the multiple-testing strategy clearly in the Methods.

We agree that these details need to be specified. P-values were corrected in the corresponding R packages using the Benjamini-Hochberg correction for multiple testing. This information has now been added to the materials and methods section.

5. Methods: RAIN was used to determine the significance of rhythmicity, while circaCompare was used to compare rhythmic parameters (phase, amplitude etc). Why not use only circaCompare for everything since it is also able to determine the significance of rhythmicity? Explain the rationale.

RAIN implements a non-parametric method to identify rhythmic that does not assume any specific form in the rhythmic expression profiles. In contrast, CircaCompare consists of a parametric method in which expression profiles are identified as rhythmic if they can be fitted to a cosinusoidal

wave. Therefore, from a theoretical point of view, RAIN would be more appropriate for determining rhythmicity. This is why we first applied RAIN in our analysis. However, we also executed CircaCompare obtaining approximately the same sets of rhythmic genes (see Venn diagram below). This suggests that, for our data, the rhythmic patterns can indeed be modeled as cosinusoidal waves, allowing us to perform the significance analysis of the differences in phases and amplitudes using CircaCompare. This is not always the case, rhythmic data cannot always be approximated by parametric methods necessitating the application of different methods from those implemented in CircaCompare.
We have now included this rationale in the materials and methods explaining that the non-parametric method implemented in RAIN was used first since it searches for any type of rhythmic patterns. The methods in CircaCompare were also employed identifying approximately the same rhythmicity. Once we confirmed that our data could be further analysed using the paremetric methods in CircaCompare, we proceeded to analyse significance of differences in phases and amplitudes using CircaCompare.

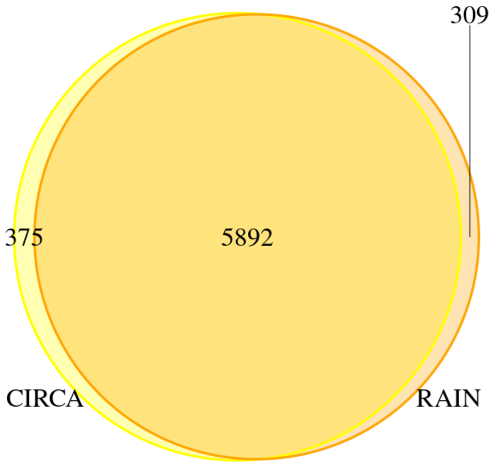

6. Line 159: it is mentioned that a transfer to DD would increase the amplitude, but this is not shown in the figure 2 D (in other panels, a significant change is shown by the p-value). Are you sure it was significant?

We thank the reviewer for raising this point. All the authors have discussed it and although there is a significant increase, it is relatively small and therefore unlikely to have any substantial physiological effect globally. Consequently, we have remove this point stating that no substantial effect was observe in amplitude when cultures were transferred from SD to DD.

7. Line 159: I wonder if only the amplitudes of the significantly rhythmic genes are being considered here? Because the number of rhythmic genes is much lower in DD (post SD) compared to SD, you would expect that the average amplitude should drop considerably. If it is true that you only consider the amplitudes of the significantly rhythmic genes, this should be stated more clear, as it might simply reflect that only the genes with the highest amplitudes are still rhythmic in DD.

We agree with the reviewer that this point needs to clarification. Only the rhythmic genes under the specific photoperiod and free-running conditions were considered. Indeed, we believe that the drastic reduction in amplitude produced by the desynchronization under LL could be responsible for

the significant decrease in the number of rhythmic genes. This aligns with what the reviewer suggests, highly rhythmic genes are expected to have large amplitudes While these amplitudes would be affected by free-running conditions reducing them, they would still be detectable as rhythmic.
This has been modified in the current version of the manuscript.

8. Figure 5, panel C: this is not the best visualization as the GO identifiers are very small/hard to read and nobody knowns these identifiers by hard. Therefore, add GO description next to all or a selection of relevant term and consider removing some terms (e.g. those that are redundant with selected terms or those that are not discussed in the text).

As suggested by the reviewer GO descriptions of the most relevant terms has been added to improve readability in Figure 5 C.

Minor
9. Line 164: Please explain what "advanced phases" are

In chronobiology, "advanced phases" refer to shifts in the timing of a biological rhythm so that an event or activity occurs earlier in the cycle compared to a reference point. We have clarified this in the text as suggested by the reviewer.

10. Line 266: Revise the title of this section: Combining proteome and ... to

We thank the reviewer this suggestion regarding the section title. We have carefully considered the proposed change. However, we believe that the original title, 'Proteome and transcriptome rhythmicity combine to orchestrate physiological rhythms,' better reflects the active role of both proteome and transcriptome rhythmicity in regulating physiological rhythms, as discussed in the section. It also captures the interplay and coordination highlighted in the content. Therefore, we have decided to keep the current title unless otherwise stated by the reviewer being open to further discussion if needed.

11. Line 578: "dependent" instead of "dependant"

We thank this reviewer for spotting this typo. This has now been corrected.

12. Line 891: I cannot open/access the AlgaeFUN github URL (the SANDAL github URL works).

We thank this reviewer for his throughout check. There was a typo in the AlgaeFUN github URL. This has now been corrected.

Response to Reviewer #2:

The paper is focused in the investigation of photoperiod in orchestration of Ostreococcus tauri gene RNA and protein content. In addition, the authors present data on free running conditions, both constant dark and constant light, after entrainment under either short or long photoperiods. The authors also provide data on diel oscillations of start content and cell cycle dynamics. This is a high quality dataset and will be an important tool to study the role of rhythmic expression in the physiology of photosynthetic eukaryotes. The authors highlight expression patterns of genes and proteins in a some key processes (photosynthesis, starch metabolism and the cell cycle). Interestingly they observe robust rhythms under constant dark conditions (DD), although previous studies had suggested there were no transcriptional or translational rhythms in DD in O. tauri (O'Neill et al., 2011). To my understanding free running rhythms of cell division were also only detectable under very low light intensities (Moulager et al., 2007). These differences are probably due to the growth condition differences in these studies and also suggest differences in how free running rhythms of different processes are regulated. These observations highlight the importance of the environment in algal physiology and indicates we need to need to study rhythms in the framework of the biology of these organisms under their natural environment.

The manuscript describes high quality datasets, is well organized and clearly written. However, the result section is tedious to read, in particular, the second half when the authors describe some specific processes in detail. In addition, the authors do not go into detail for the apparent discrepancies in the rhythms between different studies until the discussion.

We would like to thank this reviewer for his/her thorough review and insightful comments on our manuscript. We appreciate his/her recognition of the quality and relevance of our dataset, as well as his/her constructive feedback regarding the presentation of our results. We understand that certain sections, particularly the detailed presentation of specific processes, may come across as tedious and descriptive. In the new version of the manuscript we have made an effort to provide regulatory mechanisms to facilitate comparisons with other photosynthetic organisms. We have performed a Transcription Factor Binding Site enrichment analysis on the promoters of genes peaking at specific time points under LD and SD conditions. Additionally, we have developed an integrative model based on sPLS (sparsed Partial Least Squares) between our omic and physiological data. Both analysis have revealed specific families of transcription factors and a central role played by the CCA1 ortholog, indicating a high level of conservation at the core of the circadian clocks in Ostreococcus and Arabidopsis. We also acknowledge the importance of comparing our findings and previous studies, and we have expanded on these points in several sections of the paper. Next, we have responded to each of this reviewer’s specific concerns.

Major issues:

1. I would strongly suggest the authors to focus on the main general outcomes of this study. It is important for the reader to understand the rationale of why the authors look into particular details of the data but also what the authors conclude from those analyses as they read. I think merging the results and discussion sections and having a shorter conclusion section would help highlight the impact of the discoveries made in this study.

We fully understand these comments. However, *The Plant Cell* journal does not allow this organization in research papers requiring separate sections for results and discussion. Therefore, we have followed the author guidelines and have structured our paper accordingly.

These are the key outcomes of this study:
a. Robust rhythmicity in constant conditions although for specific sets of genes is present under some conditions. I think showing the differences between studies in starch content is key here as the authors highlight in the discussion. Small marine plankton are likely to be under extended periods of low light at some point, so they need longer term carbon storage in a way that most land plants and other algae living on the surface don't. I would expect that the dawn level of starch varies in O.t. with light intensity, such that they build a 'reserve' under higher light conditions that is not consumed during the regular light/dark cycle but allows them to survive longer times in darkness/low light. Supplemental Figure 9 (and all experimental results) should be addressed first in the results section and not only in the discussion.

We fully agree with this suggestion and have moved the study about starch content to its corresponding section in the results.

b. There a differences in the offset between transcript and protein content for different proteins and different conditions. The section in which they conduct a general assessment of the differences is useful but the details of the specific processes are harder to follow. These extended descriptions are good and important but only of interest to particular researchers and could be moved to the supplemental text. For example, the authors describe in Line 259 that genes peaking during the night period in SD have stronger delays in the peak of protein than transcripts peaking early in the day. Is that because translation might occur preferentially during the light period as suggested by Seaton et al., 2018? This dataset can be used to actually address some of the hypothesis raised by previous studies. That would provide a simpler context to describe this large dataset. I would also suggest also make Fig. 3D and Fig. 3E using their proteomic data, since they make the point there are strong delays between RNA and proteins. The transcriptomic data phases are quite similar to other previous studies in other photosynthetic organisms so they do not add so much new information. The authors should emphasize their more novel and interesting results in the main figures.

We agree that our data also suggests that translation occur preferentially during the light period, contrary to transcription that takes place mostly during the dark period. A sentence commenting on this has been now included in the current version of the paper. We have also followed the suggestion by this reviewer and have included a new panel in Figure 4 representing diel timing of processes based on the time point when the protein abundances reach their maximum.

2. As the experimental conditions are so critical the authors should:
a. Add panel in Figure 1 showing the actual light intensity at the different times.

We agree with this reviewer that the experimental design in our study is crucial. Accordingly, the experimental design, including the actual light intensities over complete photoperiods, is presented in Supplemental Figure 1. Incorporating all this information as a new panel into the current Figure 1, which already has nine panels, would create a figure too complex and possibly exceeding the journal limits. Unless this reviewer argues otherwise and the editors allow it, we prefer to keep the experimental design in Supplemental Figure 1.

b. Add the light intensity used in the constant conditions to the methods section.

This information has now been included in the materials and methods section.

c. Describe whether the ZT0 and ZT8/ZT16 samples were collected in the dark or light.

ZT0 was collected immediately after lights were on; and ZT8 (under SD conditions) and ZT16 (under LD conditions) were collected immediately after light were off. This information has now been added to the materials and methods section.

d. Indicate whether cell density or culture absorbance remained constant as it seems that the used the same median replenishment cycles in both LD and SD. Did culture grow faster in LD than SD?

Ostreococcus cultures grow faster under LD than SD conditions as mentioned in the cell cycle analysis section. Nevertheless, in our experimental design, cell density was similar for both conditions due to different dilution rates. Fresh medium was continuously added during the light period in both conditions at a flow rate of 45 mL h-1. Since the photoperiod under LD is twice as long as under SD, the dilution rates were different, being 0.4 d-1 under LD and 0.2 d-1 under SD. These differences ensured similar cell densities in both conditions. Our cultures were operated under continuous mode which maintained culture absorbance stable during the experiment. This has now been highlighted in the materials and methods sections.

3. When describing the free running conditions the authors should highlight the rhythms of clock components and light receptors and briefly compare their results with the previous studies.

We understand the point raised here by this reviewer. Accordingly, the gene expression patterns of CCA1 under free-running conditions has been presented in Supplemental Figure 9 and they have been validated using RT-qPCR to show that the clock is still functioning under constant dark. A new supplementary figure has also been included with the expression patterns of the clock central components and light receptors indicating that they oscillate even under constant dark in contrast to previous reports generated under different growth conditions. Nevertheless, extending this analysis would imply to include a new section which would increase substantially the length of the paper possibly beyond the limits imposed by the journal. As indicated by this reviewer, our multiomics data could be the base for complex analysis regarding different molecular systems in Ostreococcus. In order to facilitate the exploration of our data by independent researchers with specific interest different from the ones presented in this paper, we have developed the online app MINOTAUR (https://greennetwork.us.es/MINOTAUR/). Additionally, we have provided in the GitHub repository SANDAL (https://github.com/fran-romero-campero/SANDAL) the scripts used in the analysis presented in this paper so it can be modified and extended to perform other analysis. We have explicitly included these links in the “Data and code availability” section.

Minor Issues:

***Page 3, Lines 60-65: "The molecular mechanisms that have evolved to anticipate..." are "circadian clocks" and not "circadian rhythms". The molecular mechanisms give rise to circadian rhythm and is not the rhythm itself.**

We thank this reviewer for spotting this mistake that has been corrected in the current version of the manuscript.

***Line 97: I would just say Short and Long photoperiods and not summer or winter in this particular sentence in which the authors present their particular dataset, as summer and**

winter would assume changes in temperature. I think it is valid to use summer and winter in a general sense in the discussion.

We agree with this reviewer that in the introduction it is more appropriate to refer to long/short photoperiod to avoid misleading readers into thinking that different temperatures are also considered. We have edited the text accordingly.

***Line 116: In the PCAs it seems that the LD transcriptome (and proteome) changes are much better separated than the ones in SD, do the authors have an idea why? Is it correlated with the robustness of the rhythms?**

The rhythmicity under SD is more complex than under LD. While almost no genes exhibit bimodal rhythmicity under LD, nearly almost 2000 genes show bimodal rhythmicity under SD conditions. This pattern is also reflected in the proteome. We believe this contributes to the the circular organization observed in the PCA for LD whereas for SD samples arrange more into an ellipse. Additionally, the amplitudes of rhythmic genes and proteins are greater under LD compared to SD possibly as a result of a reduced synchrony in SD cultures compared to LD cultures. However, discussing all this when presenting the PCA results, and/or referring back to it later in the paper, may make the study difficult to follow. Therefore, unless the reviewer insists otherwise, we have decided not to modify this part of the manuscript to include these discussions.

*** Figure 1H: the font is much too small to read. Please show only as much can be actually read in the main figure, the other details can be shown in the supplemental.**

We agree with this reviewer on this comment and the corresponding figure has been adjusted accordingly.

***Lines 136-143: I think the numbers are wrong as stated. They don't count the genes that were rhythmic in all regimes. These sentences need to be reworded or numbers adjusted. Currently it reads as only X% remains rhythmic when moved to Y condition from Z condition, whereas the numbers refer (based on Figure 1E) seem to refer to X% remains rhythmic exclusively when transferred to Y condition from Z condition.**

This reviewer is right in this point and we have reworded the sentence to indicate that we are talking about the transcriptome proportion that maintained oscillations exclusively under LL or DD and not under both.

***Line 143: should be 'see', not 'See'.**

This has been changed as indicated by this reviewer.

***Page 6, Lines 164-167: There seems to be no directional bias on the phase shift for SD DD conditions in the figure referenced.**

We agree with the reviewer that indeed the phase shift for LD/DD is much more pronounced than the phase shift for SD/DD as shown in the histograms Figure 2 B (right) and Figure 2 E (right). Nevertheless, a negative bias can still be observed in the histrogram corresponding to SD/DD. As suggested by the reviewer, this part of the manuscript has been edited to include this.

*** Line 180: I would not refer here to 'activation of biological processes' since as the authors here show only RNA content and as their results demonstrate that does not match necessarily with protein content. In line 359 the authors also use 'activate' when describing steady state**

protein and RNA levels. I would use 'peak' or 'reach maximum levels', activate suggests an activity which is a bit confusing when describing enzymatic genes as their activity could actually be measured as the authors did for NR and GS. In the case of NR and GS (Line 466) it is important to indicate that the maximal catalytic activity was measured, activity in vivo could be influenced by the level of regulatory metabolites.

We agree with this reviewer in these points and the text has been edited following his/her suggestions.

***Line 386: The authors provide little context of why the cycling of pigments would be of interest including briefly mention their function for the non-expert reader. They could cycle in order to adapt to the differences in light intensity or to accommodate for plastid growth. For all the physiological processes highlighted it would be useful to indicate their degree of cycling under constant conditions, which would suggest circadian control.**

We have explicitly mentioned the interest of studying carotenoids in the context of diel cycles to understand their plasticity in adjusting their rhythms to photoperiodic variations in diel cycles and their implications in optimizing light energy capture and photoprotection.

We appreciate the reviewer's suggestion regarding the analysis of the degree of cycling in physiological processes under constant conditions, as it could indeed provide valuable insights into circadian physiology. However, our current study focused specifically on circadian gene expression and free-running conditions were only analysed for transcriptomic data. The assessment of physiological rhythms under constant conditions was not part of our experimental design. Additionally, including this type analysis would extend the scope and complexity of the paper which is beyond what we can accommodate at this stage. We consider this an interesting line for future research.

***Line 183: it is unclear what the authors mean with 'differences' in this sentence.**

We were referring to differences in diel timings. This has now been explicitly mentioned in the current version of the manuscript.

*** Line 234: There does not seem to be any subcellular localization data in Supp. Figure 7.**

The subcellular localization information is shown in panel B. The reference to Supplemental Figure 7B has been updated in the current version of the manuscript to clearly indicate this.

***When describing phase differences between RNA and proteins the authors make the assumption that the peak of protein will always be after the RNA and therefore have phase delays of more 12 h. This does not necessarily be the case, however, I understand that it might be ok to make that assumption but then that assumption should be explicitly stated in the results and methods section.**

This has been explicitly mentioned in the manuscript in the results section and in material and methods.

*** Page 17, Lines 495-499: Again, clarity in language is needed to emphasize that the authors are only talking about the genes found rhythmic in all the conditions.**

This sentence has been reworded following this reviewer’s suggestions.

*** Page 18, Lines 518-520 CCA1 not defined previously.**

In the current version of the paper CCA1 is defined in the introduction.

*** In text references: all of the references have the wrong spacing after the citation, some have a space missing before the citation.**

We have checked this through the manuscript and have corrected the wrong spacing when found.

Response to Reviewer #3:

Overall, the manuscript provides a detailed, integrated view of circadian and photoperiod-dependent molecular responses in the picoeukaryote Ostreococcus tauri. Although much is already known about molecular circadian rhythms in Ostreococcus, this article expands our understanding of photoperiod-dependent responses by integrating transcriptomic, proteomic, enzymatic, metabolite, and other physiological analyses. In my opinion, the data is of good quality and thus adds value to the community. Despite the manuscript's descriptive nature, I believe it deserves to be published in The Plant Cell, considering the quality and exhaustiveness of the presented data.

We sincerely appreciate the reviewer's positive assessment of our work. We are pleased that s/he recognized our detailed, integrated analysis of circadian and photoperiod-dependent molecular responses in Ostreococcus tauri aiming to expand the understanding of these rhythms by multiomics integration. We are glad that the quality and exhaustiveness of our data and analysis were recognized. The reviewer's constructive comments and suggestions, all of which we have carefully addressed, have definitely further enhanced our manuscript.

Below are some of my comments:

The manuscript should be presented in a way that contributes to the understanding of chronobiology in chlorophytes, beyond just Ostreococcus. Efforts should be made to explainhttps://www.juntadeandalucia.es/economiaconocimientoempresasyuniversidad/sguit/GestionMasteres/public/vermaster how this paper adds to the existing knowledge of chlorophytes (plants and green algae). I would also appreciate a paragraph discussing the possible mechanisms regulating photoperiodic responses, although this is not compulsory given the extensive nature of the paper. There is substantial bibliography in land plants (green lineage) about many processes controlled in a photoperiod-dependent manner (flowering time, growth, etc.) and by which means (co-incidence mechanisms between the clock and the environment, such as the EC complex and PhyB or GI/ZTL).

We agree with the point raised by this reviewer, regarding the need to include some mechanistic information and its possible conservation in the green lineage. In this respect, we have performed a Transcription Factor Binding Site enrichment analysis over the promoters of genes peaking at specific time points under LD and SD conditions. A new table, Table 1, has been added with the corresponding results. Notably, the Evening Element (EE) motif, associated to the binding site of CCA1 in Arabidopsis, was found significantly enriched in the gene sets corresponding to ZT12 under LD and ZT0/ZT4 under SD.
We have also developed a model based on sPLS (Sparse Partial Least Squares) integrating the transcriptomic and physiological data generated in this study. This model identified some TFs belonging to specific families as potential regulators of photoperiodic responses in rhythmic physiology. This model also identified CCA1 as a potential regulator for starch content, cell cycle progression and carotenoid content as a response to photoperiod length. The identification of the EE motif in the promoters of genes encoding key proteins in these processes further supported the key role played by the CCA1 ortholog ostta06g02340. A new figure, Figure 11, has been added representing this model and a final paragraph discussing this model has been added to the Discussions section.

Minor comments:

Title:

Adaptation typically refers to genetic changes. In this case, "acclimation" or "phenotypic plasticity" would better describe photoperiod-related changes in molecular rhythms.

We thank this reviewer for pointing this out. All authors have discussed this point and agreed with him/her that acclimation is more accurate to describe the analysis performed in this study. The title has been changed accordingly in the current version.

Introduction:

Lines 52-54: Seasons occur only at certain latitudes of the planet.

We agree on this with this reviewer and have added that seasons only occur in the regions between the tropics and the polar circles (temperate regions).

Lines 60-61: Biological rhythms are not restricted to circadian rhythms. This phrase is somewhat awkward; the molecular mechanisms that have evolved to anticipate circadian cycles are referred to as circadian clocks.

We agree with this reviewer and the text has been modified following his/her suggestion.

Lines 64-65: Of about 24 hours.

We agree with the reviewer that this needs to be included in the introduction and has edited the manuscript accordingly.

At the end of the introduction, the authors enumerate all the chronobiological studies in Ostreococcus; however, it reads more like a list than an introductory paragraph. The authors should make a better effort to explain the importance of all these studies and how the presented work adds to this data. Information about the composition of the Ostreococcus clock is not introduced at all. It has a reduced plant-like clock (LHY-TOC), which should be explained.

Following the suggestion by this reviewer, the last part of the introduction has been rewritten to better introduce previous work in Ostreococcus, explain how our study contributes to expand the current knowledge in this picoalga and compare the structure of the Ostreococcus and Arabidopsis circadian clocks.

Results:

Lines 126-127: No samples were collected during the first day of constant conditions. This information should be shown in the figures. This means that samples in free-running conditions were collected from CT24 onwards (and not CT0).

We thank this reviewer for his/her comment regarding the representation of sampling time in free-running conditions. According to the most common circadian biology conventions, Circadian Time (CT) values range from 0 to 24 to represent a complete 24-hour cycle, with CT0 marking the beginning of the subjective day. Therefore, to remain consistent with these most widely used conventions, we would prefer to keep the CT values within this range. To address this reviewer’s fair concern, we have added an explanatory phrase in the figure legends, indicating that the samples collected during free-running conditions started on the second cycle, starting from CT0 on the second day of free-running conditions. Additionally, a discontinuity in the time axes of the corresponding figures has been added to represent this.

Line 205: The phases of approximately two-thirds (63%) of the rhythmic genes were successfully predicted with {plus minus}4h error. The error seems quite large; can the authors comment on this and thus on the strength of the predictive model?

Our RNA-seq data used to generate our model and the 12:12 data used for validation, were generated using a 4h time increment, ZT0, ZT4, …, and ZT20. Therefore, if the difference between our prediction and the actual phase was less than 4 hours, it was still considered acceptable. In any case, we have interpreted that the limited predictive power of our model suggests that while two thirds of the rhythmic genes respond to changes in photoperiod by gradually adjusting their phases, the remaining one third exhibit a more complex response to photoperiod changes. This has now been included in the results and discussion sections.

Bimodal oscillations: Do all bimodal oscillations observed in SD show a single peak in LD? and are all the bimodal oscillations explained by the SD to LD model?

This reviewer raises an interesting point regarding the source of bimodal oscillations in our data. Indeed, 89.4% of the bimodal genes under SD present a single peak under LD, suggesting that acclimation to SD could be the responsible for generating two peaks per day. The fact that for these genes only one of the peak is maintain under LL and the other one under DD indicates that this bimodal rhythmicity could be the combination of two distinct rhythmic profiles, one dependent on light and the other on dark. Nevertheless, as suggested by another reviewer, initially we considered that bimodal expression patterns could be circatidal with a period of about 12 h. However, we discarded this possibility because our experimental design did not simulate tidal conditions in any way and because Ostreococcus is described as a planktonic picoalga, with no reports on it being benthinc and subjected to tides. More importantly, if bimodal expression patterns were truly circatidal, the 12 h period expression patterns should have been maintained under free-running conditions. Instead, the bimodal expression patterns disappeared under free-running conditions resulting in profiles that exhibited a single peak with a 24 h period.
These comments have now been included in the discussions section.

Cell Cycle: The authors should specify in the results that different phases of the cell cycle were determined by assessing total DNA content.

We agree with the reviewer that this point should be included in the results and the text has been modified accordingly.

Figures 7C/8A/8D: The boxes with the different gene/protein expressions in LD and SD are not very self-explanatory.

We have make an effort to clarify their meaning in the legend of the corresponding figures. If it is still not clear we would very much appreciate any suggestion on how to improve it.

Discussion:
Can the authors comment on the "nocturnal nature of the transcriptome"? Could the escape from light theory be advanced here?

We thank this reviewer for this suggestion, all authors have agreed that it is relevant to discuss it. Indeed, the nocturnal character of the Ostreococcus transcriptome is consistent with the "Escape from light" theory. During transcription, DNA is unwound to allow RNA polymerases to access the template strand, leaving it in a more exposed state, making it more susceptible to light-induced damage. To mitigate this risk, Ostreococcus might concentrate transcription predominantly during the night to avoid the harmful effects of daylight.

These comments have been added to the updated version of the manuscript.

Line 515: Could the fact that animal cells maintain stronger oscillations be explained by the fact that these are multicellular organisms and that the assessed cells are likely those of the SCN (which don't sense light changes)?

We agree with this reviewer that the liver and neuronal cells analyzed in these studies are under constant darkness in these multicellular organisms and do not sense light directly. Hence one would expect that DD free-running conditions would not affect them significantly. Nevertheless, light sensing in animals, plants and algae is markedly different. The authors of this paper are not experts on animal light sensing through for example hormonal signaling or other pathways. Discussing these points would be very speculative for us and we would prefer to leave this discussion out of the paper unless the reviewer thinks otherwise.

2nd Revision - Editorial Decision 15-Nov-2024

Dear Dr. Francisco Romero-Campero:

We have received reviews of your manuscript entitled "Unveiling Photoperiodic Plasticity in Molecular Rhythms in Marine Phytoplankton through Multiomics Integration." On the basis of the advice received, the board of reviewing editors would like to accept your manuscript for publication in The Plant Cell. This acceptance is contingent on revision based on the comments of our reviewers and editors. In particular, please consider the following:

The reviewers and the editorial board agree that this work is interesting and that the authors have addressed most of the previous criticisms during the revision process. However, there is a consensus that the manuscript would benefit from further revisions to improve its readability. This could likely be achieved by reducing repetition in both the main text, legends and figures, improving the logical flow between results. Moreover, a significant portion of the discussion restates the results, which detracts from its value. We believe that a more in-depth discussion of the implications of the results, as well as a clearer focus on the main contributions and discoveries, would make the article more attractive and accessible to a wider audience.

In addition to the comments of the three reviewers, the authors may wish to consider these additional aspects:

- In this work, interesting and important new data derive from the identification of clock-regulated genes in Ostreococcus from the analyses performed in LL and DD (Fig. 1). The authors highlighted differences in the amount of genes regulated by the circadian clock linked to the different LD and SD entrainment. However, these different genes have not really been discussed, also considering the different light irradiation conditions experiences in different photoperiods or in free running.
Moreover, the authors identify a subset of common clock-regulated genes related to photosynthesis or plastid activity. The authors could try to link this information to that in Figures 7, 8 and 9, where they highlight different patterns of photophysiological responses and rhythmic RNA and protein levels. It would be interesting to know whether the 'clock-regulated subset of genes' described in Figure 1 behave differently in SD and LD than those that probably respond only to direct light or metabolic inputs. In other words, would it be possible to identify a 'contribution of the clock' to the photoperiodic responses described in figures 7, 8 or 9? The authors should consider that the circadian clock and photoreceptors, some described in the supplemental Figure 4, are at the heart of photoperiod regulation in plants.

- Intro, line 86, it would be useful to specify the evolutionary position of Ostreococcus in the green lineage. In the way is presented, the current sentence is not informative.

- Line 128, even if the information is reported in the methods, at the beginning of the result session, it will be important to also indicate the light/irradiation intensities and conditions used in LD and SD, and not just the information on the duration of the light/dark cycles, as the different amounts of light received by the cells will certainly play a role in the different rhythmic responses described.

- To make reading more fluid, the authors should avoid describing the results in the legends.

- To facilitate reading, authors could consider to indicate the name of the genes also in the Supp. Figures, close to the IDs.

- In the legend of Fig. 4 ,line 1543, at the end of the text, the description of panel G is missing.

- The authors may consider moving some data into the supplementary. Maybe Fig. 5A is not essential. However, if the authors consider useful to keep the information in the main text, they should make the implication of the analysis and results clearer.

- In Fig.6, Fig6A and C seem to provide very similar information. Are they all necessary?

- Fig. 6B, authors need to make clearer if gene expression profiles refer only to those of Supplementaty Table 6.

- In Figure 7, some of the information in panels C and D, E, F and G appear redundant. The authors may consider retaining only the scheme in panel C and the genes/proteins as in the panels D-G .

- The same in Fig. 8
- Supplementary Tables indicating data on carotenoid and enzymatic activities of Nitrate Reductase (NR) and Glutamine Synthetase miss statistical support

- Table 1 mentioned in line 235 is missing

Please highlight all changes and include a detailed annotation of changes of the text, with line numbers, and noting your responses to the comments.

To submit your revised manuscript, click:

Link Not Available

If you have any questions about the revision submission procedures, please contact the Editorial Office Staff (tpc-submissions@aspb.org). If you cannot return the revised manuscript within 30 days, please let us know. Otherwise, we will assume that you have elected not to revise the manuscript and withdraw it.

Thank you very much for the privilege of reviewing this work. I look forward to receiving the next version.

On behalf of the editorial board,

Reviewing Editor, Board of Reviewing Editors
Daniel Kliebenstein, Senior Editor
Blake Meyers, Editor-in-Chief

The Plant Cell

Reviewer #4 (Comments for the Author):

Comments:

1) The authors have addressed most of my questions and comments. However, I would like to highlight what I think is an important detail. In the paragraph starting in line 458 the authors suggest that low starch accumulation in batch cultured Ostreococcus is mainly caused by the constant supplementation of nitrate and CO2. Algal cells are known for accumulating starch under low nitrogen conditions and therefore it is unlikely that low N is the main limitation causing the differences. If the authors grew the cells as described by O'Neill et al., 2011 the key environmental difference is light intensity, which was 11.5 uE in O'Neill (and includes the use of a blue light filter), which is much lower than the up to 1500 uE used in this study. Light limitation is probably the main reason why the batch cultured cells have low starch. It might be important in this context to mention that Moulager et al., 2007 (PMC1914124) indicated that at higher light intensities (35 uE) Ostreoccocus looses rhythms of cell division. This was probably the reason why Ostreococcus circadian studies have used lower light intensities. The authors' results highlight that rhythmicity varies across physiological responses depending on the environmental conditions.

2) I could not find Table 1.

3) Minor comments:
a. Text spacing is still off for many in text citations
b. Line 253: a reference is needed on transcriptional regulation by CCA1
c. Line 255-257: the authors seem to suggest that the binding of OtCCA1 to potential target genes changes 8-12 h between LD and SD. Is that supported by the transcriptome and/or proteomic data?

Reviewer #5 (Comments for the Author):

The manuscript has certainly improved. Although it remains dense and challenging to read, there have been clear efforts to integrate the datasets (TFBS and sPLS analysis) and to improve the text.

I believe the manuscript deserves publication in The Plant Cell due to the quality and thoroughness of its experiments and data analysis. The authors have addressed my comments satisfactorily (and seemly those of the other reviewers).

Below, I have included a few minor comments.

- Line 96: approaches
- Lines 138/205: Seasonal Variations - The authors should avoid using the term "seasonal variation" throughout the experiments. Please replace it with "photoperiodic variations."
- Line 662: Circatidal rhythms oscillate with a 12.4-hour periodicity. If these bimodal oscillations were circatidal, one would expect a daily phase shift of approximately one hour (which does not seem to be observed here).
- Figure 11: The figure lacks clarity. The legends (e.g., S, Viola, Prasi) do not sufficiently explain the diagram, limiting the reader's understanding of its meaning.

Reviewer #6 (Comments for the Author):

I was asked to replace reviewer 1, who apparently was unavailable to re-review the revised manuscript. I read the manuscript, as well as the reviewers' comments, especially those of reviewer 1.
Below I briefly summarize my own impressions of the revised manuscript and also how this addresses the comments of reviewer 1.

Romero-Losada et al. provide a large combined transcriptomic and proteomic study of the marine algae Ostreococcus tauri, a model system that may not be highly abundant in marine environments, but due to its accessibility has become an important reference system for marine chronobiology. The authors study how their -omics results differ between different photoperiods and constant light conditions (both LL and DD).
Technically, the work is well performed, as already remarked by the previous reviewers.

Major points:

1.)
Conceptually, I cite the initial summary of reviewer 1: "While the generated dataset is unique and present a great resource for the research community, the Results section is very descriptive. A missed opportunity is that no/limited new insights are reported about e.g. the mechanisms of the underlying gene regulation (transcriptional and/or at the protein level)."
This has not changed in my opinion. In fact, also the discussion section is basically a repetition of the results. If the manuscript is meant to go beyond a mere resource, the authors should use the space to derive new insights from their data. Several reviewers, including reviewer 1, suggested to put the data into the context of work from other species (quotation reviewer 1: "an in-depth cross-species analysis has the potential to uncover new findings about e.g. conserved genes or functional features, which could generate new testable hypotheses."). The request for an "in-depth analysis" is not sufficiently addressed by simply citing the paper mentioned by reviewer1.

2.)
Related to the comment above- the main findings of the work are that
a) phases and amplitudes can change between SD, LD, LL and DD.
But what is specifically novel about these findings? Phases changes between SD and LD have long been described for other species.
For LL and DD, the endogenous oscillator is in free-run, meaning it should run by its endogenous period, which is not exactly 24hrs. This automatically implies that there should be phase changes between the entrained conditions and the free-running conditions. Also, it is well established that light intensity under constant conditions has an impact on the circadian clock period length and synchrony (Aschoff, J. Cold Spring Harb Symp Quant Biol. 1960; 25:11-28)- thus, differences between the phases are expected under these free-running conditions. What are the relevant specific novelties and new testable hypothesis derived from this?
b) Transcriptomic and proteomic peaks don't necessarily correlate.
Again, this by itself is a well-established (and published) fact (e.g. https://doi.org/10.1093/bioinformatics/btt537; https://doi.org/10.1186/s12864-019-5749-3). What are the relevant specific novelties and new testable hypothesis derived from this?
c) The proteome cycles less than the transcriptome.
This could be meaningful. However, in the absence of another hypothesis, this could be relatively easily explained by the fact that proteins are often more stable than transcripts. Total protein abundance had been measured (in my understanding), hence if the overall protein turnover is low, this would mask cycling, although the actual synthesis (and degradation) of a given protein could be rhythmic. What would be a more mechanistically interesting hypothesis?
d) The cycling of core circadian clock components, such as cca1 and toc1, under DD, which represents a discrepancy to the work of O'Neill et al, 2011. The authors aim to explain this difference by the different starch content, but it leaves me puzzled as to how low starch content could explain a stop of the core circadian clock or a total desynchronization of the cellular population? If the authors elaborated further on this and integrated known work, this could lead to relevant and testable new hypotheses.

3.) Plotting of individual transcripts- it is unclear if the plot represents a single BR, the mean of all BRs, the median of all BRs? Please indicate what is plotted and also include the range of biological variability. This is particularly relevant when phases are compared, like in Fig.2C,F.

The other major and minor comments of reviewer 1 were all sufficiently addressed in my opinion.

Please also note the following:

-The Plant Cell now requires authors to complete and submit an author revisions checklist upon submission of a revised manuscript. The aim of the checklist is to aid authors in preparing a high-quality manuscript, facilitate the review and assessment of revised manuscripts, and help to ensure that journal standards are maintained across the board. If your manuscript is accepted, the completed checklist will be published as supplemental material attached to the article online. Please download a copy of the checklist (pdf fillable form) at this link, for submission with your revised manuscript: https://tpc.msubmit.net/html/Author_Revisions_Checklist.pdf.

-Supplemental materials should be restricted to large datasets and tables, presentation of replicates, and validation of reagents, methods, or genotypes. Any data that are used to support the major claims must be in the main manuscript. Supplemental figure legends must indicate what figure in the main manuscript is supported by the supplemental data presented. Please justify how each of the supplemental figures meet the criteria.

-Sampling methods and the nature of "biological replicates" should be described precisely (i.e. different plants, parts of plants, pooled tissue, independent pools of tissue, sampled at different times, etc.), along with a clear description of and rationale for any statistical analyses conducted. The reader should know exactly what was sampled; what forms the basis of the calculation of any means and statistical parameters reported. This is also necessary to ensure that proper statistical analysis was conducted.

The reviewers and the editorial board agree that this work is interesting and that the authors have addressed most of the previous criticisms during the revision process. However, there is a consensus that the manuscript would benefit from further revisions to improve its readability. This could likely be achieved by reducing repetition in both the main text, legends and figures, improving the logical flow between results. Moreover, a significant portion of the discussion restates the results, which detracts from its value. We believe that a more in-depth discussion of the implications of the results, as well as a clearer focus on the main contributions and discoveries, would make the article more attractive and accessible to a wider audience.

As indicated by the editors and reviewers we have removed repetitions by eliminating results from figure legends and simplifying figures by moving to supplementary figures parts that could be redundant with other panels. Finally, results have been removed from the discussion section and we have focused on discussing the implications of our results relating them to other studies. This has led to reduce the length of the discussion section to almost half of its original size. We hope this would make the article more accessible and attractive to a wider audience.

In addition to the comments of the three reviewers, the authors may wish to consider these additional aspects:

- In this work, interesting and important new data derive from the identification of clock-regulated genes in Ostreococcus from the analyses performed in LL and DD (Fig. 1). The authors highlighted differences in the amount of genes regulated by the circadian clock linked to the different LD and SD entrainment. However, these different genes have not really been discussed, also considering the different light irradiation conditions experiences in different photoperiods or in free running.

The editors raise an interesting point here that has been addressed in the current version of the manuscript. The circadian genes after LD and SD entrainment have been found to be associated with ribosome biogenesis and cell cycle respectively, indicating a strong influence of the clock over the regulation of these processes. However, circadian genes after LD entrainment lose rhythmicity under LL after SD entrainment, and the reverse is also true, the specific circadian genes after SD entrainment lose rhythmicity under DD after LD entrainment. This suggests a strong detrimental impact on rhythmicity of constant light after SD entrainment with a short photoperiod, as well as constant darkness after LD entrainment with a short dark period.

Moreover, the authors identify a subset of common clock-regulated genes related to photosynthesis or plastid activity. The authors could try to link this information to that in Figures 7, 8 and 9, where they highlight different patterns of photophysiological responses and rhythmic RNA and protein levels. It would be interesting to know whether the 'clock-regulated subset of genes' described in Figure 1 behave differently in SD and LD than those that probably respond only to direct light or metabolic inputs. In other words, would it be possible to identify a 'contribution of the clock' to the photoperiodic responses described in figures 7, 8 or 9?

This is a good point raised by the editors, in the current version of the manuscript we have included comments regarding circadian genes in figures 7, 8 and 9 and those genes specifically losing rhythmicity under constant light or darkness discussing the inputs from the circadian clock and light regulation.

The authors should consider that the circadian clock and photoreceptors, some described in the supplemental Figure 4, are at the heart of photoperiod regulation in plants.

Indeed we agree with the editors and have included comments of the central position of these genes in the circadian clock in Ostreococcus tauri possibly controlling the response to photoperiodic variations.

- Intro, line 86, it would be useful to specify the evolutionary position of Ostreococcus in the green lineage. In the way is presented, the current sentence is not informative.

We have explicitly stated that: “Ostreococcus occupies a key position in the green lineage (Viridiplantae) as a representative of the basal class Mamiellophyceae, which diverged early in plant evolution and could provide insights into the ancestral traits of green plants and their evolutionary processes”

- Line 128, even if the information is reported in the methods, at the beginning of the result session, it will be important to also indicate the light/irradiation intensities and conditions used in LD and SD, and not just the information on the duration of the light/dark cycles, as the different amounts of light received by the cells will certainly play a role in the different rhythmic responses described.

We have stated, at the beginning of the results section, light intensity variation and maximum value (1500 $\mu E\ m^{-2}\ s^{-1}$) as well as the temperature (20ºC) and pH (8) used in the experiments.

- To make reading more fluid, the authors should avoid describing the results in the legends.

As suggested by the editors we have removed all comments about results in the figure legends.

- To facilitate reading, authors could consider to indicate the name of the genes also in the Supp. Figures, close to the IDs.

As suggested by the editors gene names have been included in supplemental figures next to gene ids to facilitate reading.

- In the legend of Fig. 4 ,line 1543, at the end of the text, the description of panel G is missing.

We apologize for missing the description of this panel. The description of this panel has now been included in figure 4.

- The authors may consider moving some data into the supplementary. Maybe Fig. 5A is not essential. However, if the authors consider useful to keep the information in the main text, they should make the implication of the analysis and results clearer.

We agree that this panel is not central in the results but it is still informative so we have decided to move it to a new supplementary figure 10.

- In Fig.6, Fig6A and C seem to provide very similar information. Are they all necessary?

Fig6 C show in a circular manner the organization of the cell cycle phases depicted already in Fig 6 A. As suggested by the editors this information may be seen as redundant and consequently we have removed panel C from this figure.

- Fig. 6B, authors need to make clearer if gene expression profiles refer only to those of Supplementaty Table 6.

This has been explicitly mentioned in the text in the new version.

- In Figure 7, some of the information in panels C and D, E, F and G appear redundant. The authors may consider retaining only the scheme in panel C and the genes/proteins as in the panels D-G .

- The same in Fig. 8

We agree with the editors that panels D-G can be redundant with panel C and have accordingly removed them from the current version of Figure 7. Similarly, we have removed panels B and D from Figure 8, panel B from Figure 9 and panel B from Figure 10. Nevertheless, we think that the organization of heatmaps in piles facilitates the comparison between the different abundance profiles and therefore we have moved all these panels to a new supplementary figure 11.

- Supplementary Tables indicating data on carotenoid and enzymatic activities of Nitrate Reductase (NR) and Glutamine Synthetase miss statistical support

We have added the statistical support for the rhythmicity of these data in separate tabs in each excel file. In supplemental_table_9 (NR and GS data) the tab is called "Enzymatic activities rhythmicity" and in supplemental_table_8 (carotenoid data) the tabs are called "LD carotenoid rhythmicity" and "SD carotenoid rhythmicity".

- Table 1 mentioned in line 235 is missing

We apologize for uploading the docx file of this table with an incorrect file type. Table 1 has now been included at the end of the main document after figure legends.

Reviewer #4 (Comments for the Author):

Comments:

1) The authors have addressed most of my questions and comments. However, I would like to highlight what I think is an important detail. In the paragraph starting in line 458 the authors suggest that low starch accumulation in batch cultured Ostreococcus is mainly caused by the constant supplementation of nitrate and CO2. Algal cells are known for accumulating starch under low nitrogen conditions and therefore it is unlikely that low N is the main limitation causing the differences. If the authors grew the cells as described by O'Neill et al., 2011 the key environmental difference is light intensity, which was 11.5 uE in O'Neill (and includes the use of a blue light filter), which is much lower than the up to 1500 uE used in this study. Light limitation is probably the main reason why the batch cultured cells have low starch. It might be important in this context to mention that Moulager et al., 2007 (PMC1914124) indicated that at higher light intensities (35 uE) Ostreoccocus looses rhythms of cell division. This was probably the reason why Ostreococcus circadian studies have used lower light intensities. The authors' results highlight that rhythmicity varies across physiological responses depending on the environmental conditions.

We agree with this reviewer that light irradiance is most likely causing the differences observed in starch content. As suggested, we have included the light irradiance values in our conditions and in O'Neill when discussing this in the current version of the manuscript. Moulager et al., 2007 is included in the references accordingly.

2) I could not find Table 1.

Table 1 has now been included at the end of the main document after figure legends.

3) Minor comments:
a. Text spacing is still off for many in text citations

We have again checked this and found it without spacing in our version. The observed spacing could be produced during the generation of the pdf merging all the uploaded files.

b. Line 253: a reference is needed on transcriptional regulation by CCA1

We have added the following reference to support this sentence as suggested:
Kamioka, M., Takao, S., Suzuki, T., Taki, K., Higashiyama, T., Kinoshita, T., Nakamichi, N. (2016) Direct repression of evening genes by CIRCADIAN CLOCK-ASSOCIATED1 in the Arabidopsis circadian clock. Plant Cell 28:696–711.

c. Line 255-257: the authors seem to suggest that the binding of OtCCA1 to potential target genes changes 8-12 h between LD and SD. Is that supported by the transcriptome and/or proteomic data?

The presence of the EE in the promoters of genes that, according to our transcriptomic data, exhibit expression peaks at different time points under LD and SD conditions could indicate that OtCCA1 binding to this DNA sequence could be involved in this differences in gene phases. The text in the current version of the manuscript has been edited to clarify this point.

Reviewer #5 (Comments for the Author):

The manuscript has certainly improved. Although it remains dense and challenging to read, there have been clear efforts to integrate the datasets (TFBS and sPLS analysis) and to improve the text.

I believe the manuscript deserves publication in The Plant Cell due to the quality and thoroughness of its experiments and data analysis. The authors have addressed my comments satisfactorily (and seemly those of the other reviewers).

We would like to thank this reviewer for his/her constructive comments throughout the entire reviewing process which has definitely improved our manuscript. We have made an effort to improve the readability of the manuscript, specially in Figure legends and the Discussion section in the current version of our paper. We have also addressed this reviewer's comments below:

Below, I have included a few minor comments.

- Line 96: approaches

We would like to thank this reviewer for spotting this typo that has now been corrected in the current version of the manuscript.

- Lines 138/205: Seasonal Variations - The authors should avoid using the term "seasonal variation" throughout the experiments. Please replace it with "photoperiodic variations."

We agree with this reviewer and, as suggested, we have replaced "seasonal variations" with "photoperiodic variations" in the current version of the manuscript.

- Line 662: Circatidal rhythms oscillate with a 12.4-hour periodicity. If these bimodal oscillations were circatidal, one would expect a daily phase shift of approximately one hour (which does not seem to be observed here).

The reviewer is totally right in this point and this comment has now been included when discarding the circatidal nature of the observed 12h periodic rhythmic genes.

- Figure 11: The figure lacks clarity. The legends (e.g., S, Viola, Prasi) do not sufficiently explain the diagram, limiting the reader's understanding of its meaning.

We have updated the legend of this figure to clarify the meaning of each label.

Reviewer #6 (Comments for the Author):

I was asked to replace reviewer 1, who apparently was unavailable to re-review the revised manuscript. I read the manuscript, as well as the reviewers' comments, especially those of reviewer 1.
Below I briefly summarize my own impressions of the revised manuscript and also how this addresses the comments of reviewer 1.

Romero-Losada et al. provide a large combined transcriptomic and proteomic study of the marine algae Ostreococcus tauri, a model system that may not be highly abundant in marine environments, but due to its accessibility has become an important reference system for marine chronobiology. The authors study how their -omics results differ between different photoperiods and constant light conditions (both LL and DD).
Technically, the work is well performed, as already remarked by the previous reviewers.

Major points:

1.)
Conceptually, I cite the initial summary of reviewer 1: "While the generated dataset is unique and present a great resource for the research community, the Results section is very descriptive. A missed opportunity is that no/limited new insights are reported about e.g. the mechanisms of the underlying gene regulation (transcriptional and/or at the protein level)." This has not changed in my opinion. In fact, also the discussion section is basically a repetition of the results. If the manuscript is meant to go beyond a mere resource, the authors should use the space to derive new insights from their data. Several reviewers, including reviewer 1, suggested to put the data into the context of work from other species (quotation reviewer 1: "an in-depth cross-species analysis has the potential to uncover new findings about e.g. conserved genes or functional features, which could generate new testable hypotheses."). The request for an "in-depth analysis" is not sufficiently addressed by simply citing the paper mentioned by reviewer1.

2.)
Related to the comment above- the main findings of the work are that
a) phases and amplitudes can change between SD, LD, LL and DD.
But what is specifically novel about these findings? Phases changes between SD and LD have long been described for other species.
For LL and DD, the endogenous oscillator is in free-run, meaning it should run by its endogenous period, which is not exactly 24hrs. This automatically implies that there should be phase changes between the entrained conditions and the free-running conditions. Also, it is well established that light intensity under constant conditions has an impact on the circadian clock period length and synchrony (Aschoff, J. Cold Spring Harb Symp Quant Biol. 1960; 25:11-28)- thus, differences between the phases are expected under these free-running conditions. What are the relevant specific novelties and new testable hypothesis derived from this?
b) Transcriptomic and proteomic peaks don't necessarily correlate.
Again, this by itself is a well-established (and published) fact (e.g. https://doi.org/10.1093/bioinformatics/btt537; https://doi.org/10.1186/s12864-019-5749-3).
What are the relevant specific novelties and new testable hypothesis derived from this?
c) The proteome cycles less than the transcriptome.
This could be meaningful. However, in the absence of another hypothesis, this could be relatively easily explained by the fact that proteins are often more stable than transcripts. Total protein abundance had been measured (in my understanding), hence if the overall

protein turnover is low, this would mask cycling, although the actual synthesis (and degradation) of a given protein could be rhythmic. What would be a more mechanistically interesting hypothesis?
d) The cycling of core circadian clock components, such as cca1 and toc1, under DD, which represents a discrepancy to the work of O'Neill et al, 2011. The authors aim to explain this difference by the different starch content, but it leaves me puzzled as to how low starch content could explain a stop of the core circadian clock or a total desynchronization of the cellular population? If the authors elaborated further on this and integrated known work, this could lead to relevant and testable new hypotheses.

3.) Plotting of individual transcripts- it is unclear if the plot represents a single BR, the mean of all BRs, the median of all BRs? Please indicate what is plotted and also include the range of biological variability. This is particularly relevant when phases are compared, like in Fig.2C,F.

The other major and minor comments of reviewer 1 were all sufficiently addressed in my opinion.

We would like to thank this reviewer for taking over the task of the previous one and dedicating time and effort to improve our manuscript.

We have followed all his suggestions regarding how to make clear in the discussion section the implications and novelty of our study. As indicated by this reviewer all other major and minors points raised previously have been addressed.

Dear Dr. Francisco Romero-Campero:

We are pleased to inform you that your paper entitled "Unveiling Photoperiodic Plasticity in Molecular Rhythms in Marine Phytoplankton through Multiomics Integration" has been accepted for publication in The Plant Cell, pending a final editorial review by a science editor. At this stage, your manuscript will be evaluated by a science editor with respect to its presentation of scientific content, compliance with journal policies, and presentation for a broad readership. The Plant Cell has contracted with Plant Editors (planteditors.com) to provide this service to our authors, and you will soon receive additional information on this process.

As there has been a major reorganization of the Figures in the course of the revision, we recommend a careful reading of the legends; inconsistencies have been found, for example, in Figure 8 and Figure 8 legends.

Please note that each author needs to link their ORCID identifier to their account in the system before your manuscript can be published. If any authors do not have an ORCID linked to their account, they will receive a message with a link to complete this task. Please ensure that ALL of your coauthors have completed this task as soon as possible.

LICENSE INFORMATION Shortly after your paper is sent to production, you will receive an email from our publisher's author support team, **SciPris**, with information on article processing charges, as well as the types of Open Access license you would be able to purchase. The accepted version of your manuscript will not appear on our Advance Articles page until the license is selected and signed, so please look out for an email from SciPris. For more information see the Article Publication Fees section of our author guidelines: https://academic.oup.com/plcell/pages/General-Instructions

The Plant Cell and The Arabidopsis Information Resource (TAIR) are collaborating to collect functional annotation data about Arabidopsis genes from authors. This includes information about the gene's molecular function (e.g., kinase activity, ATP synthetase activity), the biological process/es it is involved in (e.g., endosperm development, threonine biosynthesis), its subcellular location (e.g., nucleus, ER), anatomical or developmental expression pattern (e.g., leaf, ovule, flower stage 10, seedling stage), or its partner in a protein-protein interaction (e.g., AT1G01010 interacts with AT1G01020).

If your paper contains results falling into one or more of these categories for Arabidopsis genes, we request that you now submit these data for inclusion in TAIR by filling in the form provided at the following URL: https://www.arabidopsis.org/doc/submit/functional_annotation/123. If you need further clarification on what types of data can be submitted please contact curator@arabidopsis.org.

Finally, we encourage your submission of artwork for the journal cover. If there is an image or illustration related to your paper that you would like to have considered, please email it in .pdf or .tif format to clowe@aspb.org. An image title, brief, 2-3 sentence description of the image, and the name of the person credited for the image are also encouraged. If your image is selected to appear on the online cover of the journal, you will be contacted with the scheduled issue date and further information.

We look forward to seeing your paper published.

On behalf of the editorial board,

Angela Falciatore, Board of Reviewing Editors
Pablo Manavella, Incoming Editor-in-Chief
The Plant Cell
Feliz Navidad!

=====
IMPORTANT REMINDER: PEER REVIEW REPORTS
=====

If you opted to publish a peer review report along with your article during the original submission process, it will be prepared by the editorial staff and publicly posted with your manuscript, inside the zip file that contains any other supplemental material. As a reminder, the peer review report is a public record of all comments from editors and reviewers, as well as your prior responses, as you received them in the decision letters for each draft of your manuscript. If you agreed to publish this report and have changed your mind, or are not sure if you selected this option, please contact the editorial office as soon as possible before signing the license agreement from our publisher.

----FOR ASPB OFFICE USE ONLY (DO NOT EDIT)----
MSID: 39972
Corresponding Author: Dr. Romero-Campero
